# Identification, Characterization and Quantification of Process-Related and Degradation Impurities in Lisdexamfetamine Dimesylate: Identifiction of Two New Compounds

**DOI:** 10.3390/molecules23123125

**Published:** 2018-11-29

**Authors:** Shenghua Gao, Lili Meng, Chunjie Zhao, Tao Zhang, Pengcheng Qiu, Fuli Zhang

**Affiliations:** 1 Shanghai Institute of Pharmaceutical Industry, China State Institute of Pharmaceutical Industry, No. 285 Gebaini Road, Shanghai 201203, China; shh_gao@163.com (S.G.); 18221816872@163.com (L.M.); zt0414@126.com (T.Z.); 2 College of Pharmacy, Shenyang Pharmaceutical University, Shenyang 110016, China; zcjjljj@sina.com

**Keywords:** lisdexamfetamine dimesylate, impurities, structural elucidation, forced degradation, HPLC validation

## Abstract

Twelve impurities (process-related and degradation) in lisdexamfetamine dimesylate (LDX), a central nervous system (CNS) stimulant drug, were first separated and quantified by high-performance liquid chromatography (HPLC) and then identified by liquid chromatography mass spectrometry (LC-MS). The structures of the twelve impurities were further confirmed and characterized by IR, HRMS and NMR analyses. Based on the characterization data, two previously unknown impurities formed during the process development and forced degradation were proposed to be (2*S*)-2,6-di-(lysyl)-amino-*N*-[(1*S*)-1-methyl-2-phenyl ethyl]hexanamide (Imp-H) and (2*S*)-2,6-diamino-*N*-[(1*S*)-1-methyl-2-(2-hydroxyphenyl)ethyl] hexanamide (Imp-M). Furthermore, these two compounds are new. Probable mechanisms for the formation of the twelve impurities were discussed based on the synthesis route of LDX. Superior separation was achieved on a YMC-Pack ODS-AQ S5 120A silica column (250 × 4.6 mm × 5 μm) using a gradient of a mixture of acetonitrile and 0.1% aqueous methanesulfonic acid solution. The HPLC method was optimized in order to separate, selectively detect, and quantify all the impurities. The full identification and characterization of these impurities should prove useful for quality control in the manufacture of lisdexamfetamine dimesylate.

## 1. Introduction

Lisdexamfetamine dimesylate (LDX; formerly NRP-104), (2*S*)-2,6-diamino-*N*-[(1*S*)-1-methyl-2-phenylethyl]hexanamide dimethanesulfonate) is a novel, long-acting, central nervous system (CNS) stimulating drug with low toxicity used as an abuse-resistant treatment of attention-deficit/hyperactivity disorder (ADHD). LDX is a therapeutically inactive amphetamine prodrug, and the pharmacologically active d-amphetamine is gradually released by rate-limited hydrolysis following ingestion [1]. The drug, originally developed by Shire Development Inc. (London, UK) and New River Pharmaceutical Inc. (Washington, DC, US) is currently marketed under the trade name of Vyvanse since its launch in February 2007 [2,3].

**Figure 1:**
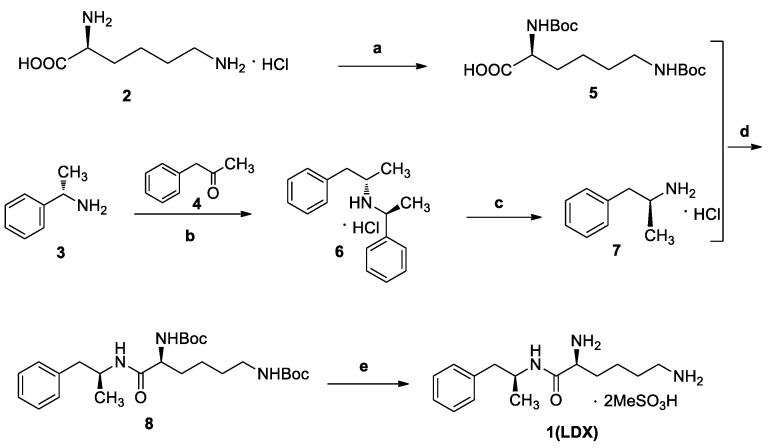
The synthesis route of LDX. *Reagents and conditions*: (**a**) Boc_2_O, acetone/2N NaOH, 25 °C, 4 h, 94%–98%; (**b**) (*i*) NaBH(AcO)_3_, DCM, rt., 9h; (*ii*) THF, 36% hydrochloric acid, 73%–78%; (**c**) ammonium formate, MeOH, 65 °C, 3 h, 92%–96%; (**d**) (*i*) EDCI, HOBt, NMM, DMF, rt., 20 h, 92–95%; (*ii*) recrystallization (acetone:*n*-heptane = 1:10, *v*/*v*); (**e**) MeSO_3_H, THF, 50 °C, 6 h, 95%–96%.

The industrial manufacturing process of LDX was developed by New River Pharmaceutical Inc. (Figure 1) [2]. Impurities in drugs are closely related to their adverse reactions and pharmacological activity. For example, degradation products, precursors, and byproducts in drugs can produce fatal immune responses, which may be responsible for some clinical allergic reactions [4,5]. After a comprehensive literature survey, we found that only one patent cursorily referred to six impurities of LDX [3]. Unfortunately, there was no information about the synthesis and spectroscopic data of LDX process-related and degradation impurities. There was only one analytical method available for quantitative analysis of LDX in the literature [6]. However, the paper only focused on comparison of CAD and UV detectors, but did not include information on process-related impurities of LDX. Furthermore, we did not get good separation resolution between LDX and process-related impurities according to the literature. According to the guidelines recommended by the International Conference on Harmonization (ICH), impurities present in drug substances exceeding the accepted level of 0.1% should be identified and characterized [7]. Hence, a thorough study was conducted to develop an effective and sensitive method for separation and identification of impurities in LDX. 

This study aimed to: (1) identify impurities formed during the preparation of LDX and its forced degradation study; (2) characterize and confirm structures of process-related and degradation impurities by IR, HRMS and NMR. The impurities were proposed based on the molecular weight revealed by LC-MS, and confirmed by their synthesis followed by spectroscopic analysis; (3) develop an effective and sensitive HPLC method to separate and quantify all the related substances of LDX. To our knowledge, this is the first comprehensive study on process-related and degradation impurities in LDX including their characterization and probable mechanisms of formation, and on development of an effective HPLC method to separate and quantify them.

## 2. Results and Discussion

### 2.1. Detection of Process-Related Impurities and Forced Degradation of LDX

**Figure 2:**
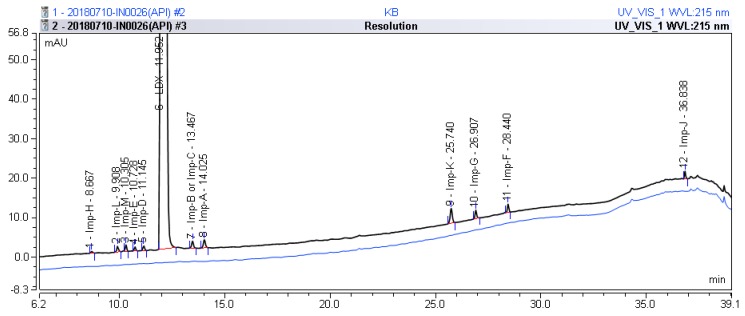
Typical HPLC chromatogram of LDX spiked with its impurities.

After analysis of different laboratory batches of LDX, process-related impurities were detected in the range of 0.05–1.61%. The HPLC method described in Section 3.2 was used to obtain a typical LC-UV chromatogram of a bulk drug sample of LDX, presenting eleven peaks (the retention time of Imp-B and Imp-C was the same due to their being enantiomers), Imp-H (RT = 8.667, relative retention time (RRT) = 0.725); Imp-L (RT = 9.908, RRT = 0.828); Imp-M (RT = 10.305, RRT = 0.862); Imp-E (RT = 10.728, RRT = 0.897); Imp-D (RT = 11.145, RRT = 0.932); Imp-B and Imp-C (RT = 13.467, RRT = 1.126); Imp-A (RT = 14.025, RRT = 1.173); Imp-K (RT = 25.740, RRT = 2.153); Imp-G (RT = 26.907, RRT = 2.251), Imp-F (RT = 28.440, RRT = 2.379); Imp-J (RT = 36.838, RRT = 3.082), which were shown in Figure 2. Moreover, the LDX samples were analyzed by LC-MS and the molecular weights were 135.1 (Imp-A), 263.2 (Imp-B and Imp-C), 391.2 (Imp-D), 391.2 (Imp-E), 363.2 (Imp-F), 363.2 (Imp-G), 519.3 (Imp-H), 463.3 (Imp-J), 163.1 (Imp-K), 249.2 (Imp-L) and 279.2 (Imp-M), respectively (Figure S5). 

During the course of degradation studies (under acidic, thermal and photolytic conditions), no significant change in the sample purity was observed. However, three degraded products (Imp-A, -B and -C) under alkaline and one degradation product (Imp-M) under oxidative conditions were detected.

### 2.2. Impurity Preparation and Structural Confirmation

All twelve LDX impurities were synthesized in our laboratory and further confirmed by IR, HRMS, NMR, and MS/MS spectroscopy. The HRMS data and carbon atom numbering scheme were shown in Table 1 and the ^1^H-NMR and ^13^C-NMR spectral data of the impurities were shown in Table 2 and Table 3, respectively. There were detailed descriptions on the structural characterization of (1*S*)-1-phenyl propan-2-amine (Imp-A) [8,9], (2*S*)-2,6-di-((*tert*-butoxycarbonyl)amino)-*N*-[(1*S*)-1-methyl-2-phenyl ethyl]hexanamide (Imp-J) [10] and *N*-[(1*S*)-1-methyl-2-phenylethyl]formamide (Imp-K) [11] in the literature. Accordingly, the structures of Imp-A, Imp-J and Imp-K were confirmed by comparison with published spectral data. All the relevant spectral data for structural confirmation are shown in the Supporting Information.

#### 2.2.1. Structural Elucidation and Control Strategy of Imp-B and Imp-C

**Figure 3:**
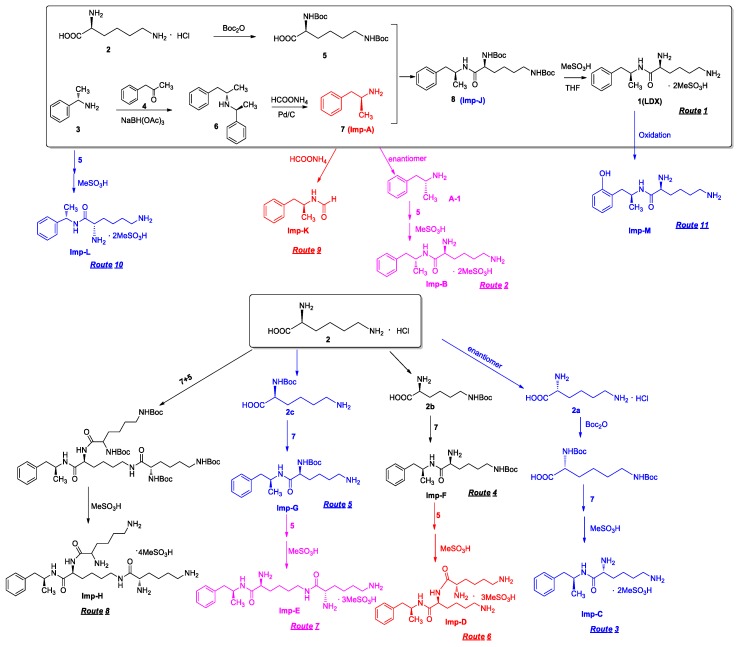
Eleven routes for the formation of LDX and its impurities.

Imp-B and Imp-C originated from the enantiomers of two different starting materials **2, 3**. The synthetic route of Imp-B and Imp-C were consistent with LDX (Figure 3), except that l-lysine (**2**) was replaced by d-lysine (**2a**) or (*S*)-1-phenylethanamine (**3**) was replaced by (*R*)-1-phenylethanamine (**3a**). Imp-B and Imp-C, were obtained as white solids and their HPLC purities were 98.63% and 96.71%, respectively. The HRMS of Imp-B and Imp-C showed an [M + H]^+^ at *m*/*z* 264.2071 and 264.2069, respectively, suggesting the same elemental composition of C_15_H_26_N_3_O (Table 1) as LDX. Imp-B and Imp-C were at the same position in reversed-phase liquid chromatography but were displayed as two different peaks (RT = 12.740 min and 14.288 min) in normal-phase chromatography (in Supporting Information Figure S6), which indicated that they were isomers instead of an identical compound. Specific rotations of Imp-B and Imp-C were +6.512 and −6.847, respectively, further supporting that Imp-B and Imp-C, with identical molecular formulae, were diastereoisomers of LDX. Detailed ^1^H-NMR spectral data were given in Table 2. The control strategy of Imp-B and Imp-C was to minimize the isomers of intermediate **8** by recrystallization (acetone:*n*-heptane = 1:10, *v*/*v*). Furthermore, by means of recrystallization of LDX, Imp-B and Imp-C were easily removed leaving less than 0.1% content in the bulk drug.

#### 2.2.2. Structural Elucidation and Control Strategy of Imp-F and Imp-G

Both of the protonated molecular ions for Imp-F and Imp-G, were obtained at an [M + H]^+^ of *m/z* 364.2 (Figure S5), which was 100 a.m.u. more than that of LDX. The 100 may correspond to one *t*-butyloxy carbonyl (Boc) moiety and thus we speculated that the two impurities were possibly (2*S*)-2-amino-6-((*tert*-butoxycarbonyl)amino)-*N*-[(1*S*)-1-methyl-2-phenylethyl]hexan-amide (Imp-F) and (2*S*)-2-((*tert*-butoxycarbonyl)amino)-6-amino-*N*-[(1*S*)-1-methyl-2-phenyl-ethyl]hexanamide (Imp-G), respectively. According to the synthetic route (Figure 3), Imp-F was obtained as a colorless oil with 97.63% HPLC purity while Imp-G was obtained as a white solid with 98.63% HPLC purity. 

The HRMS of impurities F and G showed an [M + H]^+^ at *m/z* 364.2595 and 364.2590, respectively, suggesting an identical elemental composition of C_20_H_34_N_3_O_3_ (Table 1). The structures were further confirmed by the IR, ^1^H-NMR, ^13^C-NMR, and DEPT spectra. Both of these impurities had one additional *t*-butyloxycarbonyl compared with LDX. In the ^13^C-NMR of Imp-F, the additional Boc group was deshielded to δ_C20_ = 77.73 ppm and δ_C21_ = 28.74 ppm. In the ^1^H-NMR, the chemical shift of the additional Boc was deshielded to δ_H21_ = 1.37 ppm. The NMR spectrum of Imp-G was similar to that of Imp-F, except that H-2 was appeared at a lower field of the ^1^H-NMR spectrum (chemical shift of δ_H_ = 3.98 ppm) that impurity G, which was affected by the acyl-amino groups at C1 and C19. This phenomenon also ocurrs between **2b** and **2c** (Supporting Information Figure S7). Detailed information about the ^1^H-NMR and ^13^C-NMR spectra can be seen in Table 2 and Table 3. To the best of our knowledge, this is the first report of all the spectroscopic data of Imp-F and Imp-G.

There were two ways whereby Imp-F and Imp-G can be formed in the bulk drug. First, intermediate **5** may contain **2b** and **2c** if the amino protection was not complete during its synthesis, affording, respectively, Imp-F and Imp-G. Second, the presence of Imp-J in LDX drug substance indicated that Imp-F and Imp-G were also likely formed due to the incomplete de-Boc in the final step of LDX. Accordingly, the control strategy of Imp-F and Imp-G was to increase the equivalents of (Boc)_2_O to 2.2 during the amino protection, so that l-lysine reacted completely as far as possible, thereby reducing the content of **2b** and **2c**. Moreover, the amount of methanesulfonic acid was increased to 5 equivalents in the last step to ensure he complete deprotection. The content of Imp-F, Imp-G and Imp-J can thus be reduced to below 0.1% after recrystallization.

#### 2.2.3. Structural Elucidation and Control Strategy of Imp-D and Imp-E

On-line LC-MS spectra of Imp-D and Imp-E in LDX suggested that they were likely (2*S*)-2-lysyl-6-amino-*N*-[(1*S*)-1-methyl-2-phenylethyl]hexanamide (Imp-D), and (2*S*)-2-amino-6-lysyl-*N*-[(1*S*)-1-methyl-2-phenylethyl]hexanamide (Imp-E), both of which were colorless oils with ≥96% HPLC purity. The HRMS of impurity D and E revealed their [M + H]^+^ at *m/z* 392.3205 and 392.3207, respectively, suggesting that they share the same elemental composition of C_21_H_38_N_5_O_2_ (Table 1). Their structures were further confirmed by IR, ^1^H-NMR, ^13^C-NMR, DEPT, HSQC and HMBC spectral data. The ^1^H-NMR spectra of Imp-D and Imp-E showed 17 signals, corresponding to 49 protons, which were consistent with the molecular structures of Imp-D and Imp-E. Both of them had an additional l-lysine on different amino groups (H17 and H18) compared with LDX. In the ^13^C-NMR spectrum of Imp-E, the chemical shift of the additional l-lysine (C20, C21, C22, C23, C24, C25) was deshielded to δ_C_ = 168.64, 52.53, 31.07, 21.64, 28.83, 38.96 ppm, respectively. The ^13^C-NMR spectrum of Imp-D was similar to that of Imp-E. In the ^1^H-NMR spectrum of Imp-D (Supporting Information Figure S7, D-1), affected by the two carbonyl groups (C1 and C20), the H-2 proton appeared at a lower field (chemical shift of δ_H2_ = 4.20 ppm) while its chemical shift is 3.60 ppm in the ^1^H-NMR spectrum of Imp-E (Figure S7, E-1). Besides, compared with the H-6 of Imp-D (δ_H6_ = 2.80 ppm), the H-6 of Imp-E, affected by the acyl-amino group (C20), shifted to a lower field (δ_H6_ = 3.02 ppm). The HSQC spectrum provided further evidence for the difference on the structures of Imp-D and Imp-E (Figure S7, D-5, E-5). In the HMBC spectrum of Imp-D (Figure S7, D-6), H-2 was correlated with C1 and C20, but the key long-range correlation between H-2 and C20 in Imp-E was not existed (Figure S7, E-6). In the meantime, there was no correlation between H-6 and C20 in Imp-D (Figure S7, D-6), while the H-6 was correlated with C20 in Imp-E (Figure S7, E-6). The correlation peaks of two-dimensional NMR spectra indicated that Imp-D and Imp-E were not the same compound, but rather positional isomers. The detailed ^1^H-NMR and ^13^C-NMR spectra information can be seen in Table 2 and Table 3. The HRMS and NMR spectra of the two impurities have never been reported in the literature.

In order to control the amount of Imp-D and Imp-E, we decreased the content of **2b** and **2c** by optimizing the process parameters in the amino protection step. In addition, intermediate **8** was recrystallized (acetone:*n*-heptane = 1:10, *v*/*v*) to reduce the precursors of Imp-D and Imp-E. As a result, the content of the two impurities in LDX were eliminated to below 0.05%.

#### 2.2.4. Structural Elucidation and Control Strategy of Imp-H

**Figure 4:**
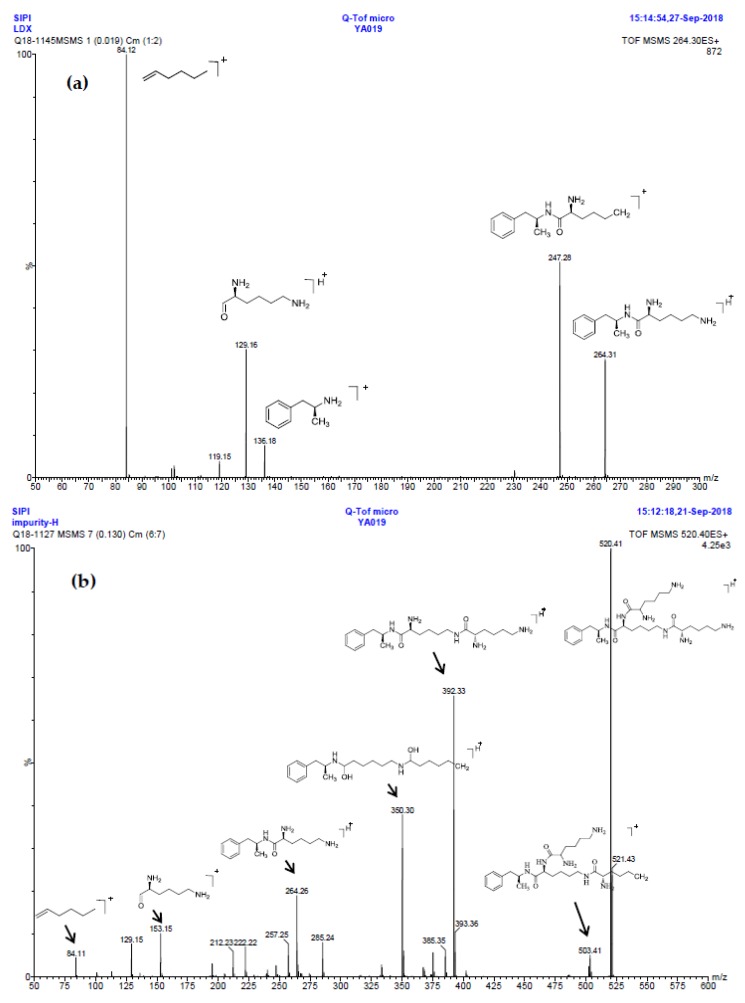

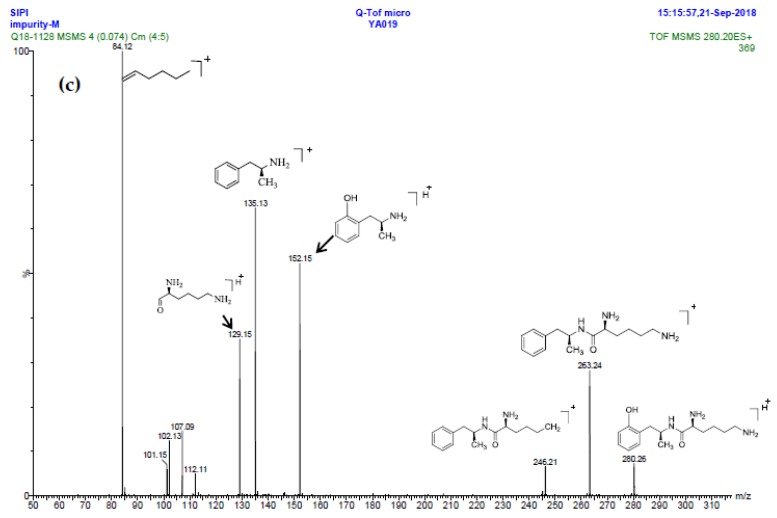
MS/MS spectra and plausible fragments of LDX (**a**), Imp-H (**b**) and Imp-M (**c**).

Inspired by the formation of Imp-D and Imp-E and a [M + H]^+^ *m*/*z* 520.3 peak for Imp-H (Figure S5), we speculated that Imp-H was (2*S*)-2,6-di-(lysyl)-amino-*N*-[(1*S*)-1-methyl-2-phenylethyl] hexanamide. Imp-H was obtained as a white solid and its HPLC purity was found to be 99.61%. By comparison of retention times in HPLC, we found that the quantity of Imp-H was about 0.05% in the bulk drug (Figure 2). The HRMS of impurity H revealed an [M+H] ^+^ at *m*/*z* 520.3975, suggesting an elemental composition of C_27_H_50_N_7_O_3_ (Table 1). Besides, the fragments 503.4, 392.3, 385.3, 264.2, 257.2, 129.1 appeared in the MS/MS spectrum of Imp-H, which supported the proposed molecular structure (Figure 4). The structure was further confirmed by IR, 1D NMR (^1^H, ^13^C, DEPT) and 2D NMR (COSY, HSQC, HMBC) spectral data. The IR spectrum displayed characteristic absorptions at 3431.0, 1671.4, 1555.5 and 1192.9/cm which were indicatives of an amino (N-H) stretching vibration, a C=O stretching vibration, an N-H bending vibration, and a C-N stretching vibration, respectively. Imp-H had two additional acyl-amino groups (-CONH) and two additional amino-groups (-NH_2_) compared with LDX. The chemical shift of the additional active hydrogens (H17, H18) were deshielded to δ_H_ = 7.8–8.5 ppm compared with the ^1^H-NMR spectrum of LDX. The additional amino groups were assigned to be H-27, H-30, H-26, H-35 on the basis of the HMBC spectrum in the Supporting Information (Figure S7) showing correlations of H-27, H-30 (δ_H_ =8.08 ppm) with C-20, C-28 (δ_C_ = 168.69, 168.63 ppm) and correlations of C-20, C-28 (δ_C_ = 168.69, 168.63 ppm) with H-17, H-18 (δ_H_ =8.43–8.42 ppm) (Figure S7, H-5). The above results indicated that Imp-H had two additional l-lysines compared with LDX. Furthermore, the H-2 and H-6 signals appeared at a lower field in the ^1^H-NMR spectrum (chemical shift of δ_H2_ = 4.18 ppm and δ_H2_ = 3.04 ppm, respectively) of impurity H (Figure S7), which indicated that the amino-group (-NH_2_) was transformed to an acylmino (-CONH). The COSY spectrum showed correlation of H-17 (δ_H_ = 8.42 ppm) with H-2 (δ_H_ = 4.18 ppm) and correlation of H-18 (δ_H_ = 8.43 ppm) with the methylene H-6 (δ_H_ = 3.04 ppm). In the meantime, there were twelve more carbon atoms in Imp-H than in LDX, and the chemical shifts of δ_C28_ = 168.69 ppm, δ_C20_ = 168.63 ppm and δ_C1_ = 170.61 ppm in the ^13^C-NMR spectrum provided further evidence for the existence of amides. The assignment of ^1^H- and ^13^C-NMR signals was performed for Imp-H on the basis of the ^1^H-, ^13^C- and 2D NMR data in Table 2 and Table 3. Further detailed information of the HSQC, HMBC and COSY spectra of Imp-H can be seen in Figure S7. To our knowledge, this compound is reported for the first time. The control strategy of Imp-H is identical to that of Imp-D and Imp-E. 

#### 2.2.5. Structural Elucidation and Control Strategy of Imp-L

The synthetic route of (2*S*)-2,6-di-amino-*N*-((1*S*)-phenylethyl) hexanamide (Imp-L) was similar to that of LDX (Figure1), except that Imp-A was replaced by (*S*)-1-phenylethylamine (**3**) in the amide condensation step. Imp-L was obtained as a white solid and its HPLC purity was found to be 98.63%. The HRMS of Imp-L revealed an [M + H]^+^ at *m*/*z* 250.1908, which suggested an elemental composition of C_14_H_24_N_3_O (Table 1). Compared with LDX, Imp-L was missing a -CH_2_ group. In the ^1^H-NMR spectrum, no benzyl (H7) was found at δ_H_ = 2.6–2.9 ppm and the chemical shift of H_8_ was deshielded from 1.14–1.15 ppm to 1.37–1.39 ppm. Detailed information about the ^1^H-NMR and ^13^C-NMR spectra is given in Table 2 and Table 3. The IR spectrum of Imp-L displayed characteristic absorptions at 3449.5, 1597.1 and 1198.5/cm which were indicative of an amino (N-H) stretching vibration, a C=O stretching vibration and a C-N stretching vibration, respectively.

The residue of (*S*)-1-phenylethanamine (**3**) in intermediate **7** led to Imp-L. Hence, the control strategy of Imp-L was to make **3** react as completely as possible in the reductive amination reaction. Thus, the equivalent ratio of (*S*)-1-phenylethanamine (**3**) and phenylacetone (**4**) was set to 1:1.1. On the other hand, the chemical and optical purity of intermediate **6** were improved by salt formation with hydrochloric acid, thereby reducing the production of Imp-L from the source.

#### 2.2.6. Structural Elucidation and Control Strategy of Imp-M

Oxidative degradation was performed in 5% H_2_O_2_ at room temperature in the dark for 4h. Considering that the purpose of the degradation experiment is to provide recommendations for transport and storage of drugs, we focused on the impurity with the maximum content under this oxidative condition. The on-line LC-MS spectrum indicated that the molecular weight of the major degradation product was 279.2 (Figure S5), 16 more than LDX. The HRMS of impurity M showed an [M + H]^+^ at *m*/*z* 280.2020, suggesting an elemental composition of C_15_H_26_N_3_O_2_ (Table 1). Furthermore, there was only four hydrogen atoms on the benzene ring accorded with the ^1^H-NMR spectrum. In other words, impurity M had more than one substituent group on the benzene ring and not the two primary amines of LDX. It was supposed that the two primary amines had formed salts with methanesulfonic acid, making them more stable in hydrogen peroxide. Furthermore, the identical oxidative experiment had been conducted with the free base of LDX, but the degraded products showed different retention time with Imp-M in HPLC. Moreover, it was reported that hydrogen peroxide with strong acid or Lewis acid converted benzene and alkylbenzenes into their hydroxylated products [12]. On the basis of molecular weight (279.2), we speculated that the additional group was a hydroxyl group. In addition, the ^1^H-NMR spectrum showed that there were four different kinds of hydrogen on the benzene ring in the low field. Thus, we excluded the para-hydroxyl degradant. Moreover, the splitting of these four kinds of hydrogen are double and triple peaks, but not single, which indicated the hydroxyl group was not located in the *meta*-position. The HMBC spectrum of Imp-M in Supporting Information Figure S7 showed correlations of the additional hydroxyl group (H-19) (δ_H_ =9.43 ppm) with C-10, C-14 and C-15 (δ_C_ = 125.30, 115.25, 155.85 ppm) and correlations of H-9 (δ_H_ = 2.73, 2.56 ppm, dd) with C-10, C-11 and C-15 (δ_C_ = 125.30, 131.29, 155.85 ppm). The above results supported that the additional OH was located in the *ortho*-position. The structure was further confirmed by ^13^C-NMR and DEPT. In the ^13^C-NMR, the carbon atom connecting to the additional OH shifted to the low field (δ_C15_ = 155.85 ppm) compared to that of LDX. In the meantime, the DEPT spectrum showed that only four carbons appeared in the aromatic region (110–160 ppm), and the C-15 (δ_C15_ = 155.85 ppm) disappeared, which confirmed again that the OH was on the benzene ring. The HSQC spectrum of Imp-M (Figure S7, M-5) showed that there was no hydrogen atom correlated with C-10 and C-15 (δC-10 = 1125.30 ppm, δC-15 = 155.85 ppm), which provided further evidence for the above conclusion. Based on the abovementioned spectral data, the new compound was identified as (2*S*)-2,6-diamino-*N*-[(1*S*)-1-methyl-2-(2-hydroxyphenyl) ethyl] hexanamide. Fragments 263.2, 246.2, 152.1, 135.1, 129.1, 84.1 were visible in the MS/MS spectrum of Imp-M, which further supports the proposed molecular structure (Figure 4). The assignment of ^1^H- and ^13^C-NMR signals was completed by means of COSY, HSQC and HMBC spectroscopic data sets (Figure S7). The detailed information about the ^1^H-NMR, ^13^C-NMR and DEPT spectra can be seen in Table 2 and Table 3. This novel degradation product has not yet been disclosed in any other published work.

### 2.3. Possible Mechanisms for Formation of the Impurities

Taking into account the synthetic process of LDX in combination with some published research, we proposed eleven possible routes for the formation of the twelve impurities (process-related and degradation) (Figure 3). Imp-A and Imp-J were the residues of intermediate **7** and intermediate **8** in the synthetic process of LDX in route 1. In routes 2 and 3, both intermediate **7** and l-lysine (**2**) contained trace amounts of enantiomers **A-1** and **2a**. The synthetic routes of Imp-B and Imp-C were consistent with that of LDX (Figure 1), except that l-lysine (**2**) was replaced by d-lysine (**2a**) while intermediate **7** was replaced by (*R*)-1-phenylpropan-2-amine (**A-1**). On the other hand, LDX can produce Imp-B or Imp-C in alkaline condition. In routes 4 and 5, intermediate **5** may contain (2*S*)-2-amino-6-((*tert*-butoxycarbonyl)amino)hexanoic acid (**2b**) and (2*S*)-6-amino-2-((*tert*-butoxycarbonyl)-amino)hexanoic acid (**2c**) as impurities when the amino protection was not completed during its synthesis, affording, respectively, Imp-F and Imp-G which, generated Imp-D (route 6) and Imp-E (route 7) after sequentially reacting with intermediate **5**, and both underwent the same reaction that gave LDX. In route 8, intermediate **5** may contain the residue of l-lysine (**2**) in its synthesis process, affording Imp-H with the same reaction that gave LDX in the last step. In route 9, during the debenzyl reaction, excessive amounts of ammonium formate may continuously react with intermediate **7**, affording Imp-K as a residue in LDX. In route 10, as an impurity, **3** might exist in intermediate **7**, and Imp-L was obtained by the same reaction for LDX. In route 11, Imp-M was produced under oxidative condition.

### 2.4. Optimization of the HPLC-UV Method

According to the foregoing analysis, twelve impurities were detected and successfully identified by LC-MS, HRMS, NMR and IR spectroscopy. Initially, different types of HPLC columns, such as Thermo Accucore XL C8 (150 × 4.6 mm, 4 μm) column, Thermo Syncronis C18 (250 × 4.6 mm × 5 µm) column and YMC-Pack ODS-AQ (250 × 4.6 mm × 5 μm) were tested to analyze LDX. The capability of separating LDX and its impurities was evaluated mainly through the performance characteristics of the columns. The best resolution was obtained on the discovery YMC-Pack ODS-AQ column which was thereafter used for further optimization of the method. 

Different mobile phase conditions and gradient progress were tested together to develop a selective separation method. We used a variety of organic acids and the tailing peak was found to appear when trifluoroacetic acid was used as the mobile phase. Fortunately, better shape symmetrical peaks were obtained with methanesulfonic acid. In the meantime, the addition of 0.1% methanesulfonic acid to acetonitrile improved the baseline fluctuation. The separation of these impurities was not satisfactory by a continuous gradient elution program. The initial gradient elution condition was as follows: 0–10 min, linear from 5% to 20% B, however, the polar impurities (D, E, L, M) cannot be well separated under this condition. For the separation of Imp-D, Imp-E, Imp-L and Imp-M, the gradient profile was optimized. On the one hand, we reduced the slope of B increase (0–15 min, linear from 3% to 20% B)**.** Alternatively, the separation can be improved by reducing the initial proportion of the organic phase to 3%. The method was initially optimized by comparing the separation of related substance, shape symmetrical peaks of LDX and its impurities, and then by optimizing the effect of column types, mobile phase and gradient elution mode afterwards shown in Section 2.2.

### 2.5. Validation of the HPLC-UV Method

The HPLC method, used to identify the related substances in LDX bulk drug, was validated in terms of the linearity, accuracy, precision, limit of quantitation (LOQ), limit of detection (LOD), robustness and system suitability. The validation was in accordance with ICH Q2 guideline [13] and the details are shown in Table 4 and Table 5.

#### 2.5.1. System Suitability

In order to obtain a satisfactory performance using the analytical method, a system suitability test was carried out before each run. The results showed that the United States Pharmacopoeia (USP) theoretical plates of LDX and its impurities were greater than 19755, the USP resolution between any two compounds was greater than 3.19, and the peak asymmetry for all the analytes was between 1.02 and 1.28 (Table 4). The HPLC chromatogram of the separation of LDX and its impurities can be seen in Figure 2.

#### 2.5.2. Linearity, LOD, and LOQ 

Using the least squares method, linear regression analysis of the response values of sample solutions with different concentrations and the corresponding concentration was carried out to calculate the slope and intercept. The measurements indicated that the response value and concentration had a positive linear relationship over the concentration range of 0.50–20.00 μg/mL. The LOQ solution (0.50 μg/mL), equivalent to 0.05% of the LDX sample solution, was prepared and used to calculate the (S/N) of LDX and its twelve impurities. S/N of LDX and its impurities was greater than 10, and the LOQ of the method was 0.05% while the minimum quantitative concentration was 0.50 µg/mL. Using the same injection, the calculated LOD of the method was 0.02% and the S/N of LDX and its impurities was 3:1. The LOQ level by injecting six individual preparations and calculating the percentage RSD of the areas. The results were shown in Table 4.

#### 2.5.3. Accuracy, Precision, and Robustness

Recovery and RSD values of sample solution at concentration levels of 0.05%, 0.10%, and 0.15% were measured in triplicate after the addition of a certain amount of the twelve impurities to LDX test solutions (1.0 mg/mL) and then the accuracy was calculated. The recovery of all the impurities was 80%–120%, confirming the acceptable good accuracy of the method (Table 5). The precision of the method was evaluated through parallel preparation of six individual 1.0 mg/mL LDX sample solution for injection and calculating the RSD for each peak. The RSD of all individual impurities was not more than 5%, indicating good precision of the method (Table 6). The robustness of the developed method was studied by changing the column temperature (30 ± 3 °C) flow rate (1.0 ± 0.1 mL/min), detection wavelength (215 ± 2 nm) of the original HPLC conditions. Under different conditions, excluding the isomer of impurities B and C, resolution between any two compounds was >1.5. Compared with the original HPLC method, difference measured values of the individual impurities in the sample solution was not more than 0.02%, suggesting excellent robustness of the method (Table 7).

## 3. Materials and Methods 

### 3.1. Chemicals and Reagents

Crude LDX and its impurities were synthesized in our laboratory. l-Lysine hydrochloride (**2**), (*S*)-1-phenylethanamine (**3**) and methanesulfonic acid were purchased from Energy Chemical Corporation (Shanghai, China). The purity of all substances was >98%. HPLC-grade methanesulfonic acid were purchased from Fisher Scientific (Waltham, MA, USA). HPLC-grade acetonitrile (ACN) was purchased from Honeywell (Newark, NJ, USA). Deionized water for preparing the aqueous phase was obtained using a water purification system and all other chemicals were of analytical grade.

### 3.2. Analytical HPLC Conditions

Studies were conducted on a Dionex Ultimate 3000 HPLC instrument (Waltham, MA, USA) equipped with a quaternary pump and a DAD detector. An analytical silica column YMC-Pack ODS-AQ S5 120A (250 × 4.6 mm × 5 μm, YMC, Nagoya, Japan) maintained at 30 °C was used for separation. Mobile phase A was 0.1% methanesulfonic acid (*v*/*v*) in water, while B was 0.1% methanesulfonic acid in acetonitrile. The HPLC gradient program was set as follows: Time (min)/% of solvent B: 0/3, 15/20, 30/50, 35/95, 37/95, 37.1/3, 45/3. The flow rate was 1.0 mL/min for a total run time of 45 min, and the detection wavelength was 215 nm. The crude LDX was accurately weighed and dissolved in the mixture of water and ACN (70:30, *v*/*v*) to obtain a test solution of 1.0 mg/mL. Samples (10 µL) were injected into the HPLC system for analysis.

### 3.3. LC-MS Conditions

LC-MS was performed on an Agilent LC/MS system consisting of an Agilent 1260-LC system equipped with a single quadruple mass detector and electrospray ionization (ESI) interface (Agilent Technologies, Santa Clara, CA, USA). The column and mobile phase composition are the same as in the HPLC analysis, except that formic acid was used instead of methanesulfonic acid. The LC gradient program was set as follows: Time (min)/% of solvent B: 0/5, 40/15, 50/50, 55/95, 60/95, 60.1/5, 65/5. The flow rate was 1.0 mL/min for a total run time of 65 min. The mass instrument was operated in positive-ion ESI mode. Optimized mass conditions are as follows: drying gas (N_2_) flow rate of 12.0 L/min, drying gas temperature 300 °C, nebulizer pressure 50 psig, capillary voltage 3.0 kV. Scans were acquired from 50 to 800 amu with a 0.1 s/scan. The high-resolution mass spectra and MS/MS were recorded on a Q-TOF micro YA019 instrument (Waters, Milford, MA, USA). 

### 3.4. NMR Spectroscopy

^1^H-NMR, ^13^C-NMR, distortionless enhancement by polarization transfer (DEPT), correlation spectroscopy (COSY), heteronuclear multiple bond correlation (HMBC), and heteronuclear singular quantum correlation (HSQC) NMR spectra were recorded on an Avance III 400 MHz spectrometer (Bruker, Karlsruhe, Germany). Solvents used were DMSO-*d_6_* or CDCl_3_.

### 3.5. FT-IR Spectroscopy

IR spectra were recorded in the solid state as KBr dispersions using a 670 FT-IR spectrophotometer (NICOLET Waltham, MA, USA). Data were collected between 400 and 4000/cm, at a resolution of 4.0/cm.

### 3.6. Preparation of Standard and Sample Solutions

Samples was prepared using a water and acetonitrile mixture (70:30, *v*/*v*) as the diluent. In each trial, the HPLC conditions were investigated by injecting test solution added with the twelve impurities into the HPLC system. The concentration of LDX sample was 1.0 mg/mL, prepared by spiking the twelve impurities (Imp-A, Imp-B, Imp-C, Imp-D, Imp-E, Imp-F, Imp-G, Imp-H, Imp-J, Imp-K, Imp-L and Imp-M) into LDX at a concentration of 1.0 μg/mL and used to investigate the system suitability.

### 3.7. Forced Degradation Study

For forced degradation solutions, LDX was subjected to stress conditions according to ICH guidelines [14]. The forced degradation of LDX was performed under hydrolytic (acidic and alkaline), oxidative, thermal and photolytic conditions. The hydrolytic degradation was carried out separately in 1.0 M HCl (2.0 mL) as well as 5.0 M NaOH (2.0 mL) and kept in water bath at 90 °C for 3 h. The oxidative degradation was performed in 5% H_2_O_2_ (5.0 mL) at room temperature in the dark for 4 h. LDX was also subjected to thermolytic (90 °C, 48 h) and photolytic (UV light, 4500 lx, 24 h) degradation. After completion of the experiment, the samples were cooled to ambient temperature, neutralized with a base or an acid, respectively. All of the stressed samples were kept at a concentration of 1.0 mg/mL for assay determination. 

## 4. Conclusions

An effective and selective HPLC method, used for the separation and determination of the twelve impurities (process-related and degradation) in LDX bulk drug, was developed and optimized. Structures of the two new compounds, Imp-H and Imp-M, were proposed by the synthesis route of LDX and LC-MS analyses, and then confirmed and characterized using HRMS, ESI-MS/MS, 1D NMR (^1^H-, ^13^C-, DEPT 135) and 2D NMR (COSY, HSQC, HMBC). Furthermore, probable mechanisms for the formation of the process-related and degradation impurities were proposed based on the synthesis route of LDX. The HPLC method was validated in terms of its linearity, accuracy, robustness, limits of detection, and quantification. Full identification and characterization of these impurities is useful in quality control in the manufacture of LDX.

## Figures and Tables

**Table 1:** Retention time, HRMS and structures of LDX and its impurities.

Compound	RRT	HRMS		Structure	Source
		[M + H]^+^	Chemical Formula		
LDX	1.00	264.2076	C_15_H_26_N_3_O	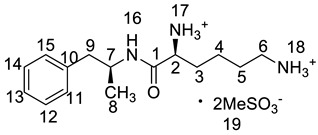	Target compound
Imp-A	1.17	136.1121	C_9_H_13_N	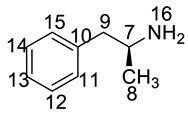	Process and alkaline degradation
Imp-B	1.12	264.2071	C_15_H_26_N_3_O	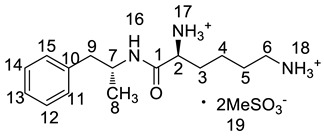	Process and alkaline degradation
Imp-C	1.12	264.2069	C_15_H_26_N_3_O	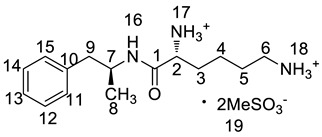	Process and alkaline degradation
Imp-D	0.93	392.3205	C_21_H_38_N_5_O_2_	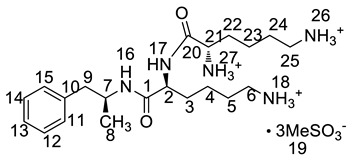	Process
Imp-E	0.89	392.3207	C_21_H_38_N_5_O_2_	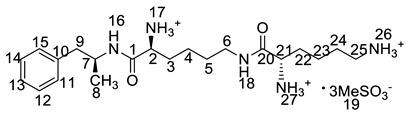	Process
Imp-F	2.38	364.2595	C_20_H_34_N_3_O_3_	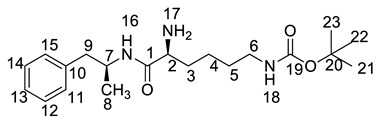	Process
Imp-G	2.25	364.2590	C_20_H_34_N_3_O_3_	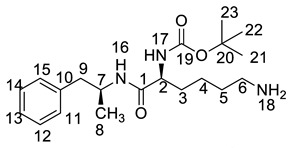	Process
Imp-H	0.72	520.3975	C_27_H_50_N_7_O_3_	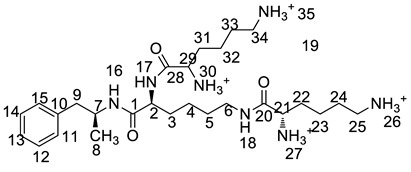	Process
Imp-J	3.08	464.3118	C_25_H_41_N_3_O_5_	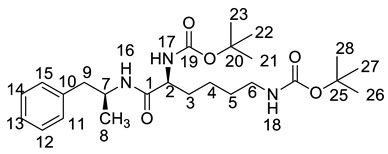	Process
Imp-K	2.15	186.0889 (M + Na^+^)	C_10_H_13_NO	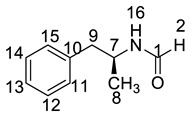	Process
Imp-L	0.82	250.1908	C_14_H_24_N_3_O	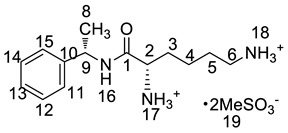	Process
Imp-M	0.86	280.2020	C_15_H_26_N_3_O_2_	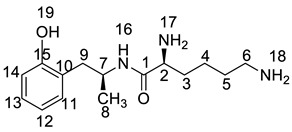	Oxidative degradation

**Table 2:** ^1^H-NMR assignment for LDX and its impurities.

Position	LDX	Imp-B	Imp-C	Imp-D	Imp-E	Imp-F	Imp-G	Imp-H	Imp-L	Imp-M
2	3.66–3.63 (t,1H)	3.67–3.63 (t,1H)	3.67–3.63 (t,1H)	4.22–4.18 (t,1H)	3.63–3.61 (t,1H)	3.10–3.07 (t,1H)	3.99–3.95 (t,1H)	4.20–4.12 (dt,1H)	4.93–4.88 (m,1H)	3.65–3.63 (t,1H)
3	1.48–1.40 (m ^(c)^,4H)	1.76–1.67 (dd ^(d)^,2H	1.76–1.68 (dd,2H)	1.79–1.69 (m,2H)	1.75–1.68 (m,2H)	1.47,1.30 (m,2H)	1.54–1.46 (m,2H)	1.49–1.43 (m,2H)	1.53–1.46 (m,2H)	1.54–1.46 (dd,2H),
4	1.03–0.92 (m,2H)	1.35–1.26 (m,2H)	1.35–1.26 (m,2H)	1.39–1.31 (m,2H)	1.38–1.27 (m,4H)	1.24–1.17 (m,2H)	1.28–1.23 (dd,2H)	1.13–1.11 (d,2H)	1.25–1.14 (m,2H)	1.08–0.86 (m,2H).
5	1.48–1.44 (m,4H)	1.59–1.47 (m,2H)	1.59–1.47 (m,2H）	1.63–1.48 (m,6H)	1.49–1.45 (m,2H)	1.34–1.26 (m,2H)	1.43–1.34 (m,2H)	1.38–1.34 (m,6H)	1.69–1.66 (m,2H)	1.44–1.39 (m,2H),
6	2.78–2.73 (m,2H)	2.78–2.71 (m,2H)	2.78–2.70 (m,2H)	2.82–2.76 (m,4H)	3.03–2.98 (m,2H)	2.89–2.84 (dd,2H)	2.69–2.61 (m,2H)	3.06–3.01 (m,2H)	2.67–2.61 (dd,2H)	2.68–2.61 (m,2H),
7	4.16–4.04 (m,1H)	3.97–3.93 (m,1H)	3.97–3.93 (m,1H)	3.97–3.92 (m,1H)	4.15–4.07 (m,1H)	3.93–4.03 (m,1H)	4.32–4.32 (m,1H)	4.02–3.95 (m,1H)	–	4.20–4.09 (m,1H),
8	1.13 (d ^(b)^,3H)	1.03 (d,3H)	1.02 (d,3H)	1.01 (d,3H)	1.14 (d,3H)	1.03 (d,3H)	1.15 (d,3H)	1.07 (d,3H)	1.38 (d,3H)	1.11–1.10 (d,3H),
9	2.65,2.75 (dd,2H)	2.62,2.82 (dd,2H)	2.62,2.82 (dd,2H)	2.62,2.79 (dd,2H)	2.68,2.76 (dd,2H)	2.77,2.63 (dd,2H)	2.84–2.71 (dd,2H)	2.69–2.67 (d,2H)	3.79 (t,1H)	2.72,2.56 (dd,2H)
11,13	7.24–7.18 (m,3H)	7.23–7.17 (m,3H)	7.23–7.16 (m,3H)	7.23–7.17 (m,3H)	7.23–7.18 (m,3H)	7.20–7.17 (m,3H)	7.24–7.18 (m,3H)	7.20–7.19 (m,3H)	7.34–7.28 (m,4H)	7.04(d,1H), 7.01(t,H)
12,14	7.31–7.26 (m,2H)	7.32–7.26 (m,2H)	7.32–7.25 (m,2H)	7.31–7.26 (m,2H)	7.30–7.26 (m,2H)	7.29–7.26 (m,2H)	7.31–7.28 (m,2H)	7.30–7.23 (m,2H)	7.34–7.28 (m,4H)	6.69(d,1H), 6.78(t,H)
15	7.24–7.18 (m,3H)	7.23–7.17 (m,3H)	7.23–7.16 (m,3H)	7.23–7.17 (m,3H)	7.23–7.18 (m,3H)	7.20–7.17 (m,3H)	7.24–7.18 (m,3H)	7.20–7.19 (m,3H)	7.34–7.28 (m,4H)	––
16	8.38–8.32 (d,1H)	8.43–8.37 (d,1H)	8.43–8.37 (d,1H)	8.01–7.99 (d,1H)	8.43–8.40 (t,1H)	7.80–7.77 (d,1H)	5.15 (d,1H)	7.99–7.97 (d,1H)	8.95–8.93 (d,1H)	8.27 (d,1H)
17	8.15–7.56 (s,6H)	8.20–8.10 (s,3H)	8.20–8.10 (s,3H)	8.65–8.63 (d,1H)	8.10–8.09 (d,3H)	–	6.16 (s,1H)	8.43–8.42 (d,2H)	8.18–8.13 (m,3H)	8.08 (s,2H)
18	8.15–7.56 (s ^(a)^,6H)	7.82–7.72 (s,3H)	7.82–7.72 (s,3H)	7.78 (s,3H)	8.33–8.30 (d,1H)	6.75–6.73 (t,1H)	–	8.43–8.42 (d,2H)	7.78 (s,3H)	7.86 (s,2H)
19	2.39 (s,6H)	2.42 (s,6H)	2.42 (s,6H)	2.39 (s,9H)	2.38 (s,9H)	–	–	2.45 (s,12H)	2.45 (s,6H)	9.43 (s,1H)
21,29				3.88–3.83 (t,1H)	3.72–3.69 (t,1H)	1.37 (s,9H)	1.45 (s,9H)	3.82–3.73 (t,1H)		
22,31				1.63–1.48 (m,6H)	1.60–1.52 (dd,2H)	1.37 (s,9H)	1.45 (s,9H)	1.72–1.70 (d,4H)		
23,32				1.25 (d,2H)	1.04,0.93 (m,2H).	1.37 (s,9H)	1.45 (s,9H)	1.38–1.34 (m,6H)		
24,33				1.63–1.48 (m,6H)	1.38–1.27 (m,4H)			1.62–1.53 (dd,4H)		
25,34				2.82–2.76 (m,4H)	2.79–2.74 (dd,2H)			2.84–2.71 (dd,4H)		
26,(35)				7.78 (s,3H)	7.70 (s,3H)			7.75 (s,6H)		
27,(30)				8.14 (s,3H)	7.99 (d,3H)			8.09 (s,6H)		

^(a)^ Single; ^(b)^ Double; ^(c)^ Multiple; ^(d)^ Doublet doublet.

**Table 3:** ^13^C-NMR assignment for LDX and its impurities.

Position	LDX	Imp–D	Imp–E	Imp–F	Imp–G	Imp–H	Imp–L	Imp–M
	δC DEPT	δC DEPT	δC DEPT	δC DEPT	δC DEPT	δC DEPT	δC DEPT	δC DEPT
1	167.41–	168.88–	167.87–	174.25–	174.17–	170.61–	167.96–	167.69–
2	52.02 CH	53.35 CH	52.61 CH	54.98 CH	54.97 CH	53.34 CH	52.33 CH	52.47 CH
3	30.38 CH_2_	30.89 CH_2_	30.88 CH_2_	35.00 CH_2_	34.55 CH_2_	28.97 CH_2_	30.78 CH	30.88 CH_2_
4	20.73 CH_2_	21.58 CH_2_	21.57 CH_2_	22.88 CH_2_	22.72 CH_2_	22.96 CH_2_	22.74 CH_2_	21.27 CH_2_
5	26.38 CH_2_	26.72 CH_2_	26.78 CH_2_	29.89 CH_2_	29.83 CH_2_	26.72 CH_2_	26.66 CH_2_	27.01 CH_2_
6	38.53 CH_2_	38.98 CH_2_	38.90 CH_2_	40.25 CH_2_	40.09 CH_2_	32.14 CH_2_	38.95 CH_2_	38.93 CH_2_
7	46.45 CH	46.66 CH	46.84 CH	46.00 CH	45.83 CH	46.54 CH	–	45.31 CH
8	20.83 CH_3_	20.17 CH_3_	21.22 CH_3_	20.54 CH_3_	20.23 CH_3_	21.02 CH_3_	21.46 CH_3_	21.47 CH_3_
9	41.77 CH_2_	41.99 CH_2_	42.17 CH_2_	42.29 CH_2_	42.62 CH_2_	42.19 CH_2_	48.91 CH	36.97 CH_2_
10	138.99 C	139.43 C	139.34 C	139.43 C	138.25 C	139.51 C	144.61 C	125.37 C
11	128.13 CH	128.62 CH	128.50 CH	128.55 CH	128.30 CH	128.46 CH	127.32 CH	131.29 CH
12	129.20 CH	129.61 CH	129.57 CH	129.62 CH	129.38 CH	129.59 CH	128.78 CH	118.94 CH
13	126.18 CH	128.62 CH	126.56 CH	126.45 CH	126.37 CH	126.47 CH	126.37 CH	127.73 CH
14	129.20 CH	129.61 CH	129.57 CH	129.62 CH	129.38 CH	129.55 CH	128.78 CH	115.28 CH
15	128.13 CH	128.62 CH	128.50 CH	128.55 CH	128.30 CH	128.49 CH	127.32 CH	155.85 C
19	39.80 CH_3_	40.07 CH_3_	40.13 CH_3_	156.03–	156.10–	40.16 CH_3_	40.11 CH_3_	–
20		170.61–	168.67–	77.73 C	79.07 C	168.63–		
21		52.47 CH	52.53 CH	28.74 CH_3_	28.42 CH_3_	52.53 CH		
22		31.98 CH_2_	31.07 CH_2_	28.74 CH_3_	28.42 CH_3_	30.90 CH_2_		
23		22.63 CH_2_	21.64 CH_2_	28.74 CH_3_	28.42 CH_3_	21.65 CH_2_		
24,33		26.86 CH_2_	28.83 CH_2_			26.78 CH_2_		
25,34		39.10 CH_2_	38.96 CH_2_			38.97 CH_2_		
28						168.69–		
29						52.25 CH		
31						30.84 CH_2_		
32						21.33 CH_2_		

Note: ^13^C-NMR assignment for Imp-B and Imp-C was identical to LDX.

**Table 4:** Summary of method validation.

Compound	System Suitability				Linearity					Sensitivity	
	RRT ^a^	PC ^b^	SF ^c^	R ^d^	Range (μg/mL)	R ^e^	Slope	Intercept	CF ^f^	LOD ^g^ (μg/mL)	LOQ ^h^ (μg/mL)
LDX	-	19755	1.28	6.09	0.5100–20.4000	0.9999	0.1461	−0.0055	-	0.3060	0.5100
Imp-A	1.17	105353	1.15	66.28	0.5025–20.1000	1.0000	0.2271	−0.0276	0.61	0.3028	0.5025
Imp-B	1.12	121354	1.02	3.50	0.5110–20.4400	1.0000	0.1262	−0.0141	1.10	0.3010	0.5105
Imp-C	1.12	125659	1.03	3.50	0.5047–20.1900	1.0000	0.1119	−0.0010	1.31	0.3026	0.5105
−Imp-D	0.93	98972	1.09	3.33	0.5160–20.6400	1.0000	0.1112	−0.0079	1.04	0.3035	0.5070
Imp-E	0.89	118365	1.14	3.19	0.5135–20.5400	1.0000	0.0836	−0.0013	1.66	0.3041	0.5051
Imp-F	2.38	749255	1.63	12.44	0.5022–20.0900	1.0000	0.1700	−0.0061	0.86	0.3013	0.5023
Imp-G	2.25	864920	1.16	80.95	0.5070–20.2800	1.0000	0.1641	−0.0119	0.89	0.3042	0.5070
Imp-H	0.725	46840	1.28	8.53	0.5028–20.1900	1.0000	0.1524	−0.0085	0.92	0.3028	0.5062
Imp-J	3.08	28465	1.14	-	0.5080–20.3200	0.9998	0.1269	0.0110	1.15	0.3028	0.5041
Imp-K	2.15	322224	1.12	7.00	0.5105–20.256	0.9999	0.1389	0.0105	1.32	0.3036	0.5075
Imp-L	0.83	90222	1.02	8.53	0.5180–20.7200	1.0000	0.1159	−0.0056	1.19	0.3041	0.5180
Imp-M	0.86	114119	1.17	3.40	0.5240–20.9592	1.0000	0.1614	−0.0011	0.91	0.3060	0.5140

^a^ Relative retention time; ^b^ (USP) plate count; ^c^ Symmetry factor; ^d^ (USP) resolution; ^e^ Correlation factor. ^f^ Calibration response factor. ^g^ (S/N ≥ 3). ^h^ (S/N ≥10).

**Table 5:** Summary of accuracy.

Impurity	0.05%–1%	0.05%–2%	0.05%–3%	0.10%–1%	0.10%–2%	0.10%–3%	0.15%–1%	0.15%–2%	0.15%–3%	Mean	RSD (n = 9)
Imp-A	91.8	90.2	92.6	89.6	92.7	94.1	92.0	92.5	94.1	92.0	1.83
Imp-B	102.6	105.1	100.2	100.9	91.3	97.3	92.6	90.8	90.5	96.8	5.82
Imp-C	92.8	90.4	91.2	100.6	98.7	99.5	97.4	102.4	98.5	96.8	4.44
Imp-D	99.7	96.3	95.8	97.7	96.3	97.6	94.1	97.0	93.1	96.4	2.04
Imp-E	99.1	101.0	102.7	101.6	96.5	95.7	103.5	103.4	102.6	100.6	2.91
Imp-F	103.9	96.6	97.1	103.3	105.6	99.3	98.8	100.3	98.1	100.3	3.19
Imp-G	101.5	102.4	100.3	103.3	96.6	102.6	95.8	100.9	100.5	100.4	2.59
Imp-H	99.5	98.7	102.5	101.8	98.9	103.5	96.9	99.5	102.1	100.3	2.16
Imp-J	92.7	95.4	93.9	95.0	96.2	93.5	91.0	99.4	94.0	94.5	2.50
Imp-K	99.7	103.5	101.6	98.7	99.5	96.4	105.1	103.6	104.2	101.3	2.90
Imp-L	91.4	90.6	101.2	95.7	95.5	94.8	96.7	90.0	94.9	94.5	3.69
Imp-M	97.8	98.5	93.2	95.1	94.4	97.6	91.3	95.5	92.7	95.1	2.62

**Table 6:** Summary of precision.

Compound	1	2	3	4	5	6	RSD (n = 6)
	C (mg/mL)						
	1.0080	1.0225	1.0180	1.0290	1.0130	1.0095	
Imp-A	0.10	0.10	0.09	0.10	0.10	0.10	4.22
Imp-B	0.10	0.11	0.11	0.11	0.11	0.11	2.19
Imp-C	0.12	0.12	0.12	0.12	0.12	0.13	3.24
Imp-D	0.09	0.09	0.09	0.09	0.09	0.08	1.67
Imp-E	0.10	0.10	0.10	0.11	0.10	0.10	1.83
Imp-F	0.12	0.12	0.12	0.12	0.11	0.11	1.20
Imp-G	0.10	0.10	0.10	0.10	0.10	0.10	1.30
Imp-H	0.10	0.11	0.11	0.10	0.10	0.10	1.51
Imp-J	0.11	0.11	0.10	0.10	0.10	0.10	2.14
Imp-K	0.11	0.11	0.11	0.10	0.10	0.11	1.63
Imp-L	0.11	0.11	0.11	0.11	0.11	0.11	1.32
Imp-M	0.12	0.11	0.11	0.11	0.11	0.11	1.87
RRT = 1.32	0.01	0.01	0.01	0.02	0.02	0.01	2.85

**Table 7:** Summary of robustness.

Compound	Column Temperature			Flow Rate			UV		
	27 °C	30 °C	33 °C	0.9 mL/min	1.0 mL/min	1.1 mL/min	213 nm	215 nm	217 nm
Imp-A	0.10	0.10	0.09	0.10	0.10	0.09	0.11	0.10	0.10
Imp-B	0.13	0.12	0.13	0.12	0.13	0.12	0.12	0.13	0.13
Imp-C	0.10	0.11	0.10	0.13	0.12	0.13	0.11	0.10	0.12
Imp-D	0.09	0.09	0.08	0.09	0.09	0.10	0.09	0.09	0.08
Imp-E	0.11	0.10	0.11	0.11	0.10	0.10	0.10	0.10	0.10
Imp-F	0.10	0.09	0.09	0.09	0.10	0.10	0.09	0.10	0.09
Imp-G	0.09	0.09	0.09	0.10	0.09	0.10	0.09	0.09	0.10
Imp-H	0.09	0.09	0.08	0.09	0.09	0.08	0.09	0.09	0.09
Imp-J	0.08	0.09	0.08	0.07	0.08	0.07	0.07	0.07	0.07
Imp-K	0.10	0.10	0.11	0.10	0.10	0.11	0.11	0.10	0.10
Imp-L	0.11	0.12	0.12	0.11	0.11	0.12	0.12	0.12	0.12
Imp-M	0.08	0.08	0.09	0.08	0.09	0.08	0.09	0.09	0.09

## Supplementary Materials

The following are available online, Table of contents; Figure S5 the LC-MS spectrum of LDX and its impurities; Figure S6 The spectrum of Imp-B and Imp-C in normal-phase chromatography; Figure S7 NMR, HRMS and IR spectrogram of LDX and its impurities.
