# Supplementary material for: Identification, Characterization and Quantification of Process-Related and Degradation Impurities in Lisdexamfetamine Dimesylate: Identifiction of Two New Compounds
Source: Molecules. 2018 Nov 29;23(12):3125. doi: 10.3390/molecules23123125 (PMC6321639; doi:10.3390/molecules23123125)
Supplement: Supplementary file 1 [file molecules-23-03125-s001.pdf]

Identification, characterization and quantification of process-related and degradation impurities in Lisdexamfetamine dimesylate: Two identified as new compounds

Shenghua Gao[1], Lili Meng[1], Chunjie Zhao[2], Tao Zhang[1], Pengcheng Qiu [1,*] and Fuli Zhang[1,*]

* Correspondence: zhangfuli1@sinopharm.com(F.Z.); pcqiu@aliyun.com (P.Q.);

Tel.:+86-21-2057-2000(ext.5036) (F.Z.); +86-21-2057-2000(ext.5077) (P.Q.)

Supporting Information

Table of contents

Figure S5 The LC-MS spectrum of LDX and its impurities P2-P7

Figure S6 The spectrum of Imp-B and Imp-C in normal-phase chromatography P7

Figure S7 NMR, HRMS and IR spectrogram of LDX and its impurities P8-P47

Molecules 2018, *23*, 3125; doi:10.3390/molecules23123125

Figure S5 The LC-MS spectrum of LDX and its impurities

(a) The LC chromatogram of LDX and its impurities;

(b) The MS spectrum of Imp-L;

(c) The MS spectrum of Imp-M;

(d) The MS spectrum of Imp-A;

(e) The MS spectrum of Imp-H;

(f) The MS spectrum of LDX;

(g) The MS spectrum of Imp-E;

(h) The MS spectrum of Imp-D;

(i) The MS spectrum of Imp-B or C;

(j) The MS spectrum of Imp-K;

(k) The MS spectrum of Imp-G;

(l) The MS spectrum of Imp-F;

(m) The MS spectrum of Imp-J.

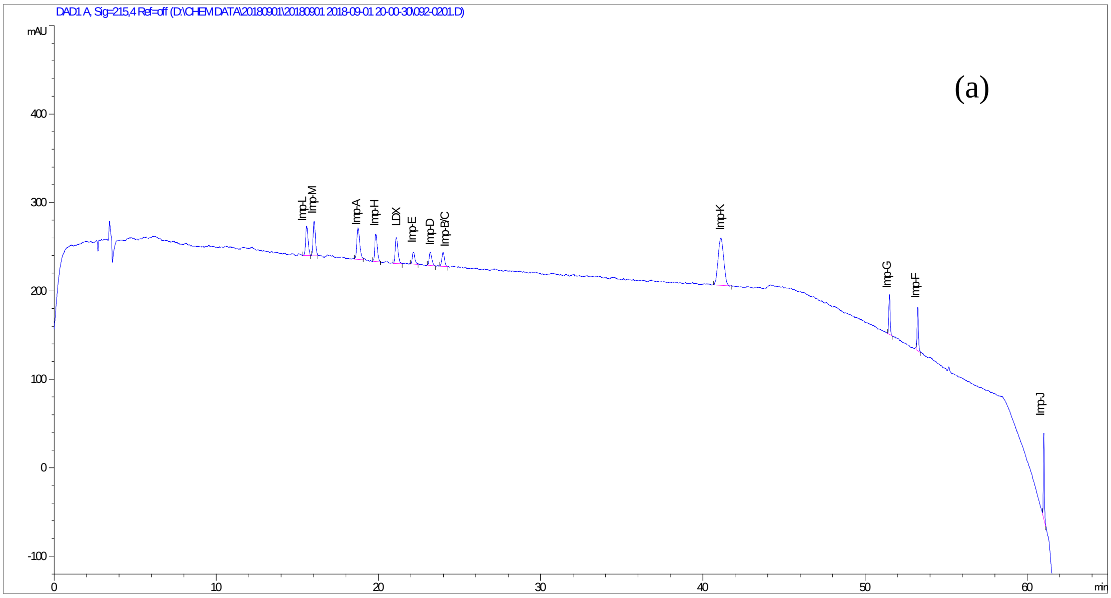

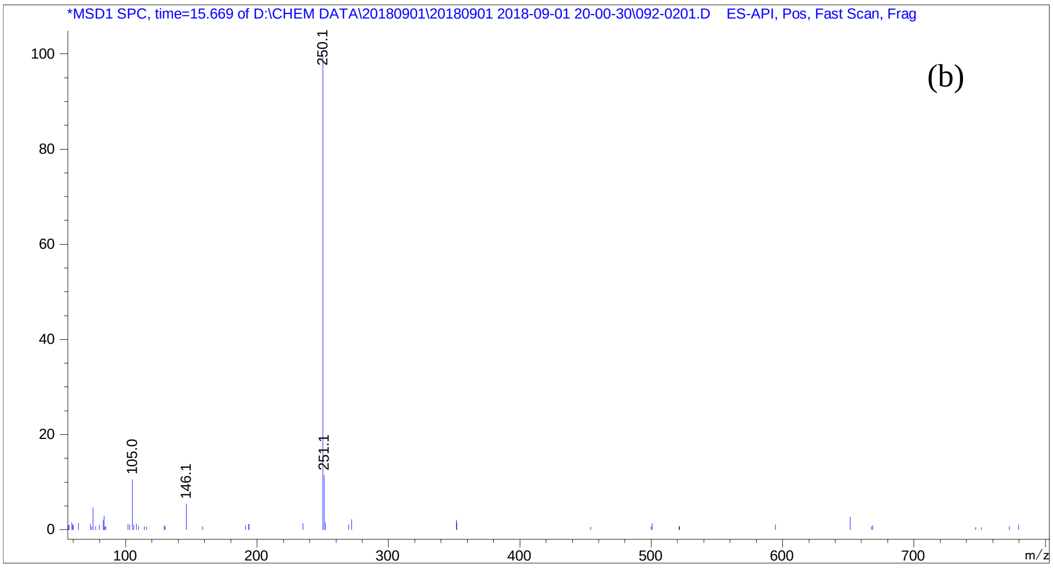
*MSD1 SPC, time=15.669 of D:\CHEM DATA\20180901\20180901 2018-09-01 20-00-30\092-0201.D ES-API, Pos, Fast Scan, Frag
(b)
250.1
251.1
105.0
146.1
0
20
40
60
80
100
100
200
300
400
500
600
700
m/z

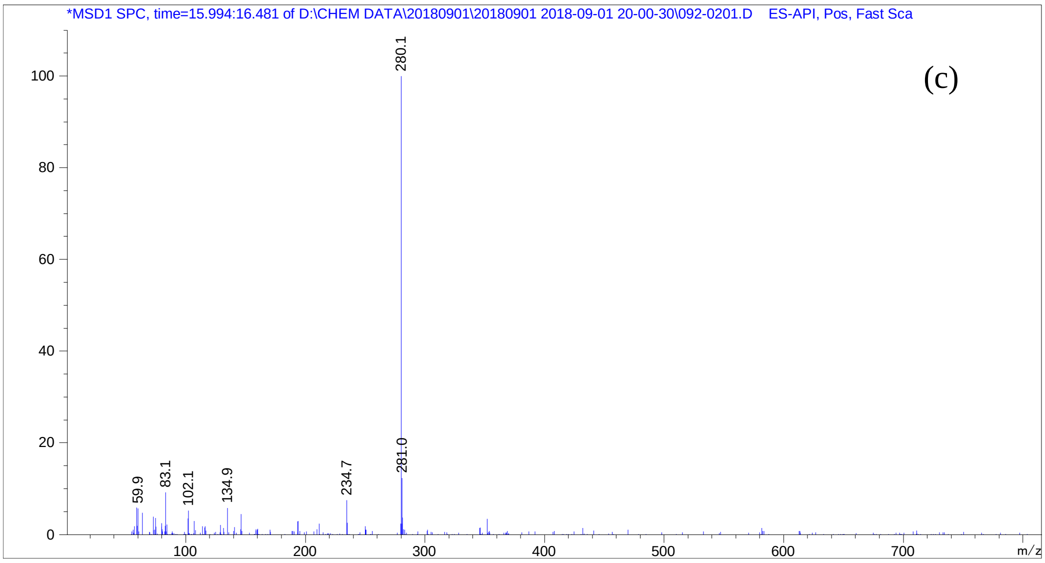
*MSD1 SPC, time=15.994:16.481 of D:\CHEM DATA\20180901\20180901 2018-09-01 20-00-30\092-0201.D ES-API, Pos, Fast Sca
(c)
280.1
281.0
59.9
83.1
102.1
134.9
234.7
0
20
40
60
80
100
100
200
300
400
500
600
700
m/z

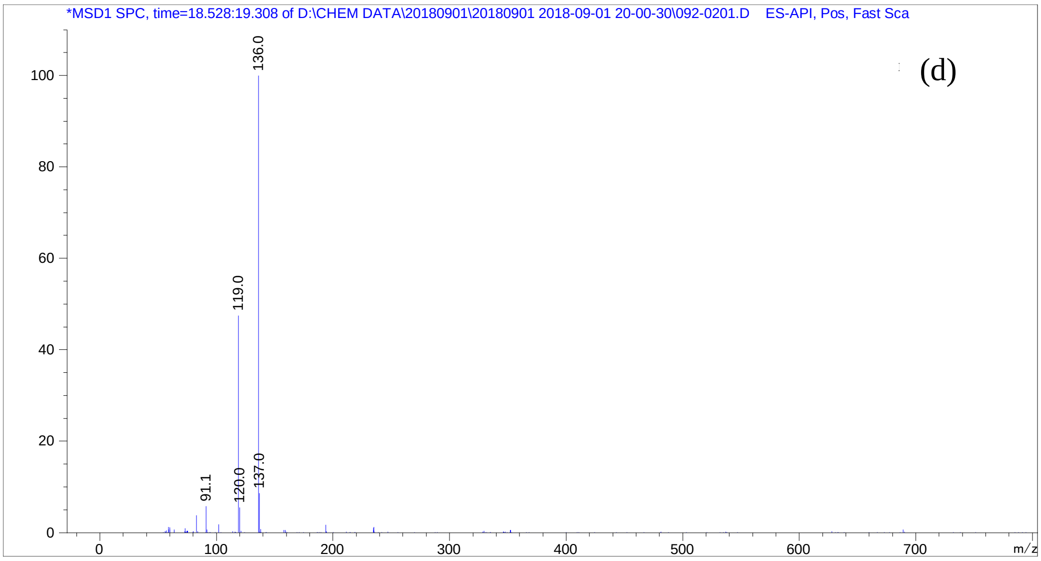
*MSD1 SPC, time=18.528:19.308 of D:\CHEM DATA\20180901\20180901 2018-09-01 20-00-30\092-0201.D ES-API, Pos, Fast Sca
(d)
136.0
119.0
91.1
120.0
137.0
0
20
40
60
80
100
0
100
200
300
400
500
600
700
m/z

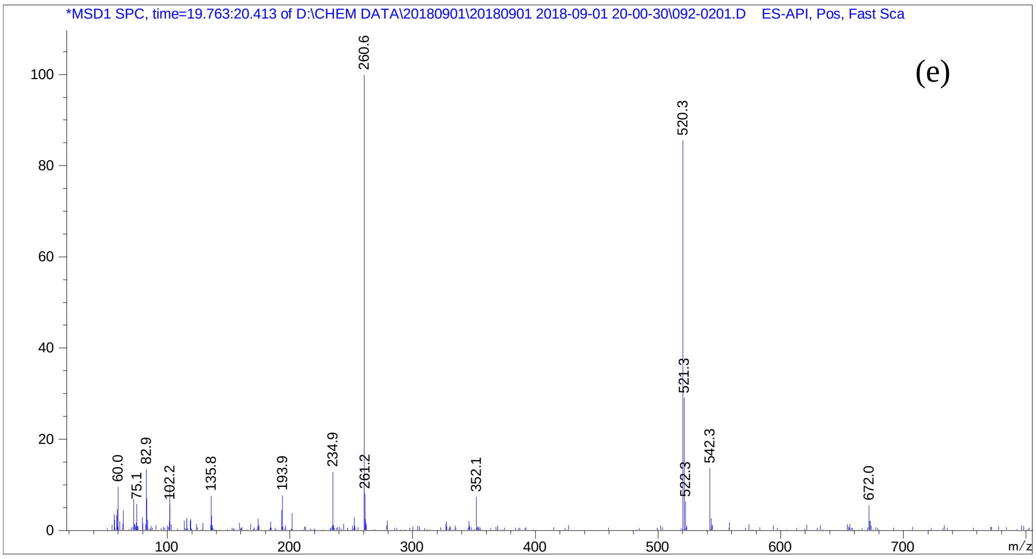
*MSD1 SPC, time=19.763:20.413 of D:\CHEM DATA\20180901\20180901 2018-09-01 20-00-30\092-0201.D ES-API, Pos, Fast Sca
(e)
260.6
520.3
521.3
542.3
672.0
352.1
261.2
234.9
193.9
135.8
102.2
82.9
75.1
60.0
522.3
m/z

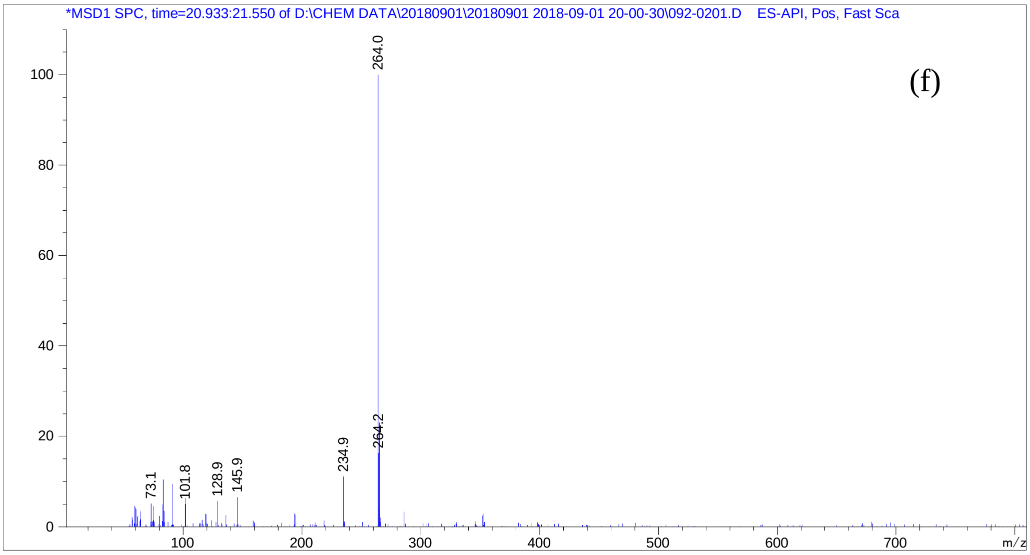
*MSD1 SPC, time=20.933:21.550 of D:\CHEM DATA\20180901\20180901 2018-09-01 20-00-30\092-0201.D ES-API, Pos, Fast Sca
(f)
264.0
264.2
234.9
145.9
128.9
101.8
73.1
m/z

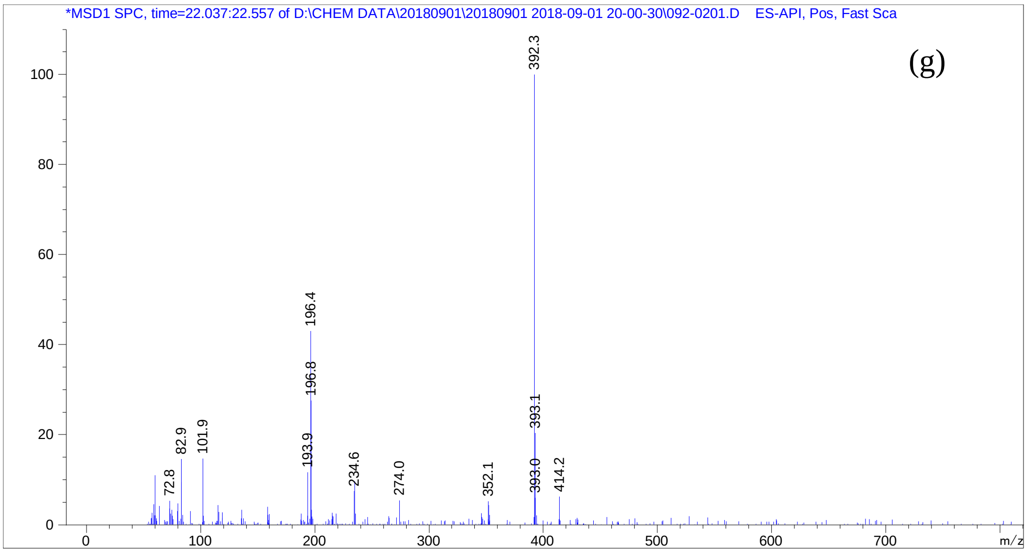
*MSD1 SPC, time=22.037:22.557 of D:\CHEM DATA\20180901\20180901 2018-09-01 20-00-30\092-0201.D ES-API, Pos, Fast Sca
(g)
392.3
196.4
196.8
393.1
101.9
82.9
193.9
72.8
234.6
274.0
352.1
393.0
414.2
m/z

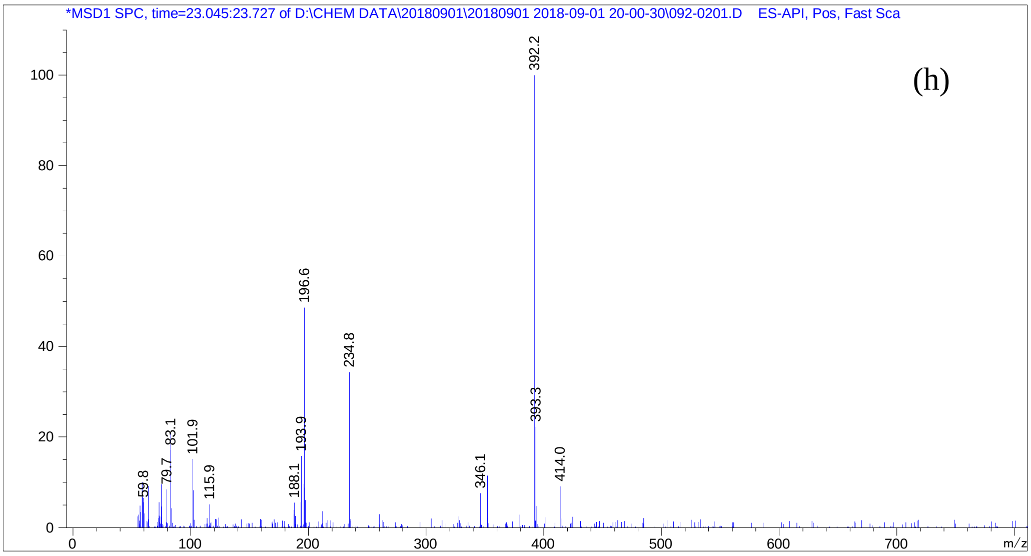
*MSD1 SPC, time=23.045:23.727 of D:\CHEM DATA\20180901\20180901 2018-09-01 20-00-30\092-0201.D ES-API, Pos, Fast Sca
(h)
392.2
196.6
234.8
393.3
83.1
101.9
193.9
414.0
346.1
59.8
79.7
115.9
188.1
m/z

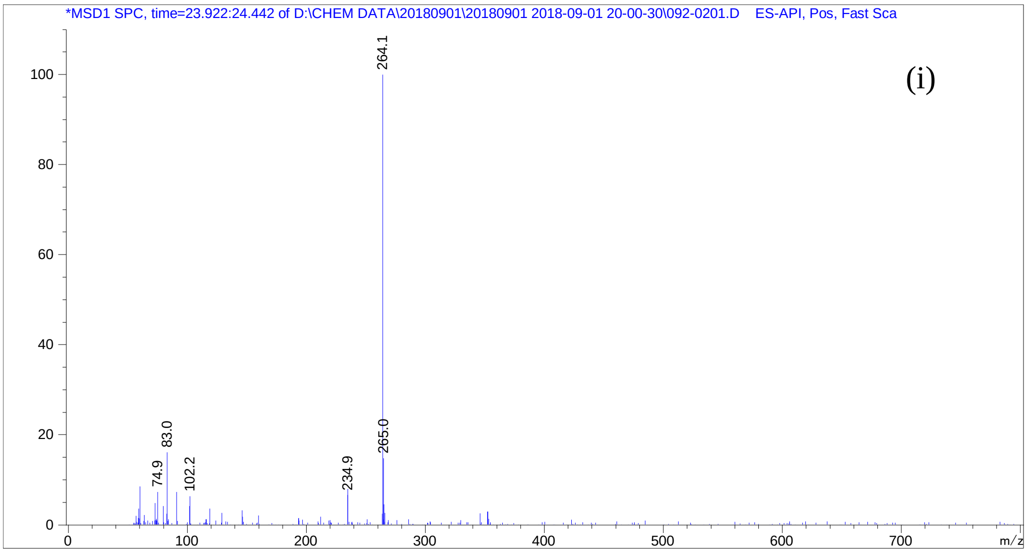
*MSD1 SPC, time=23.922:24.442 of D:\CHEM DATA\20180901\20180901 2018-09-01 20-00-30\092-0201.D ES-API, Pos, Fast Sca
(i)
264.1
83.0
265.0
74.9
102.2
234.9
m/z

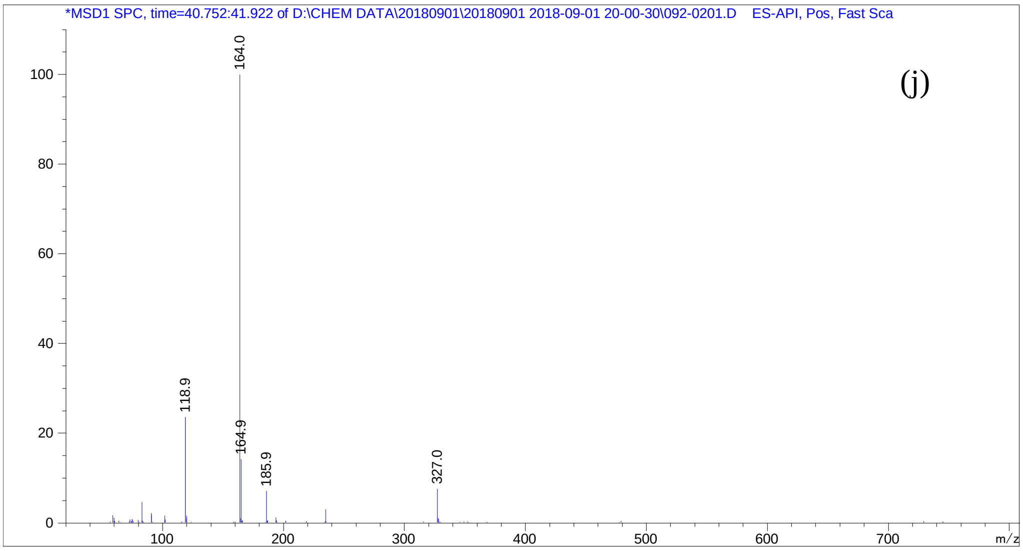
*MSD1 SPC, time=40.752:41.922 of D:\CHEM DATA\20180901\20180901 2018-09-01 20-00-30\092-0201.D ES-API, Pos, Fast Sca
(j)
164.0
118.9
164.9
185.9
327.0
m/z

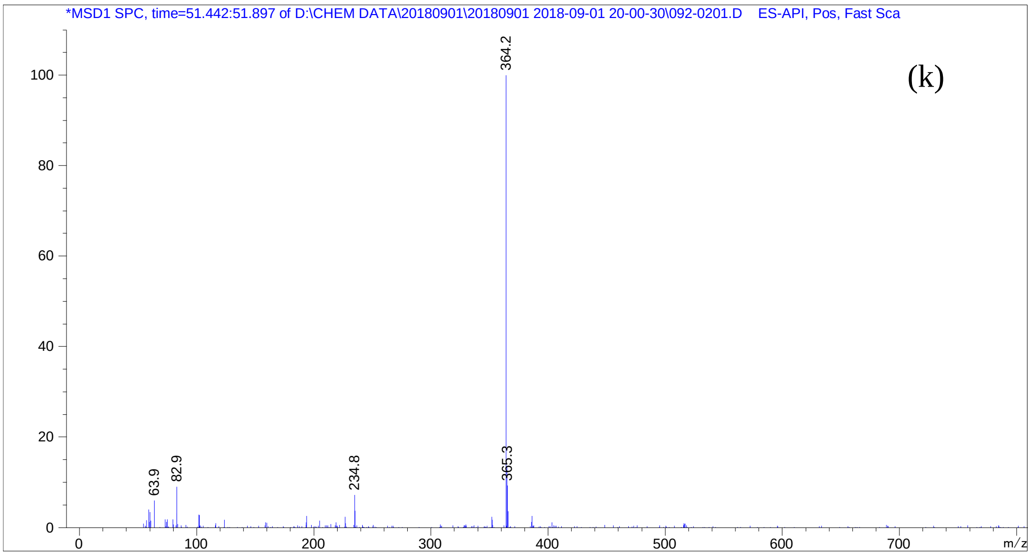
*MSD1 SPC, time=51.442:51.897 of D:\CHEM DATA\20180901\20180901 2018-09-01 20-00-30\092-0201.D ES-API, Pos, Fast Sca
(k)
364.2
365.3
234.8
82.9
63.9
m/z

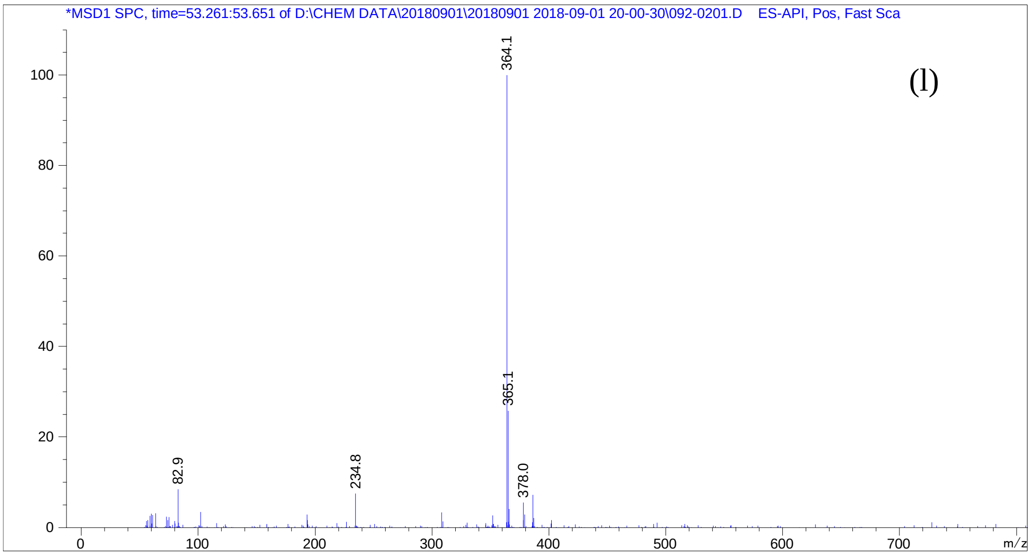
*MSD1 SPC, time=53.261:53.651 of D:\CHEM DATA\20180901\20180901 2018-09-01 20-00-30\092-0201.D ES-API, Pos, Fast Sca
(l)
364.1
365.1
378.0
234.8
82.9
m/z

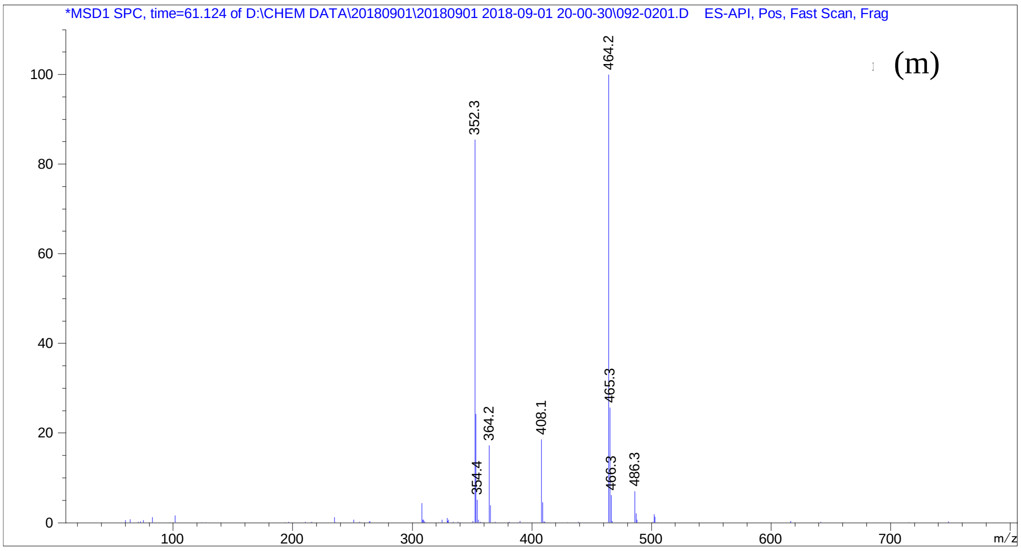
*MSD1 SPC, time=61.124 of D:\CHEM DATA\20180901\20180901 2018-09-01 20-00-30\092-0201.D ES-API, Pos, Fast Scan, Frag
(m)
464.2
352.3
465.3
408.1
364.2
486.3
466.3
354.4
m/z

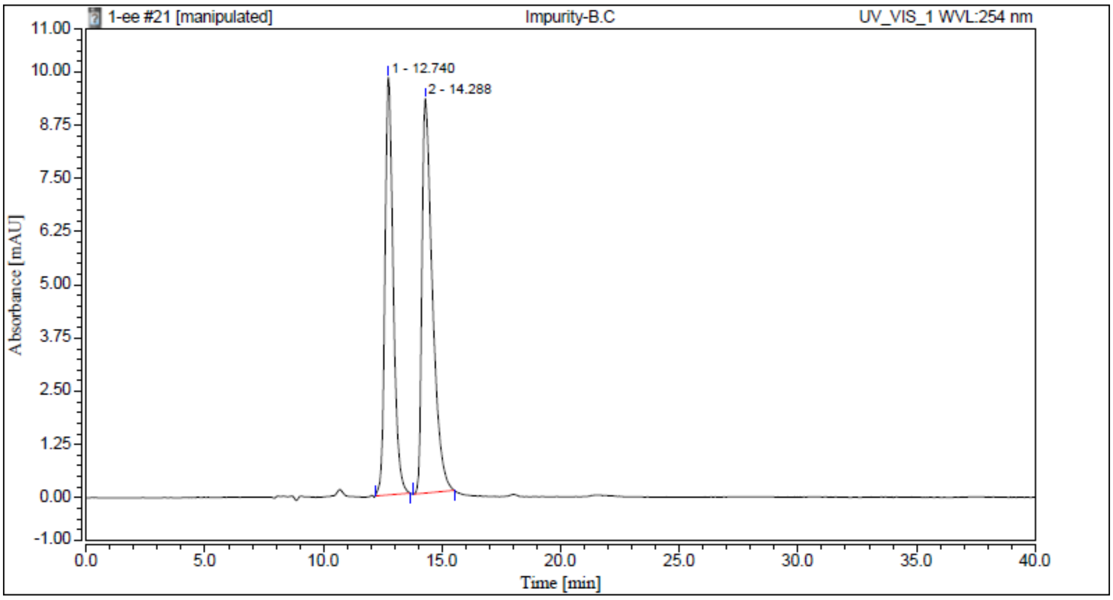

Figure S6 The spectrum of Imp-B and Imp-C in normal-phase chromatography

FigureS7 The spectrums of LDX and its impurities

(X-1) the ^{1}H NMR spectrum of LDX;

(X-2) the ^{13}C NMR spectrum of LDX;

(X-3) the DEPT spectrum of LDX;

(X-4) the IR spectrum of LDX;

(X-5) the HRMS spectrum of LDX;

(A-1) the ^{1}H NMR spectrum of Imp-A;

(A-2) the ^{13}C NMR spectrum of Imp-A;

(A-3) the DEPT spectrum of Imp-A;

(A-4) the IR spectrum of Imp-A;

(A-5) the HRMS spectrum of Imp-A;

(B-1) the ^{1}H NMR spectrum of Imp-B;

(C-1) the ^{1}H NMR spectrum of Imp-C;

(C-2) the ^{13}C NMR spectrum of Imp-C;

(C-3) the DEPT spectrum of Imp-C;

(C-4) the HRMS spectrum of Imp-C;

(D-1) the ^{1}H NMR spectrum of Imp-D;

(D-2) the ^{13}C NMR spectrum of Imp-D;

(D-3) the DEPT spectrum of Imp-D;

(D-4) the HSQC spectrum of Imp-D;

(D-5) the HMBC spectrum of Imp-D;

(D-6) the enlarged view of the HMBC spectrum of Imp-D;

(D-7) the IR spectrum of Imp-D;

(D-8) the HRMS spectrum of Imp-D;

(E-1) the ^{1}H NMR spectrum of Imp-E;

(E-2) the ^{13}C NMR spectrum of Imp-E;

(E-3) the DEPT spectrum of Imp-E;

(E-4) the HSQC spectrum of Imp-E;

(E-5) the HMBC spectrum of Imp-E;

(E-6) the enlarged view of the HMBC spectrum of Imp-E;

(E-7) the IR spectrum of Imp-E;

(E-8) the HRMS spectrum of Imp-E;

(F-1) the ^{1}H NMR spectrum of Imp-F;

(F-2) the ^{13}C NMR spectrum of Imp-F;

(F-3) the DEPT spectrum of Imp-F;

(F-4) the IR spectrum of Imp-F;

(F-5) the HRMS spectrum of Imp-F;

(G-1) the ^{1}H NMR spectrum of Imp-G;

(G-2) the ^{13}C NMR spectrum of Imp-G;

(G-3) the DEPT spectrum of Imp-G;

(G-4) the IR spectrum of Imp-G;

(G-5) the HRMS spectrum of Imp-G;

(H-1) the ^{1}H NMR spectrum of Imp-H;

(H-2) the ^{13}C NMR spectrum of Imp-H;

(H-3) the DEPT spectrum of Imp-H;

(H-4) the HMBC spectrum of Imp-H;

(H-5) the enlarged view of the HMBC spectrum of Imp-H;

(H-6) the HSQC spectrum of Imp-H;

(H-7) the COSY spectrum of Imp-H;

(H-8) the enlarged view of the COSY spectrum of Imp-H;

(H-9) the IR spectrum of Imp-H;

(H-10) the HRMS spectrum of Imp-H;

(J-1) the ^{1}H NMR spectrum of Imp-J;

(J-2) the ^{13}C NMR spectrum of Imp-J;

(J-3) the DEPT spectrum of Imp-J;

(J-4) the HRMS spectrum of Imp-J;

(K-1) the ^{1}H NMR spectrum of Imp-K;

(K-2) the ^{13}C NMR spectrum of Imp-K;

(K-3) the HRMS spectrum of Imp-K;

(L-1) the ^{1}H NMR spectrum of Imp-L;

(L-2) the ^{13}C NMR spectrum of Imp-L;

(L-3) the DEPT spectrum of Imp-L;

(L-4) the IR spectrum of Imp-L;

(L-5) the HRMS spectrum of Imp-L;

(M-1) the ^{1}H NMR spectrum of Imp-M;

(M-2) the ^{13}C NMR spectrum of Imp-M;

(M-3) the DEPT spectrum of Imp-M;

(M-4) the COSY spectrum of Imp-M;

(M-5) the HSQC spectrum of Imp-M;

(M-6) the HMBC spectrum of Imp-M;

(M-7) the enlarged view of the HMBC spectrum of Imp-M;

(M-8) the enlarged view of the HMBC spectrum of Imp-M;

(M-9) the HRMS spectrum of Imp-M;

(N-1) the ^{1}H NMR spectrum of B-3;

(N-2) the ^{1}H NMR spectrum of B-2;

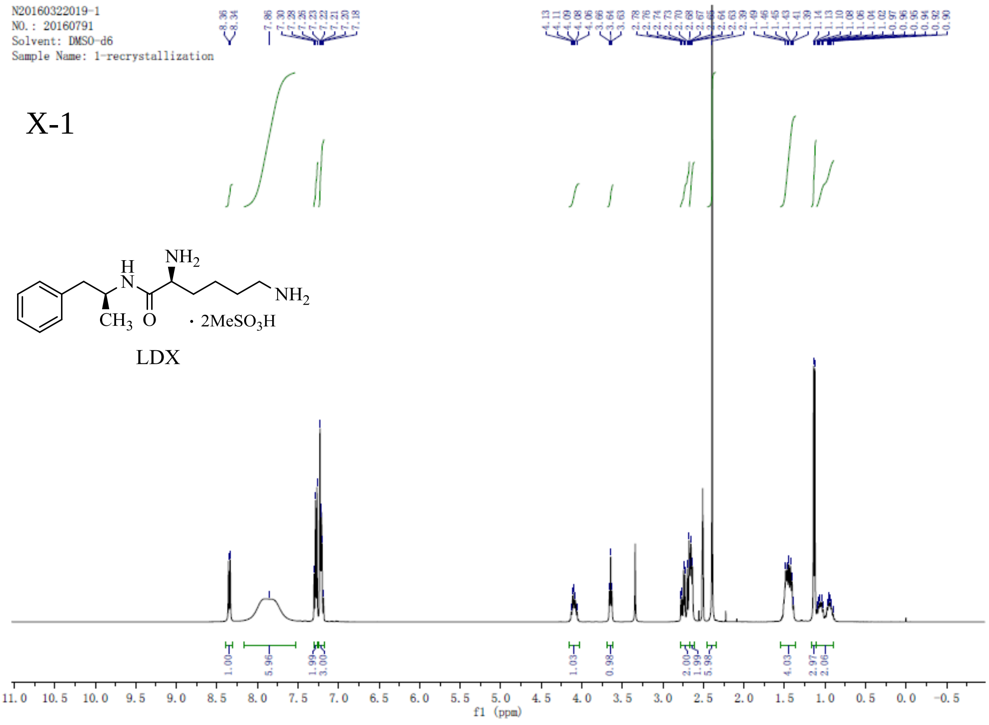

LDX ^{1}H NMR DMSO 400 MHz

X-2

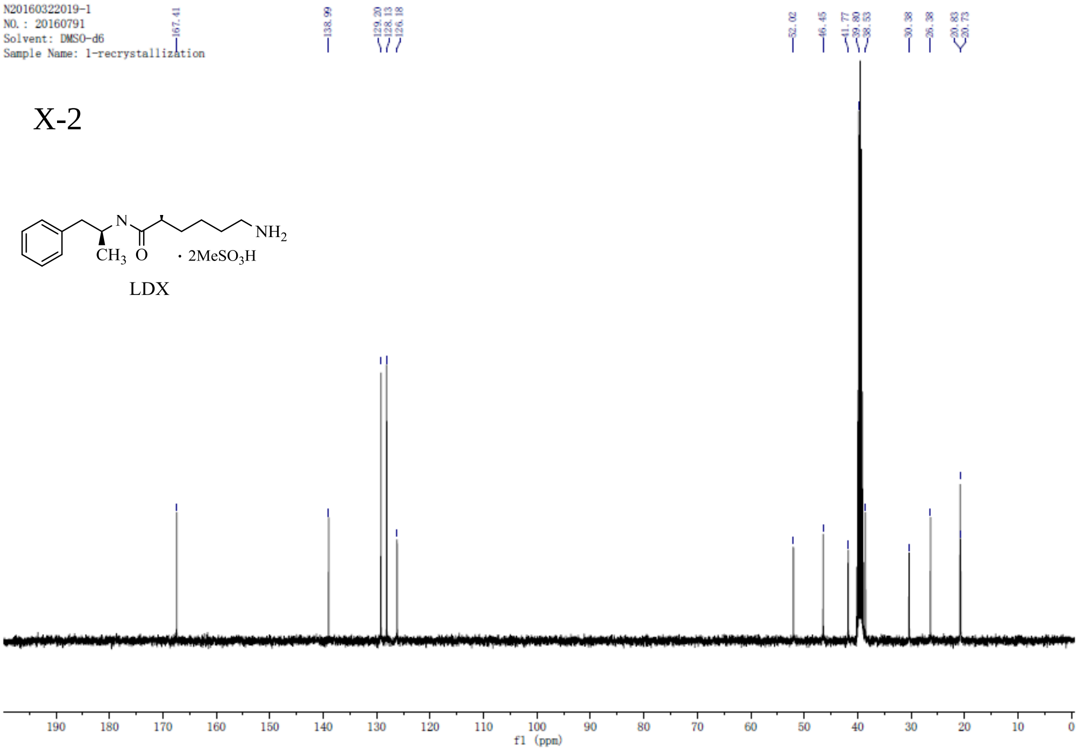

LDX ^{13}C NMR DMSO 101 MHz

X-3

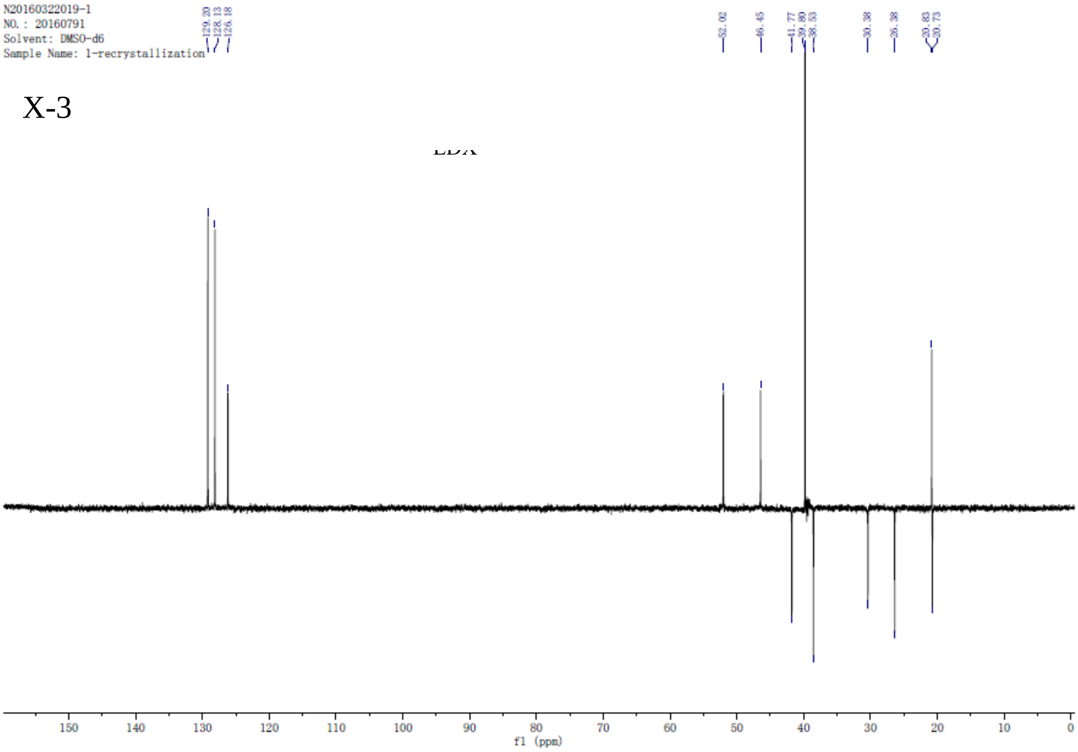

LDX DEPT DMSO 101 MHz

X-4

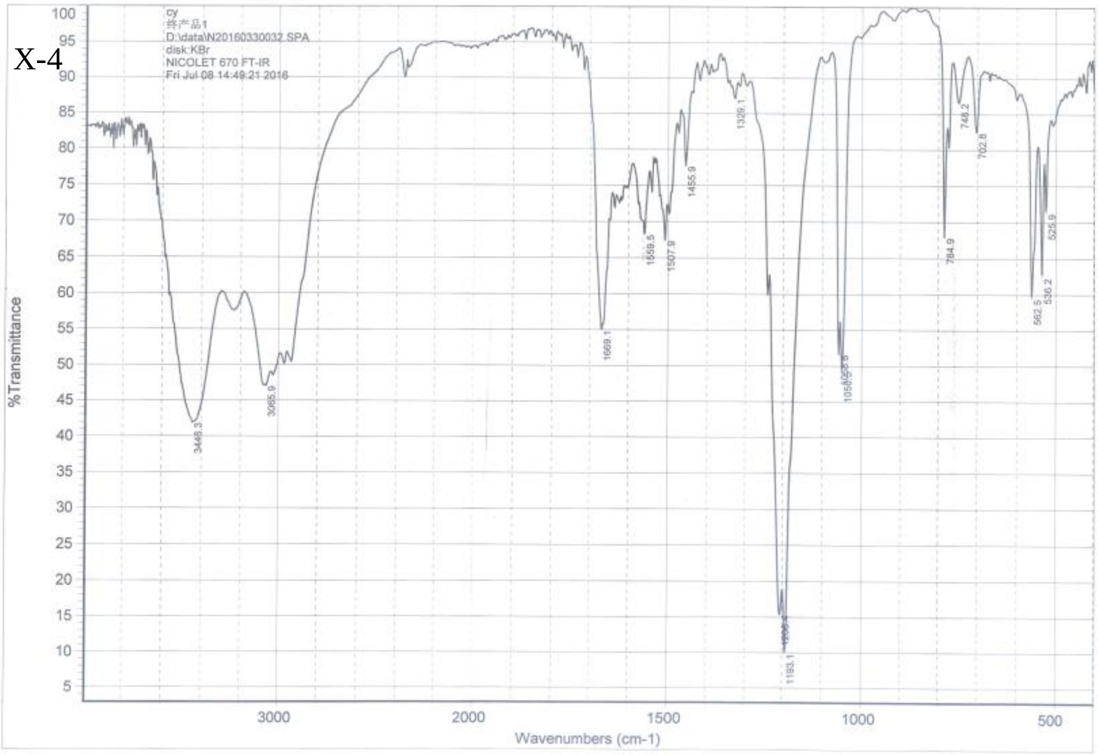

LDX IR

X-5

Qualitative Compound Report

**Data File**	20160630-017.d	**Sample Name**	LYBBA-1
**Sample Type**	Sample	**Position**	Vial 50
**Instrument Name**	Instrument 1	**User Name**	
**Acq Method**		**IRM Calibration Status**	Success
**DA Method**	MS.m	**Comment**	N20160530015

Compound Table

Compound Label	RT	Mass	Abund	Formula	Tgt Mass	Diff (ppm)
Cpd 1: C15 H25 N3 O	-0.24	263.2004	1178593	C15 H25 N3 O	263.1998	2.3

Compound Label	RT	Algorithm	Mass
Cpd 1: C15 H25 N3 O	-0.24	Find By Formula	263.2004

MS Zoomed Spectrum

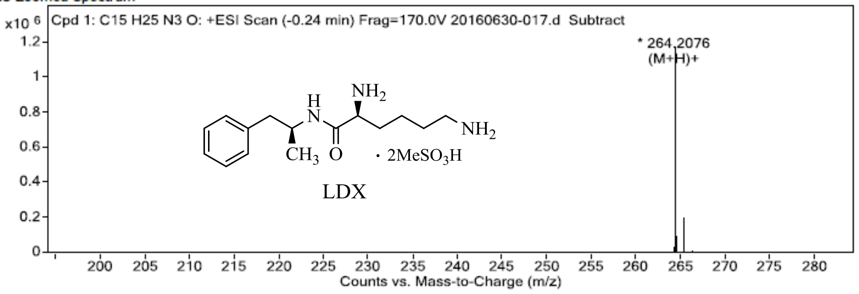

MS Spectrum Peak List

m/z	*Calc m/z*	Diff(ppm)	Abund	Formula	Ion
264.2076	264.207	2.3	1178593	C15 H26 N3 O	(M+H)+

A-1

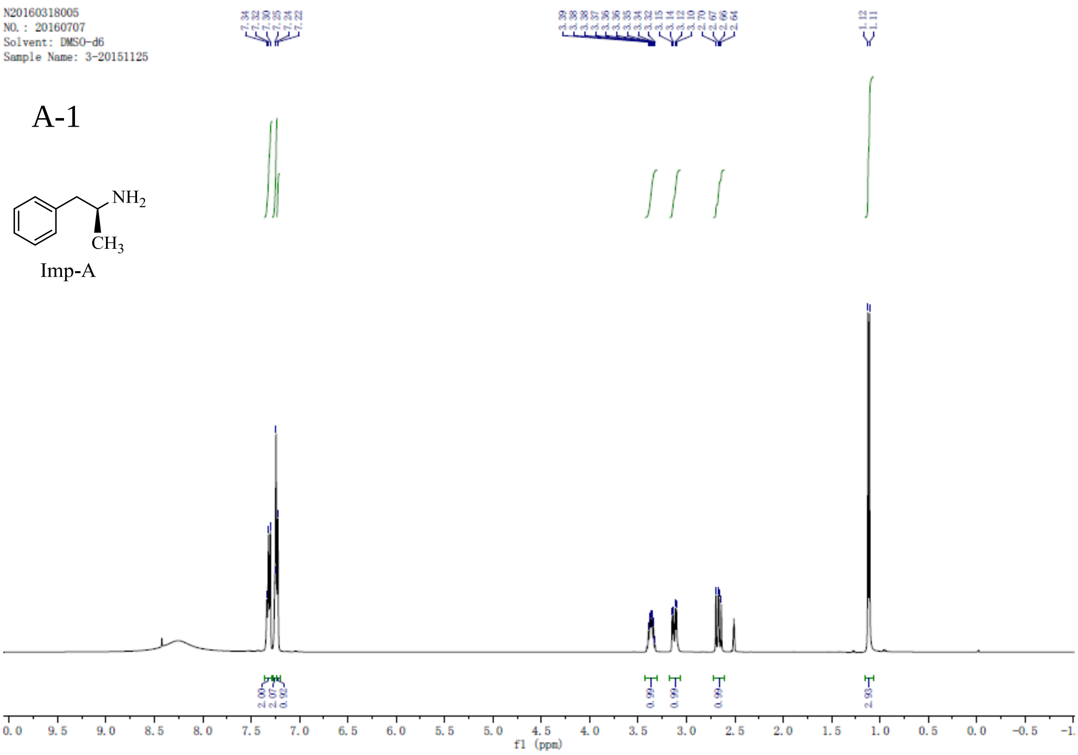

Imp-A ^{1}H NMR DMSO 400 MHz

A-2

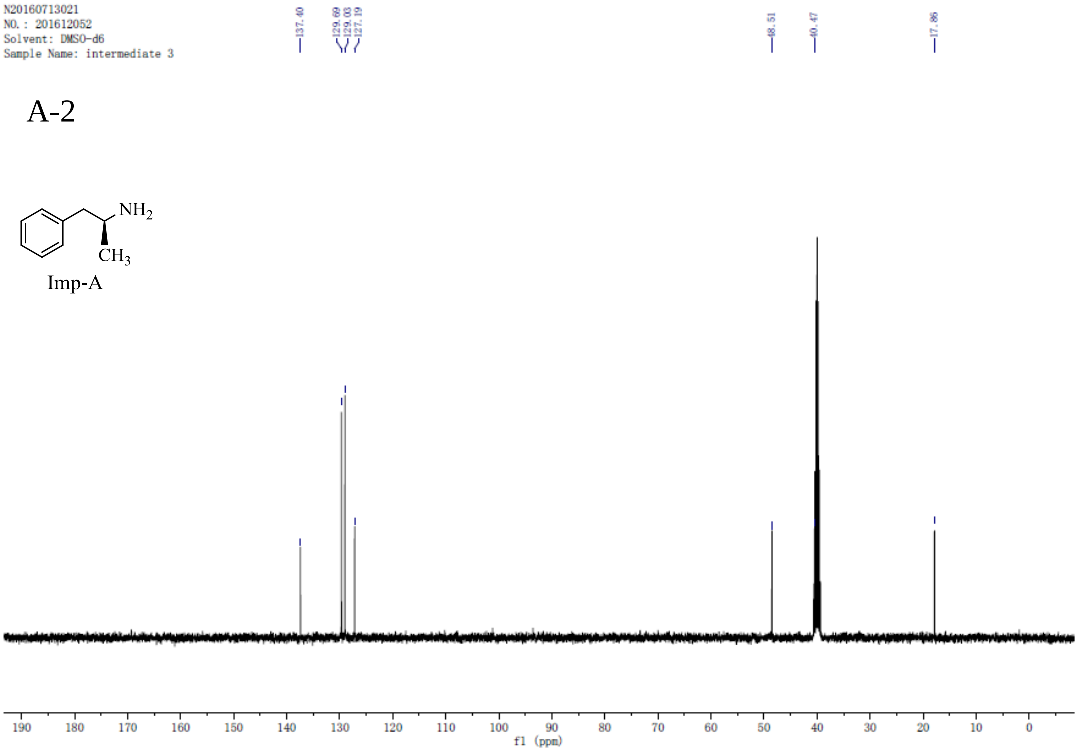

Imp-A ^{13}C NMR DMSO 101 MHz

A-3

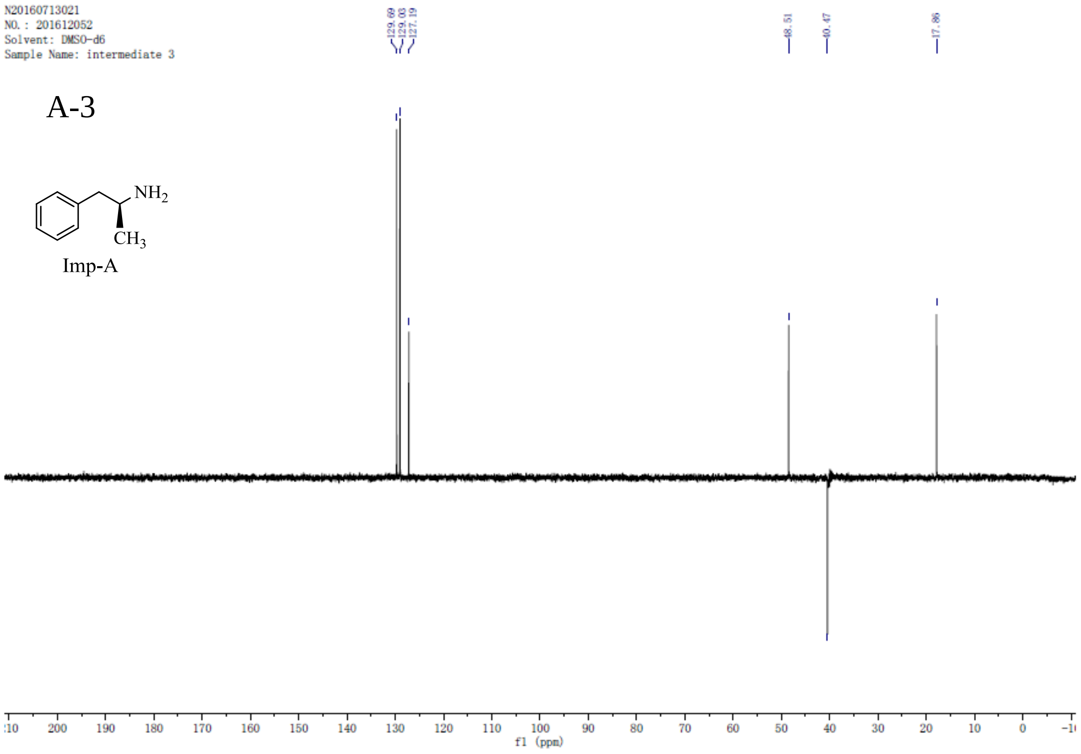

Imp-A DEPT DMSO 101 MHz

A-4

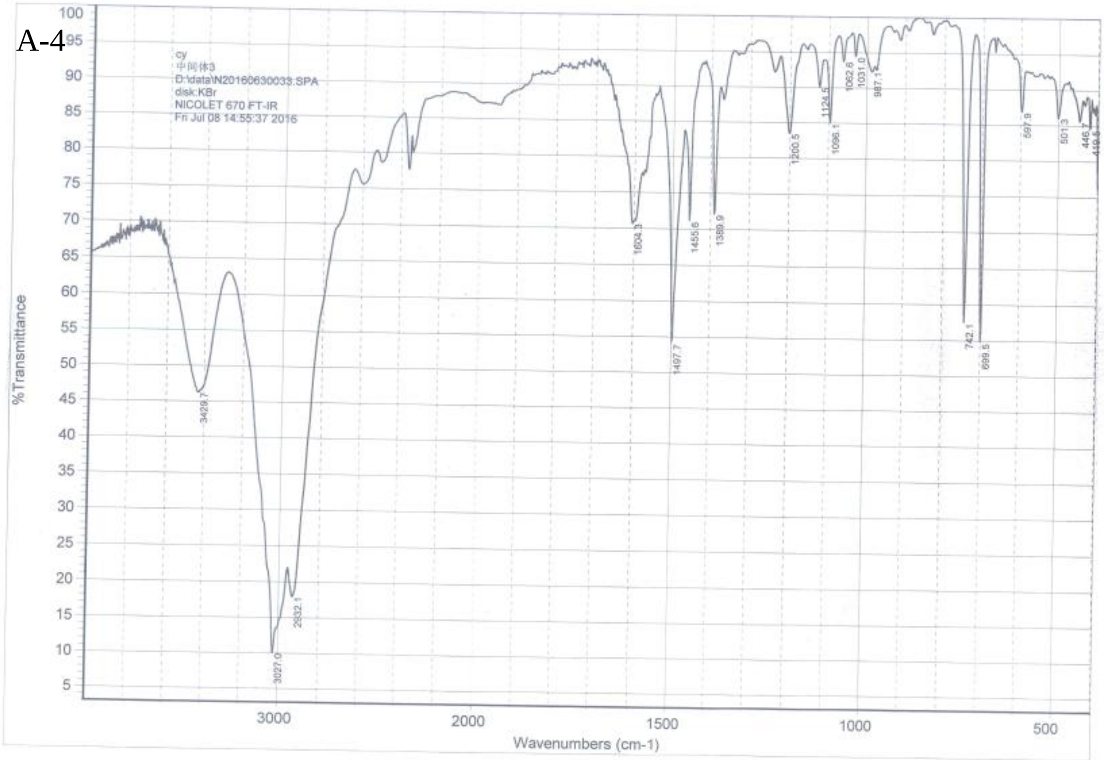

Imp-A IR

A-5

Qualitative Compound Report

**Data File**	20160706-001.d	**Sample Name**	Int-3
**Sample Type**	Sample	**Position**	Vial 76
**Instrument Name**	Instrument 1	**User Name**	
**Acq Method**		**IRM Calibration Status**	Success
**DA Method**	MS.m	**Comment**	N20160706003

Compound Table

Compound Label	RT	Mass	Abund	Formula	Tgt Mass	Diff (ppm)
Cpd 1: C9 H13 N	0.17	135.1048	264278	C9 H13 N	135.1048	-0.11

Compound Label	RT	Algorithm	Mass
Cpd 1: C9 H13 N	0.17	Find By Formula	135.1048

MS Zoomed Spectrum

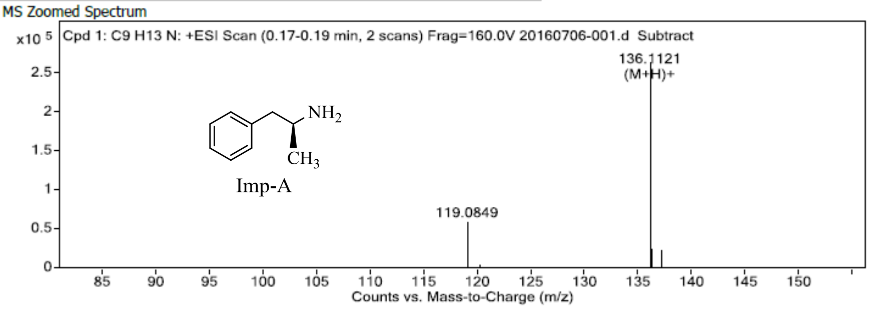

MS Spectrum Peak List

m/z	*Calc m/z*	Diff(ppm)	z	Abund	Formula	Ion
136.1121	136.1121	-0.11	1	264278	C9 H14 N	(M+H)+

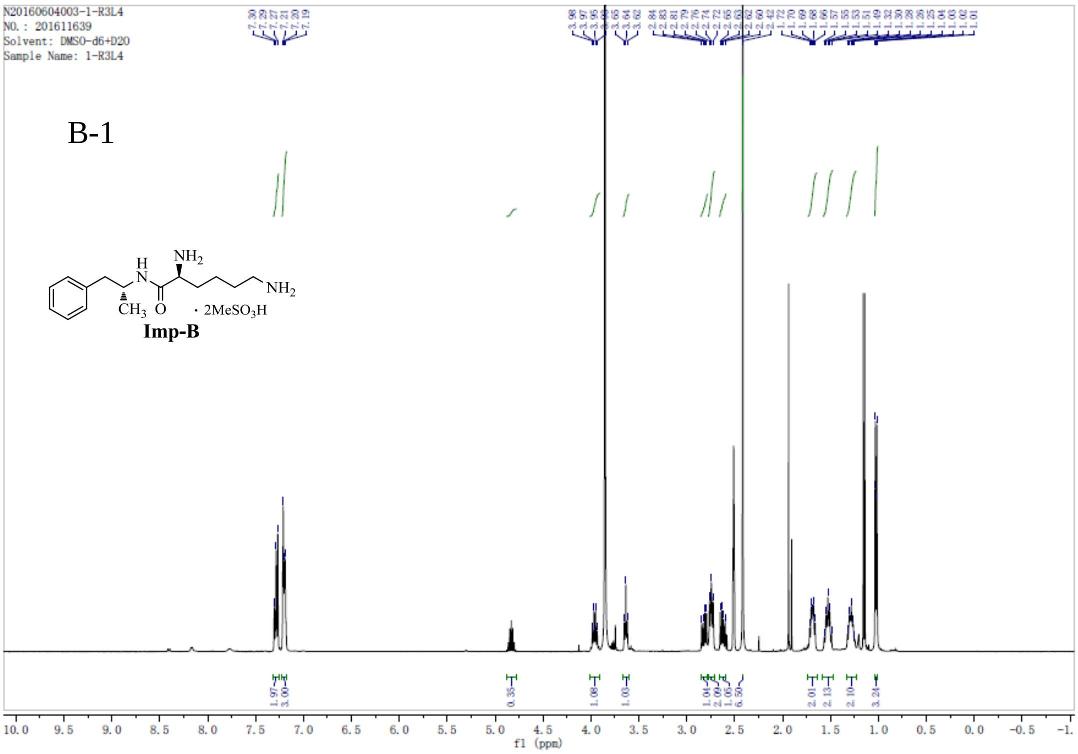

Imp-B ^{1}H NMR DMSO 400 MHz

C-1

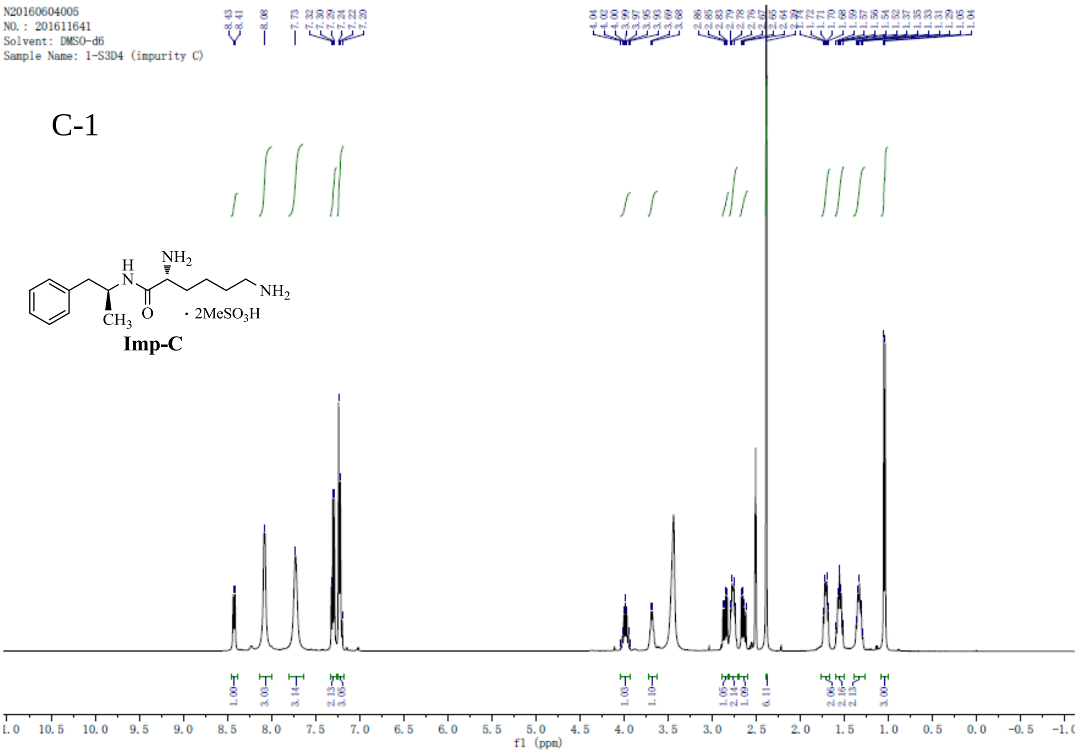

Imp-C ^{1}H NMR DMSO 400 MHz

C-2

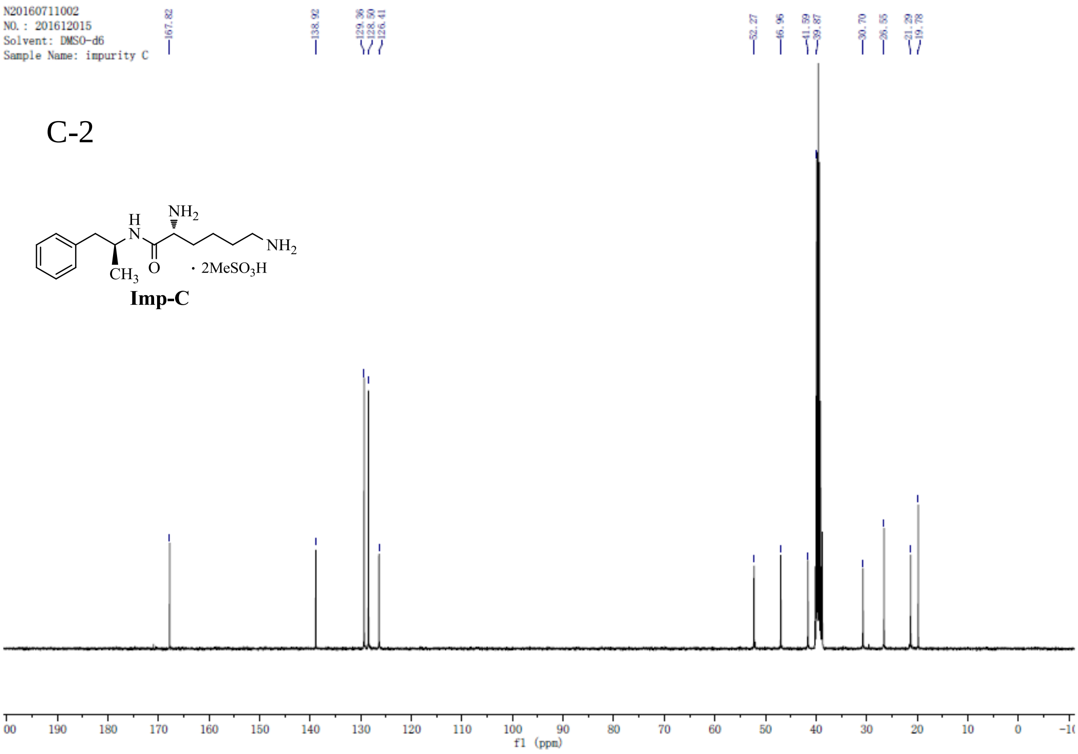

Imp-C ^{13}C NMR DMSO 101 MHz

C-3

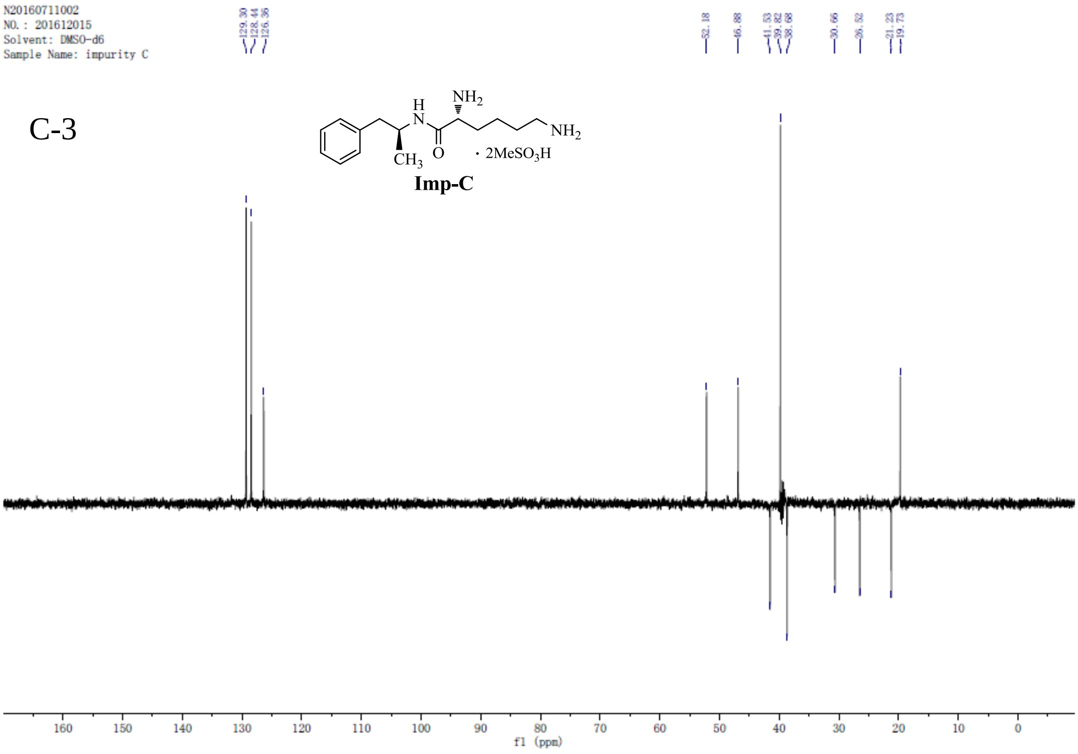

Imp-C DEPT DMSO 101 MHz

C-4

Qualitative Compound Report

**Data File**	20160630-015.d	**Sample Name**	Impurity C
**Sample Type**	Sample	**Position**	Vial 49
**Instrument Name**	Instrument 1	**User Name**	
**Acq Method**		**IRM Calibration Status**	Success
**DA Method**	MS.m	**Comment**	N20160630031

Compound Table

Compound Label	RT	Mass	Abund	Formula	Tgt Mass	Diff (ppm)
Cpd 1: C15 H25 N3 O	-0.25	263.1997	660907	C15 H25 N3 O	263.1998	-0.37

Compound Label	RT	Algorithm	Mass
Cpd 1: C15 H25 N3 O	-0.25	Find By Formula	263.1997

MS Zoomed Spectrum

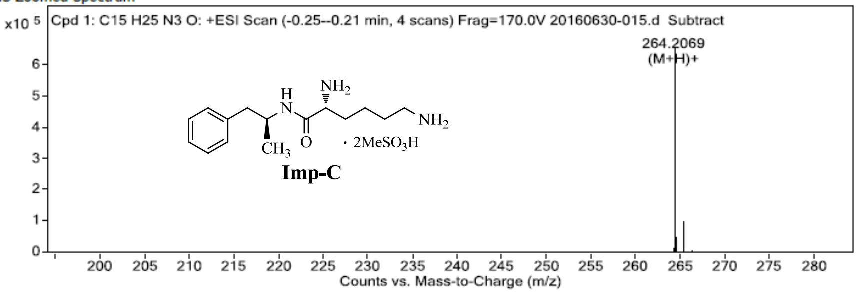

MS Spectrum Peak List

m/z	*Calc m/z*	Diff(ppm)	Abund	Formula	Ion
264.2069	264.207	-0.36	660907	C15 H26 N3 O	(M+H)+

--- End Of Report ---

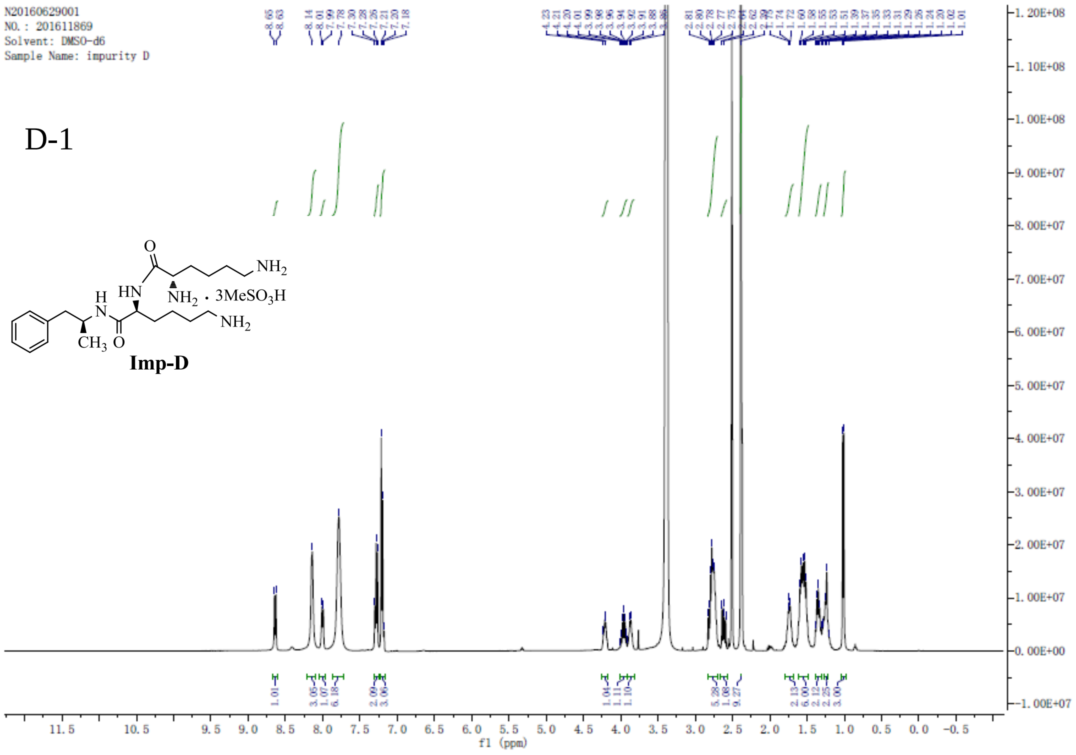

Imp-D ^{1}H NMR DMSO 400 MHz

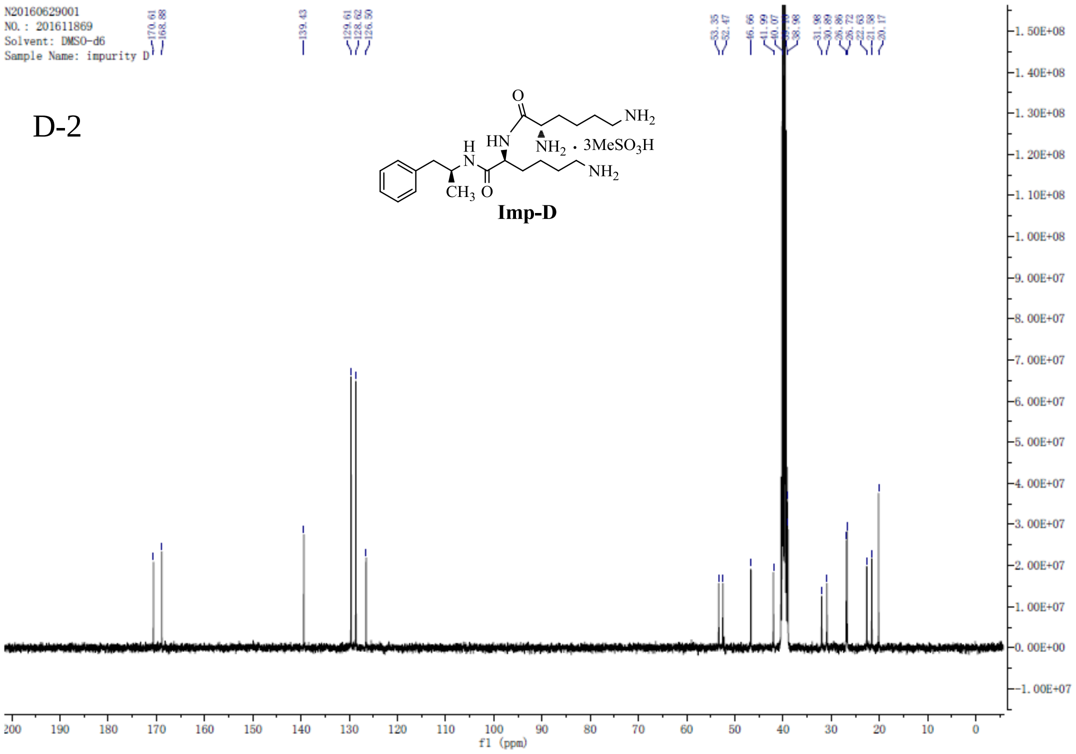

Imp-D ^{13}C NMR DMSO 101 MHz

D-3

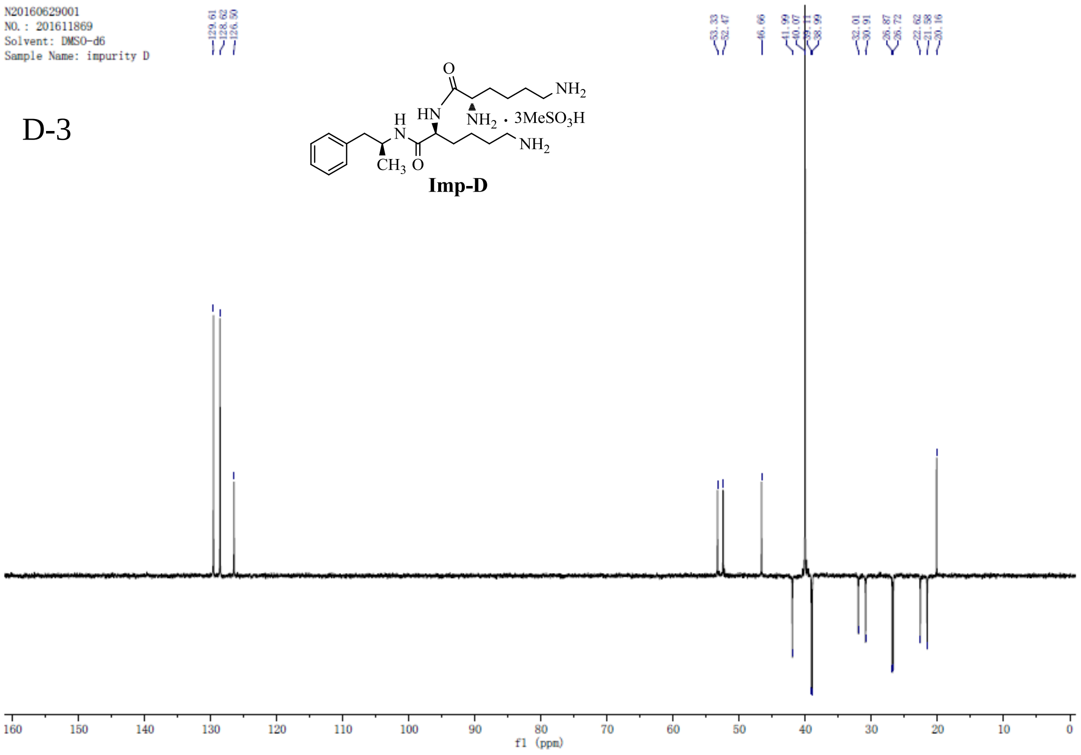

Imp-D DEPT DMSO 101 MHz

D-4

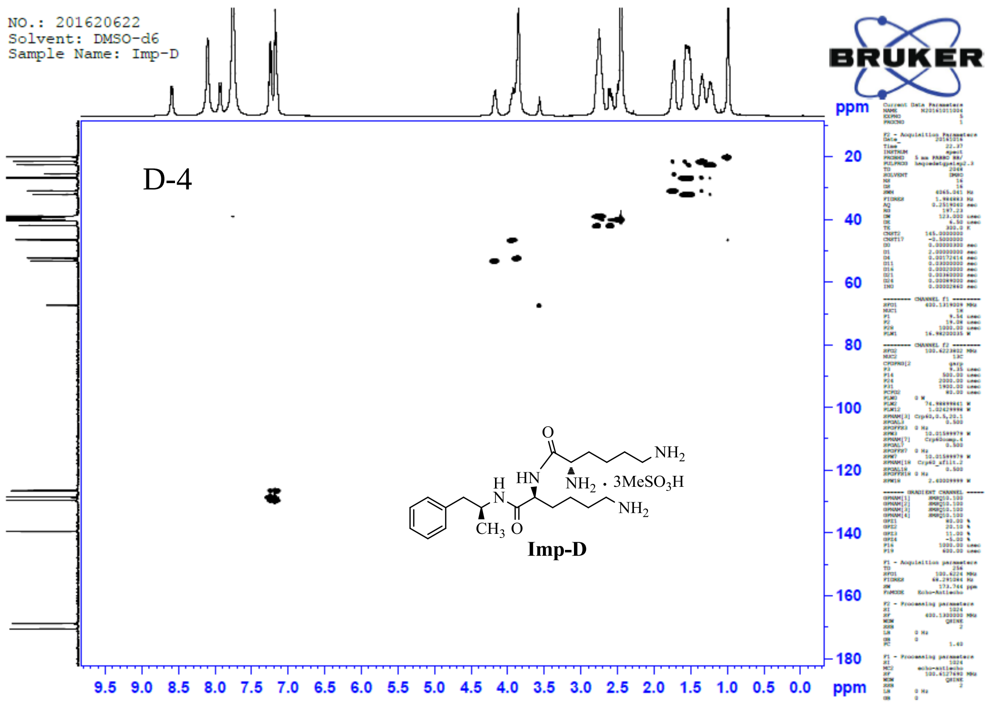

HSQC Imp-D DMSO

D-5

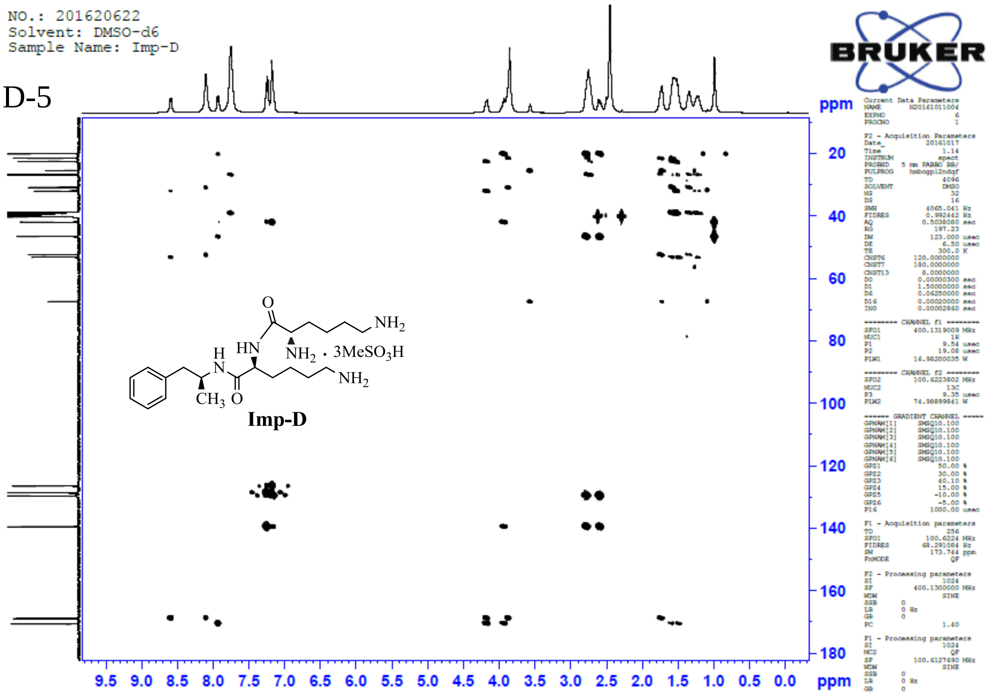

HMBC Imp-D DMSO

D-6

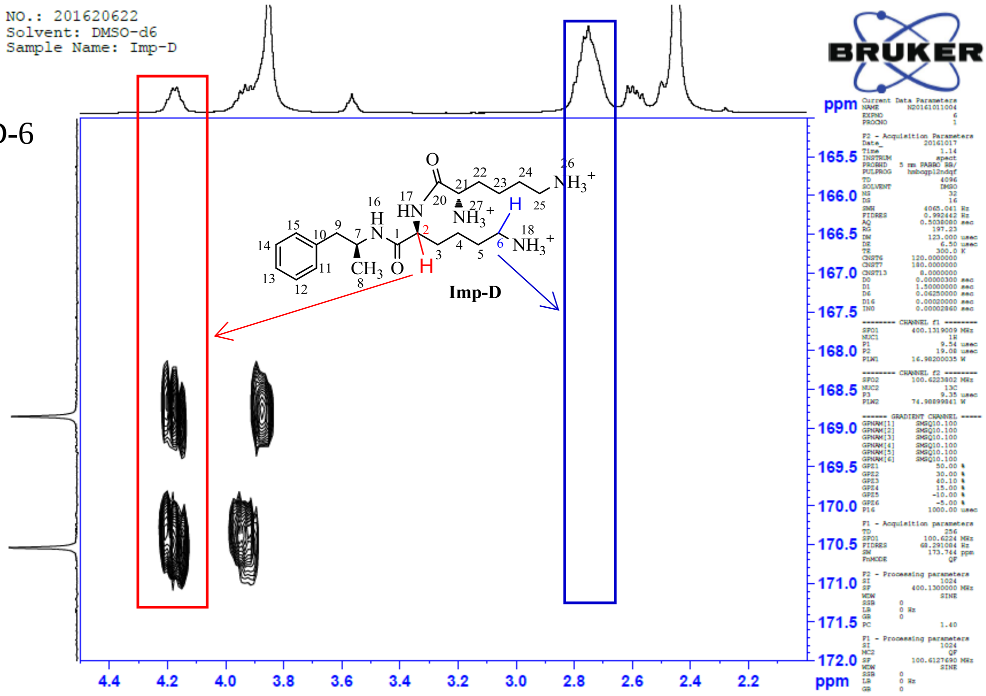

The the enlarged view of the HMBC spectrum of Imp-D

D-7

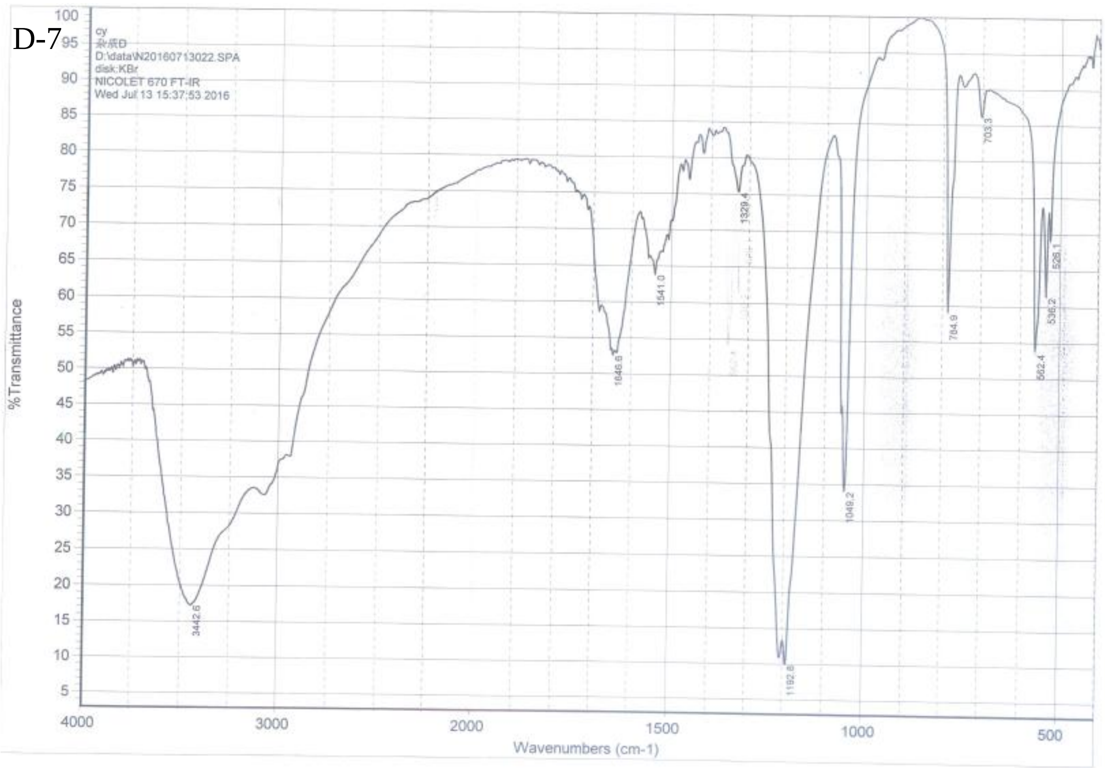

Imp-D IR

D-8

Qualitative Compound Report

**Data File**	20160701-001.d	**Sample Name**	Impurity D
**Sample Type**	Sample	**Position**	Vial 52
**Instrument Name**	Instrument 1	**User Name**	
**Acq Method**		**IRM Calibration Status**	Success
**DA Method**	MS.m	**Comment**	N20160627018

Compound Table

Compound Label	RT	Mass	Abund	Formula	Tgt Mass	Diff (ppm)
Cpd 1: C21 H37 N5 O2	-0.32	391.2952	239384	C21 H37 N5 O2	391.2947	1.31

Compound Label	RT	Algorithm	Mass
Cpd 1: C21 H37 N5 O2	-0.32	Find By Formula	391.2952

MS Zoomed Spectrum

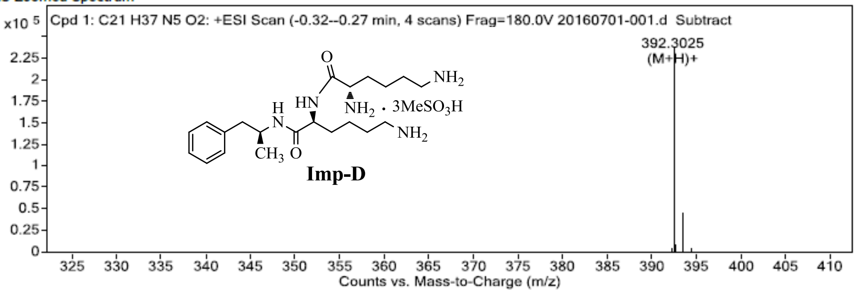

MS Spectrum Peak List

m/z	*Calc m/z*	Diff(ppm)	Abund	Formula	Ion
392.3025	392.302	1.32	239384	C21 H38 N5 O2	(M+H)+

--- End Of Report ---

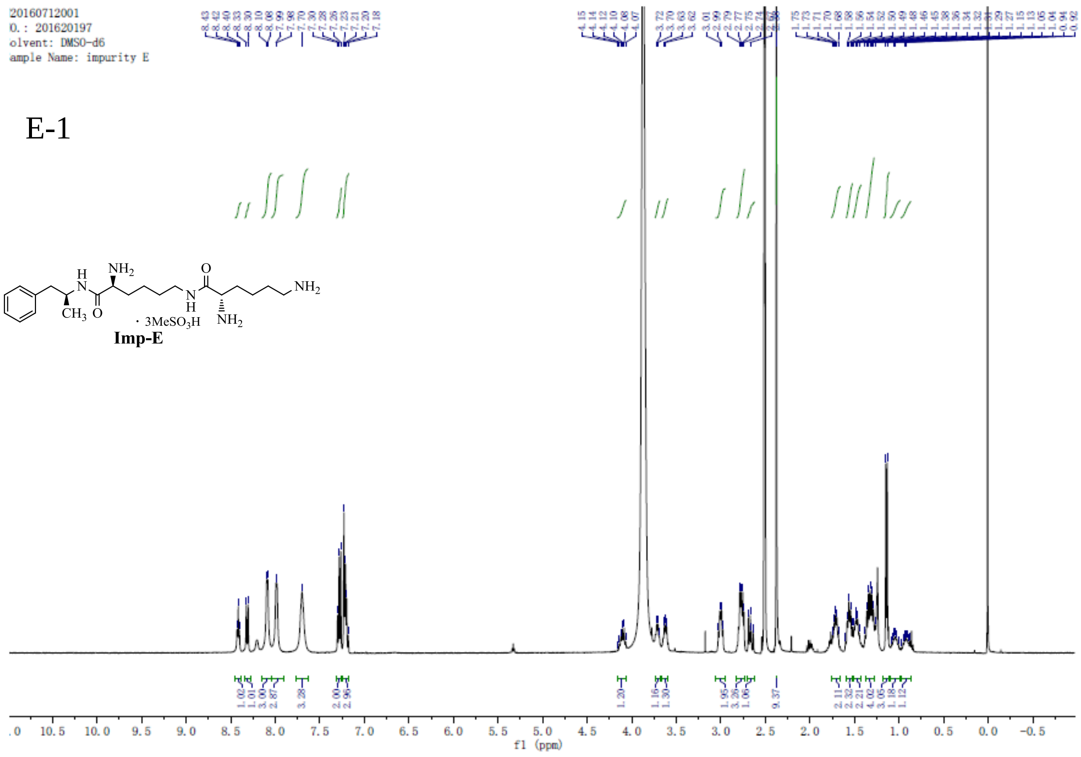

Imp-E ^{1}H NMR DMSO 400 MHz

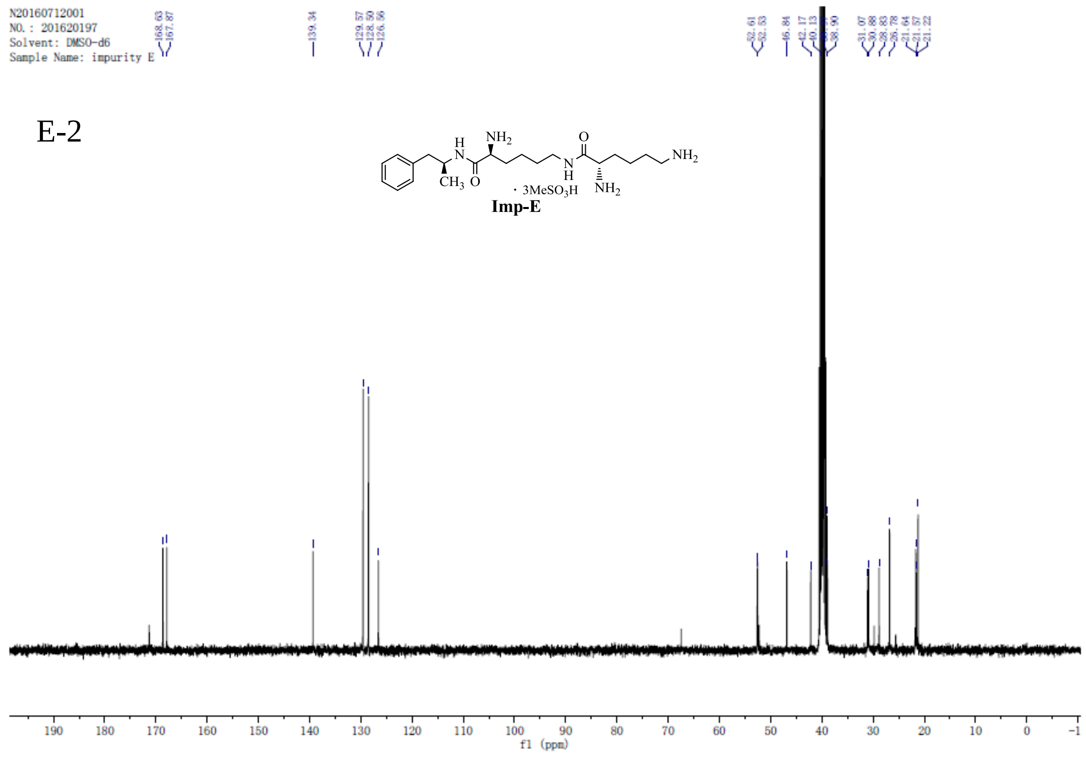

Imp-E ^{13}C NMR DMSO 101 MHz

E-3

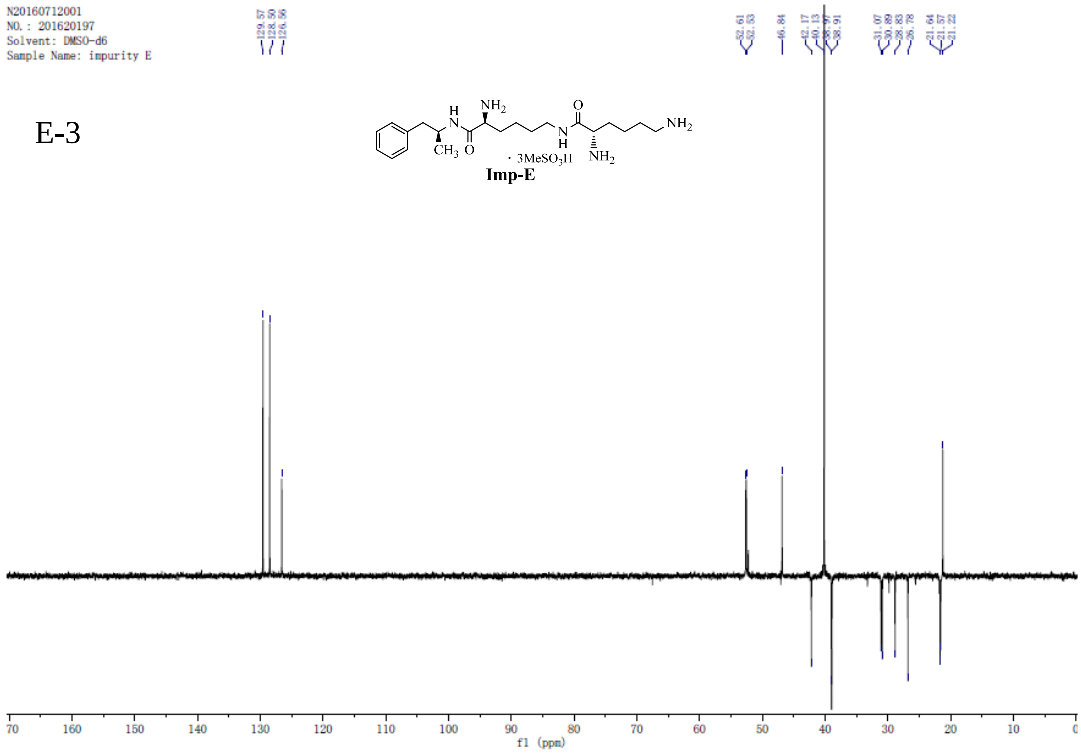

Imp-E DEPT DMSO 101 MHz

E-4

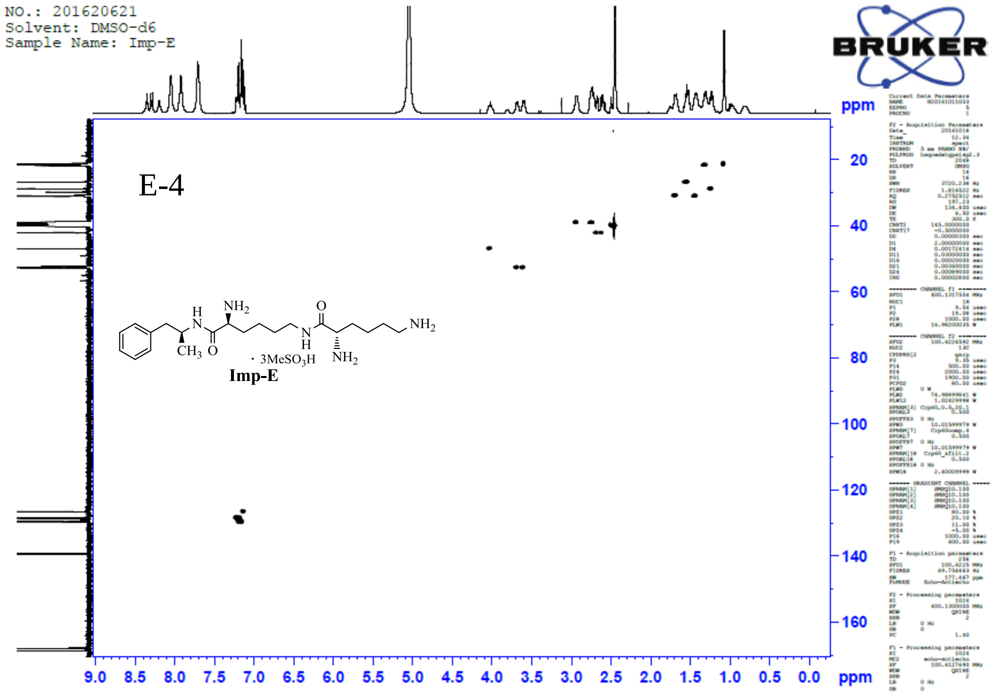

HSQC Imp-E DMSO

E-5

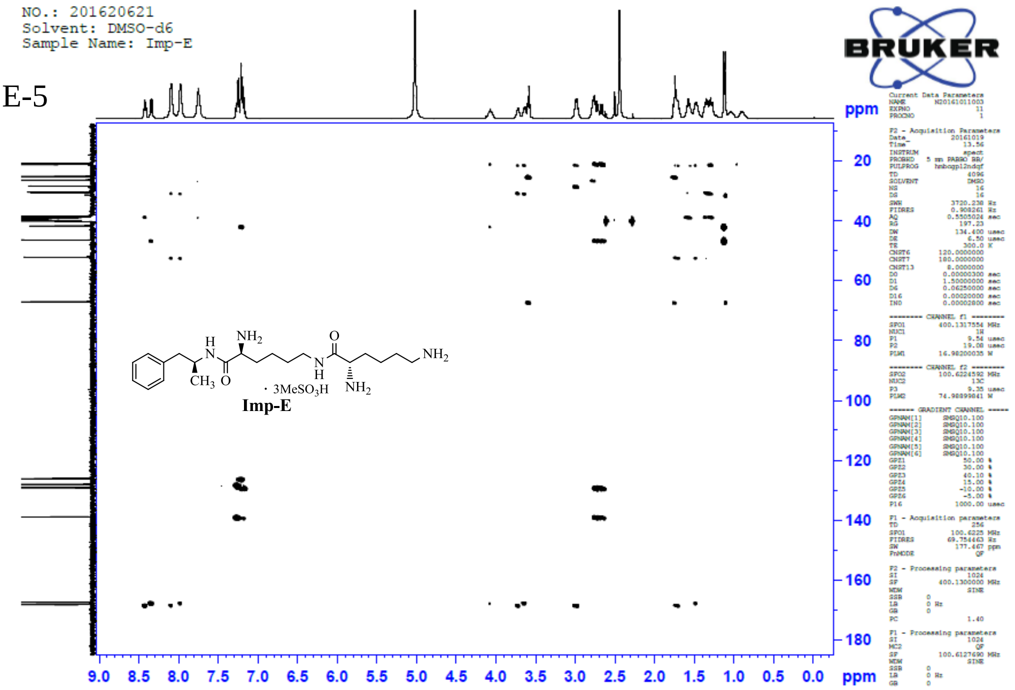

HMBC Imp-E DMSO

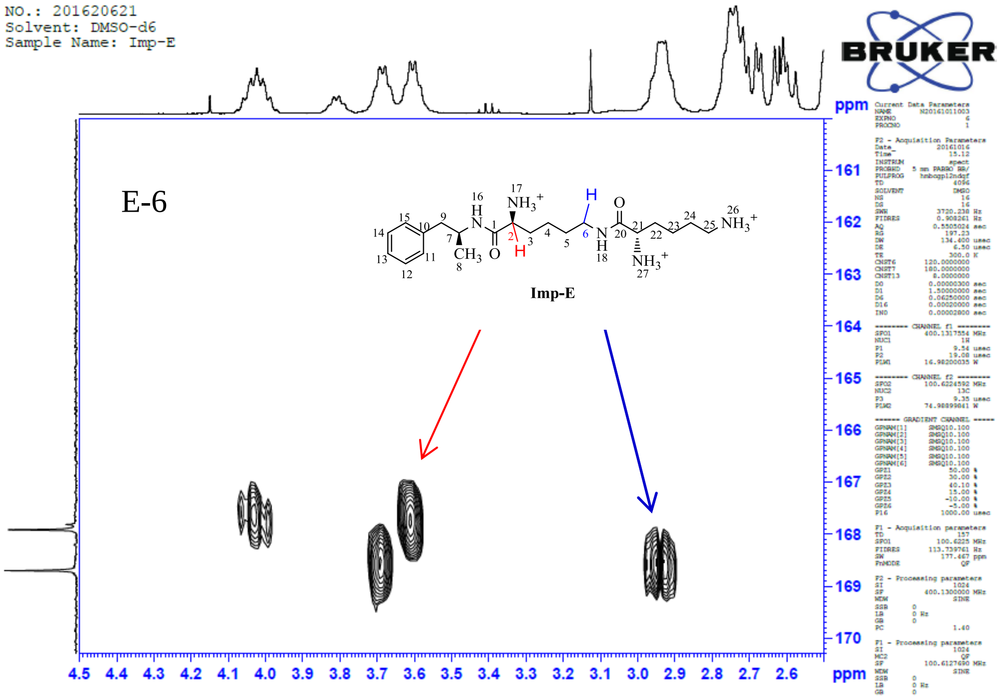

The the enlarged view of the HMBC spectrum of Imp-E

E-7

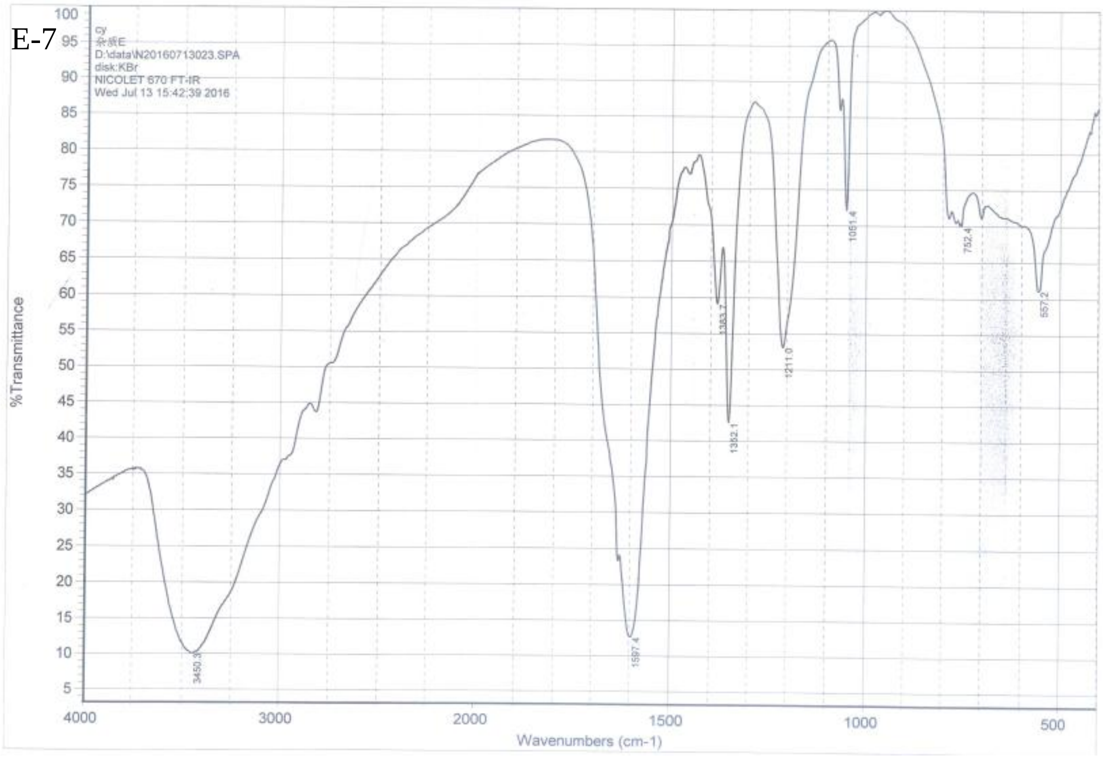

Imp-E IR

E-8

Qualitative Compound Report

**Data File**	20160630-019.d	**Sample Name**	Impurity E
**Sample Type**	Sample	**Position**	Vial 51
**Instrument Name**	Instrument 1	**User Name**	
**Acq Method**		**IRM Calibration Status**	Success
**DA Method**	MS.m	**Comment**	N20160627019

Compound Table

Compound Label	RT	Mass	Abund	Formula	Tgt Mass	Diff (ppm)
Cpd 1: C21 H37 N5 O2	-0.05	391.2954	66624	C21 H37 N5 O2	391.2947	1.66

Compound Label	RT	Algorithm	Mass
Cpd 1: C21 H37 N5 O2	-0.05	Find By Formula	391.2954

MS Zoomed Spectrum

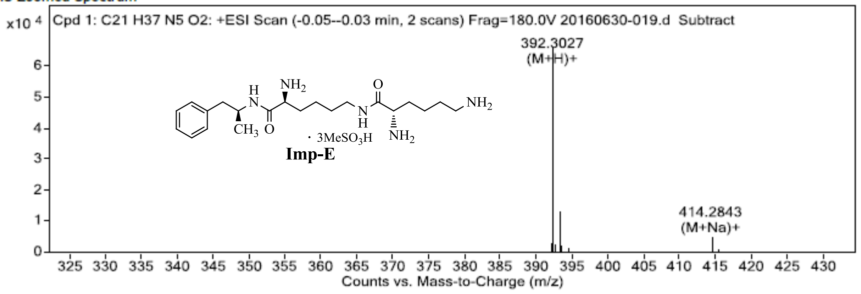

MS Spectrum Peak List

m/z	*Calc m/z*	Diff(ppm)	z	Abund	Formula	Ion
392.3027	392.302	1.66		66624	C21 H38 N5 O2	(M+H)+
414.2843	414.2839	0.82	1	4866	C21 H37 N5 Na O2	(M+Na)+

Imp-E HRMS

F-1

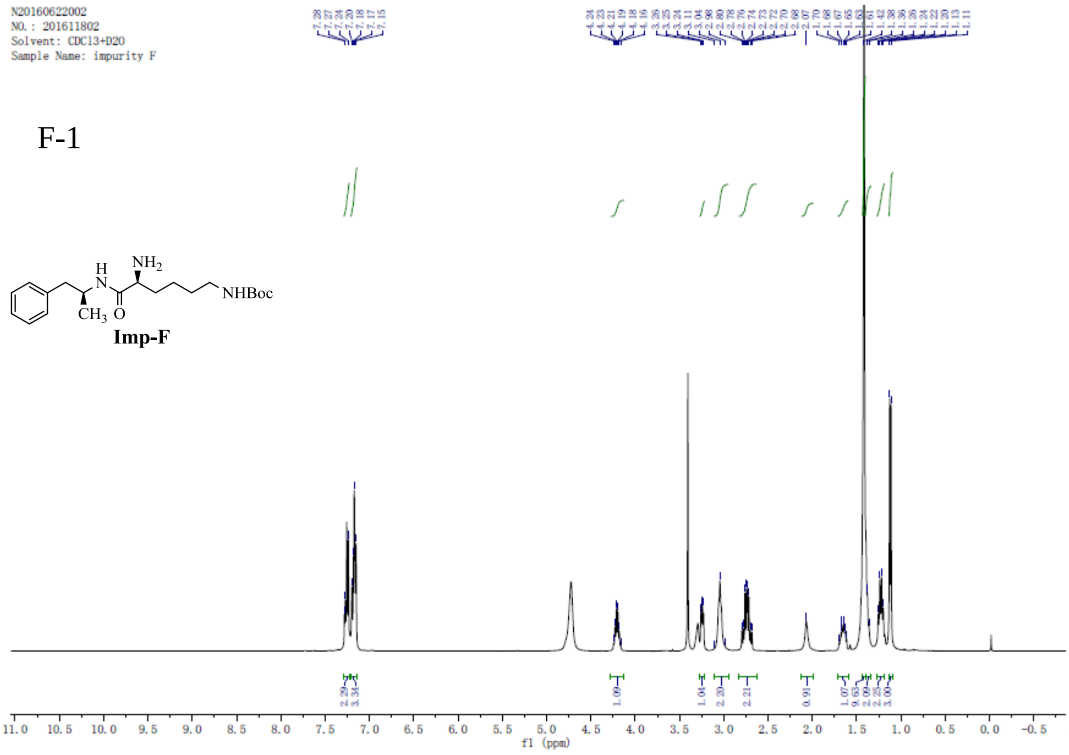

Imp-F ^1H NMR CDCl3 400 MHz

F-2

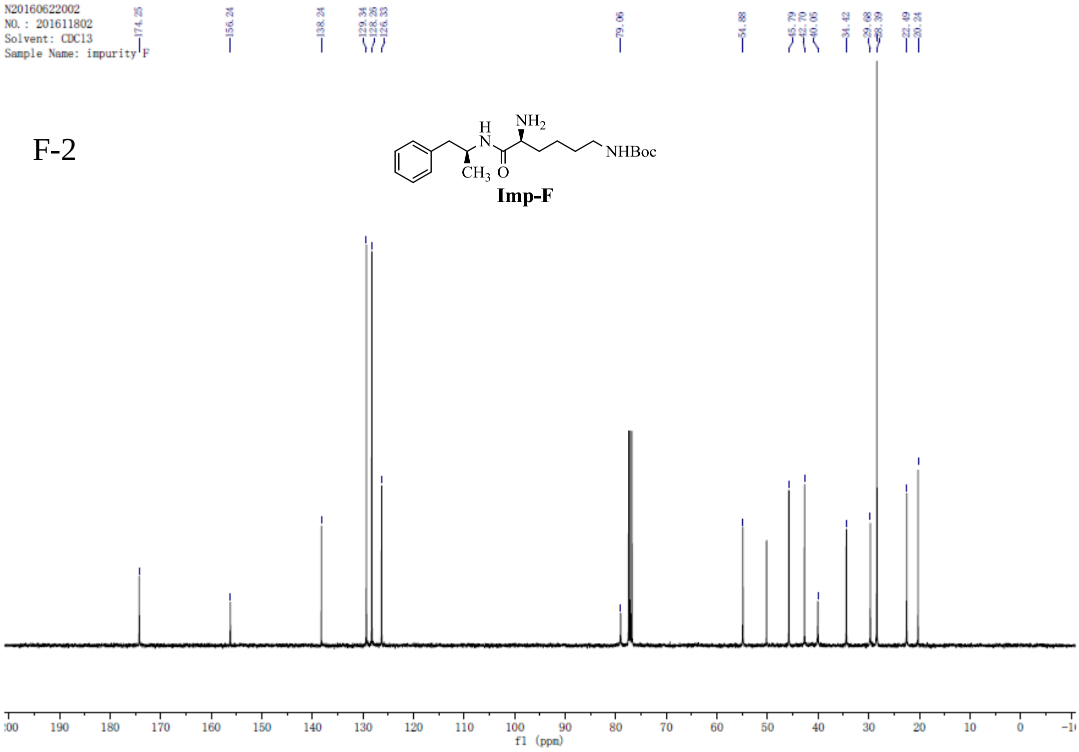

Imp-F ^{13}C NMR CDCl3 101 MHz

F-3

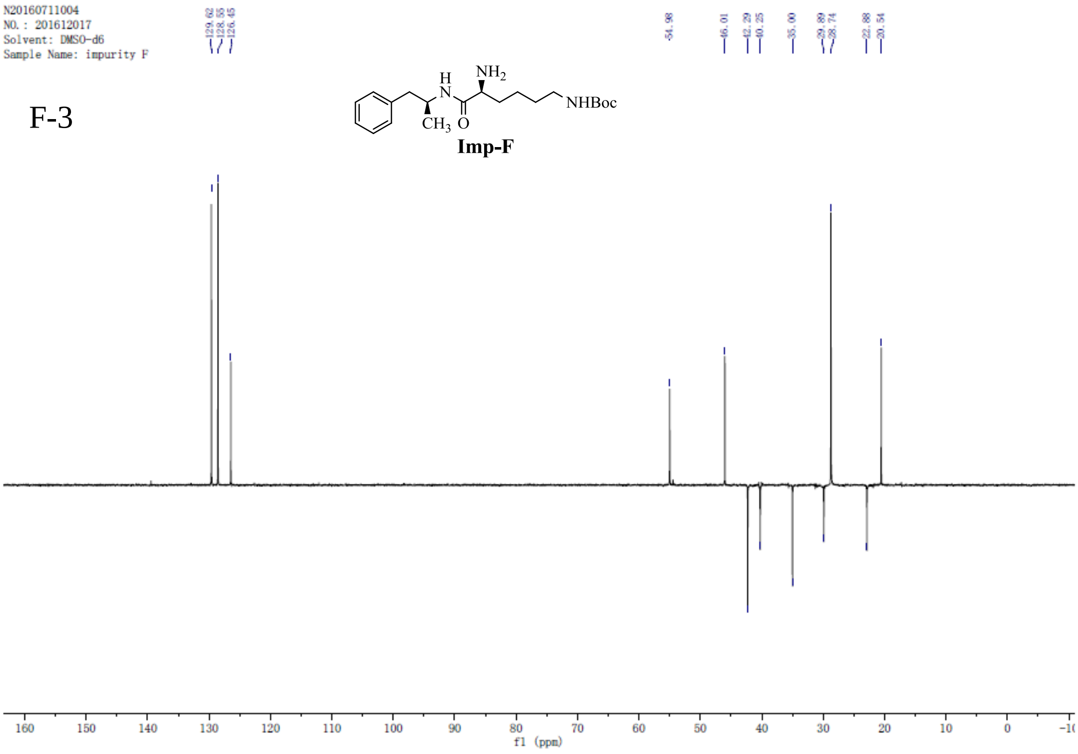

Imp-F DEPT $CDCl_3$ 101 MHz

F-4

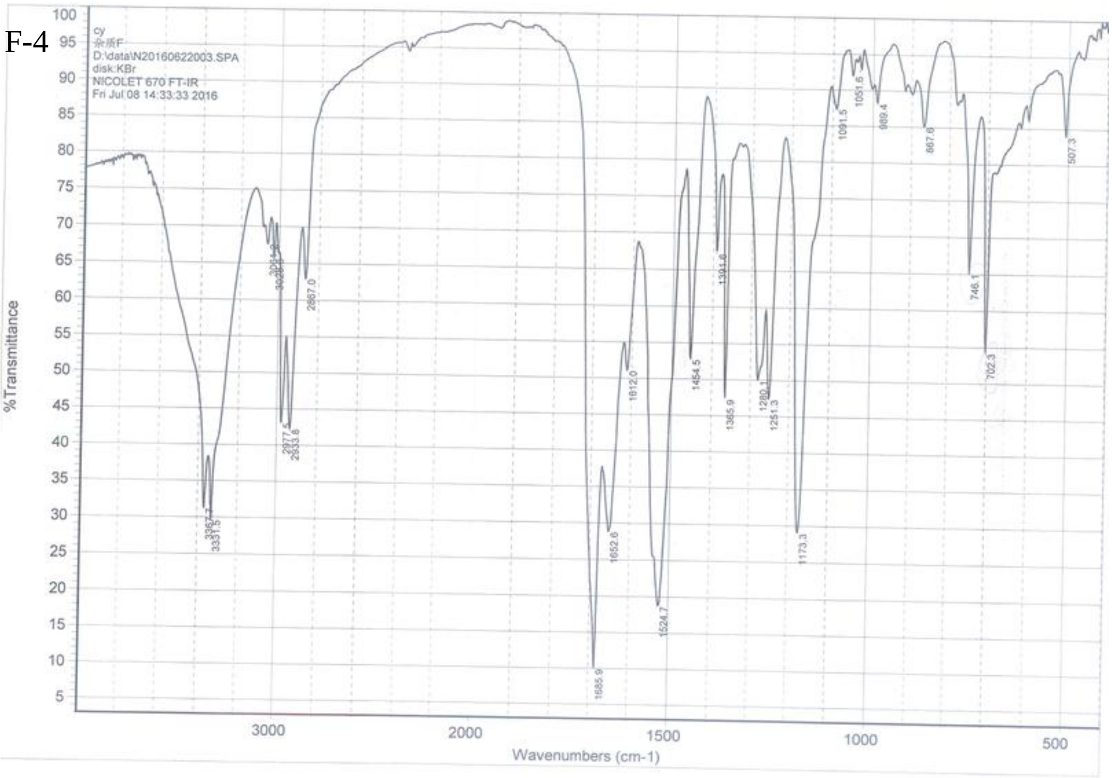

Imp-F IR

F-5

Qualitative Compound Report

**Data File**	20160701-003.d	**Sample Name**	Impurity F
**Sample Type**	Sample	**Position**	Vial 53
**Instrument Name**	Instrument 1	**User Name**	
**Acq Method**		**IRM Calibration Status**	Success
**DA Method**	MS.m	**Comment**	N20160622003

Compound Table

Compound Label	RT	Mass	Abund	Formula	Tgt Mass	Diff (ppm)
Cpd 1: C20 H33 N3 O3	-0.25	363.2522	324479	C20 H33 N3 O3	363.2522	-0.03

Compound Label	RT	Algorithm	Mass
Cpd 1: C20 H33 N3 O3	-0.25	Find By Formula	363.2522

MS Zoomed Spectrum

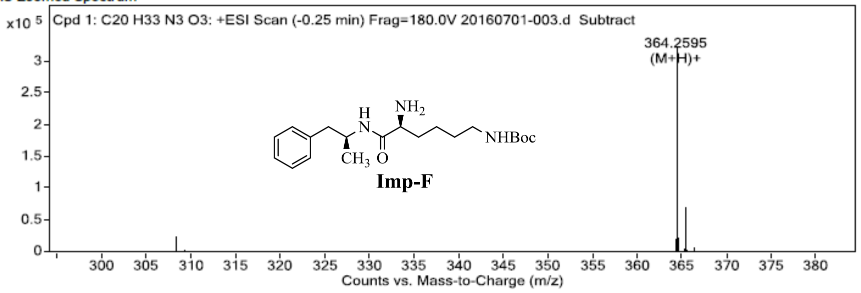

MS Spectrum Peak List

m/z	Calc m/z	Diff(ppm)	Abund	Formula	Ion
364.2595	364.2595	-0.01	324479	C20 H34 N3 O3	(M+H)+

Imp-F HRMS

G-1

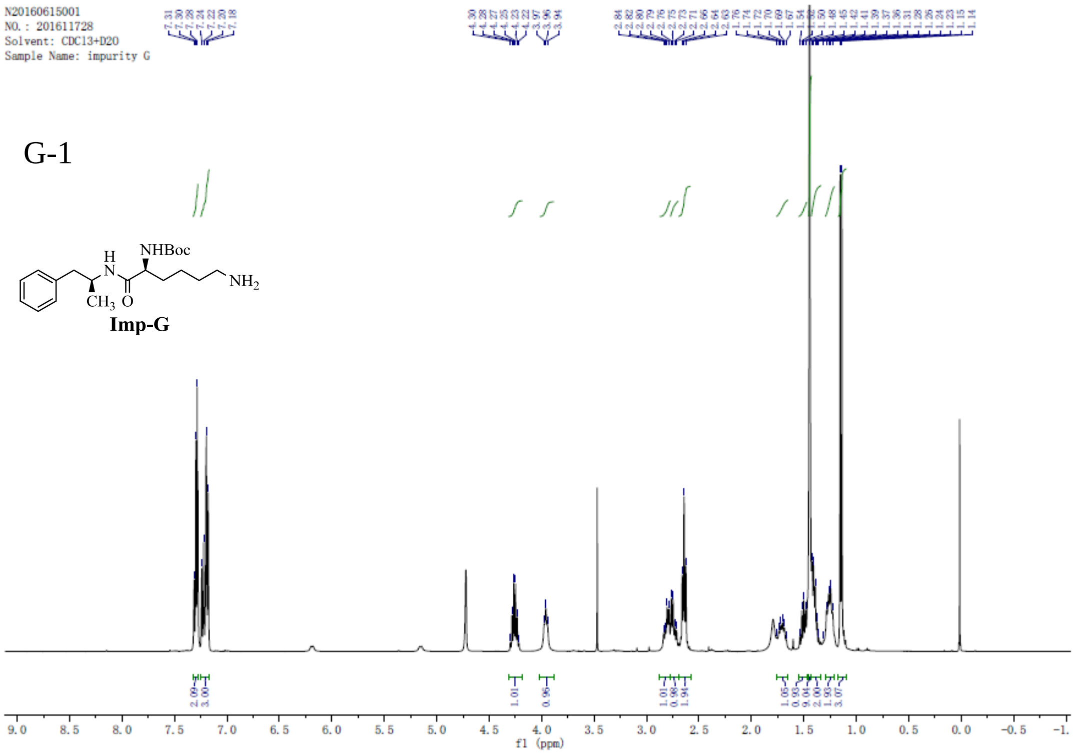

Imp-G ^{1}H NMR $CDCl_3$ 400 MHz

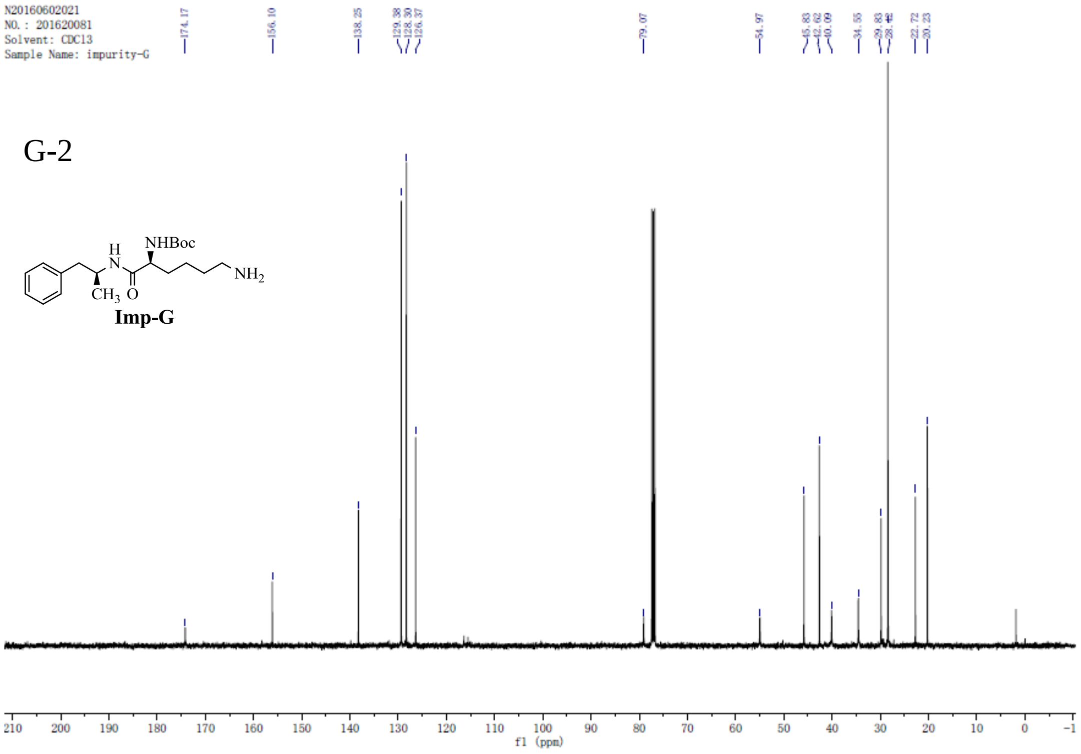

Imp-G ^{13}C NMR $CDCl_3$ 101 MHz

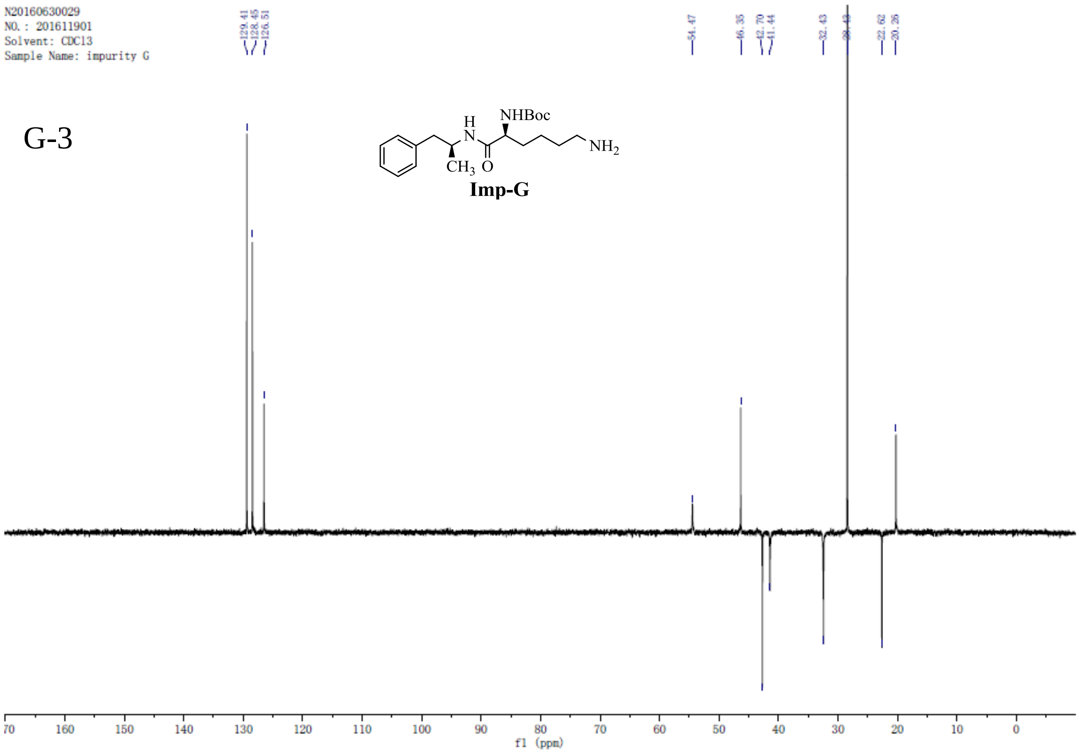

Imp-G DEPT $CDCl_3$ 101 MHz

G-4

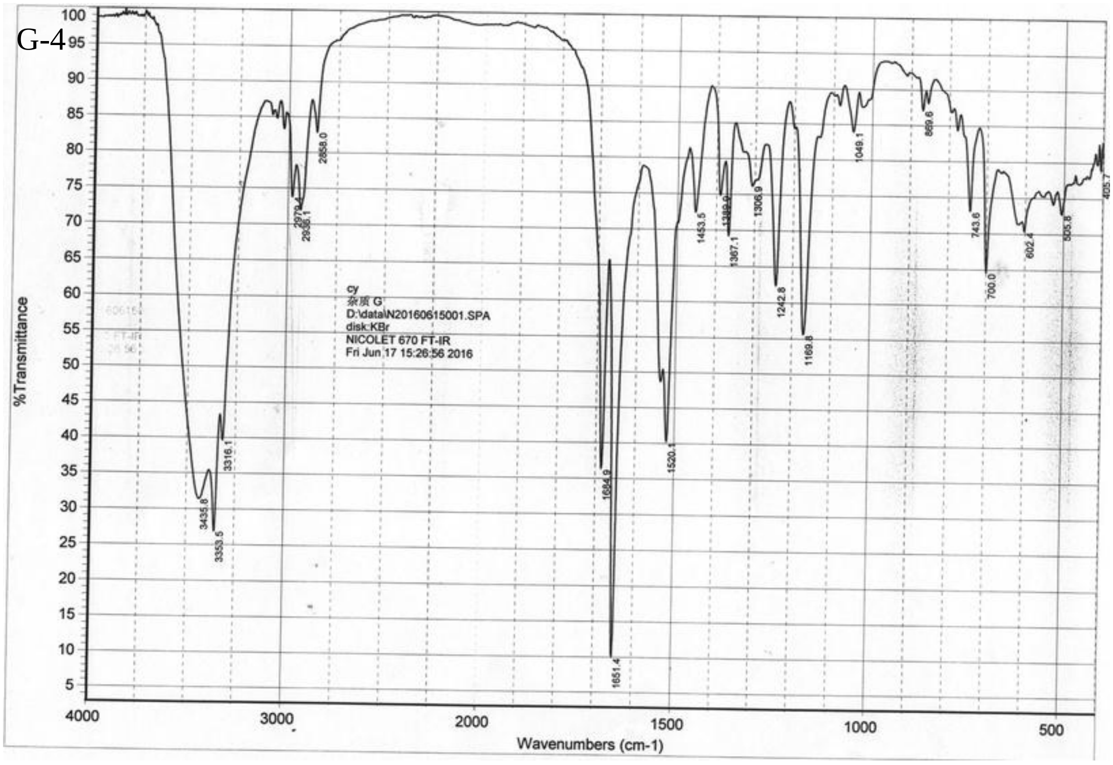

Imp-G IR

G-5

Qualitative Compound Report

**Data File**	20160701-006.d	**Sample Name**	Impurity G
**Sample Type**	Sample	**Position**	Vial 55
**Instrument Name**	Instrument 1	**User Name**	
**Acq Method**		**IRM Calibration Status**	Success
**DA Method**	MS.m	**Comment**	N20160615001

Compound Table

Compound Label	RT	Mass	Abund	Formula	Tgt Mass	Diff (ppm)
Cpd 1: C20 H33 N3 O3	0.06	363.2517	165528	C20 H33 N3 O3	363.2522	-1.27

Compound Label	RT	Algorithm	Mass
Cpd 1: C20 H33 N3 O3	0.06	Find By Formula	363.2517

MS Zoomed Spectrum

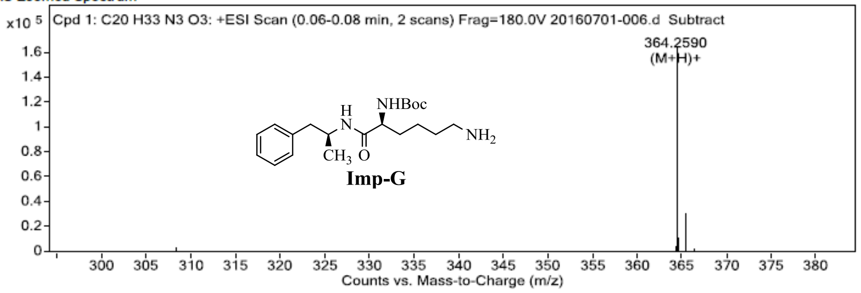

MS Spectrum Peak List

m/z	Calc m/z	Diff(ppm)	Abund	Formula	Ion
364.259	364.2595	-1.27	165528	C20 H34 N3 O3	(M+H)+

Imp-G HRMS

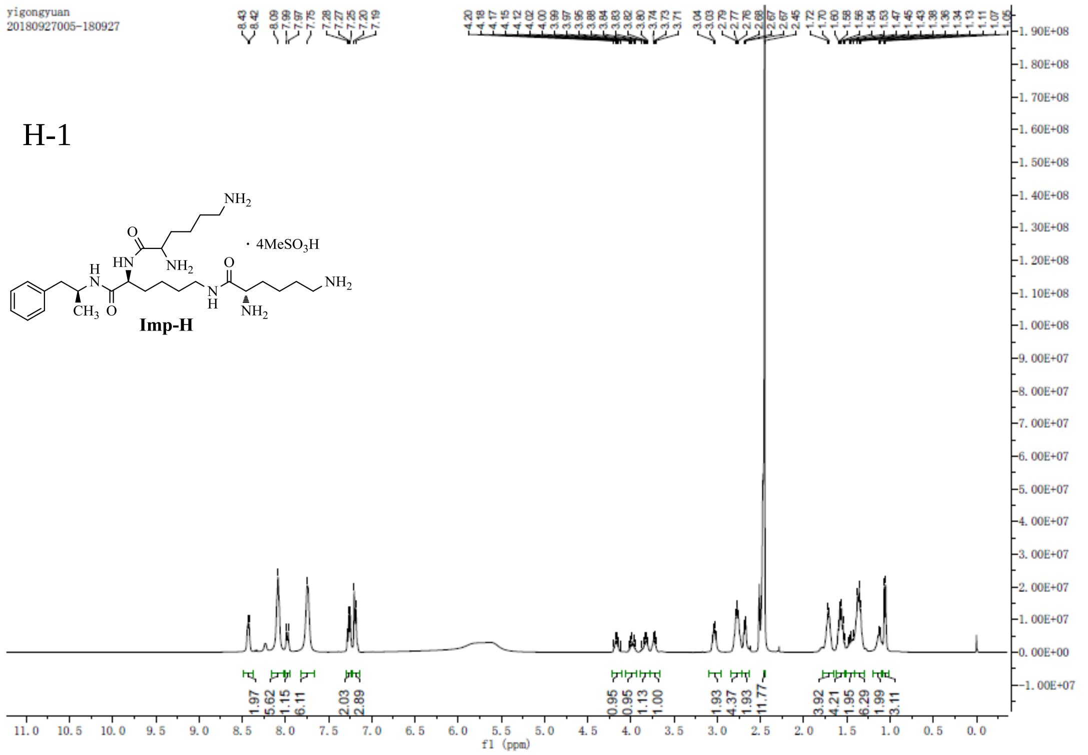

Imp-H ^{1}H NMR DMSO 400 MHz

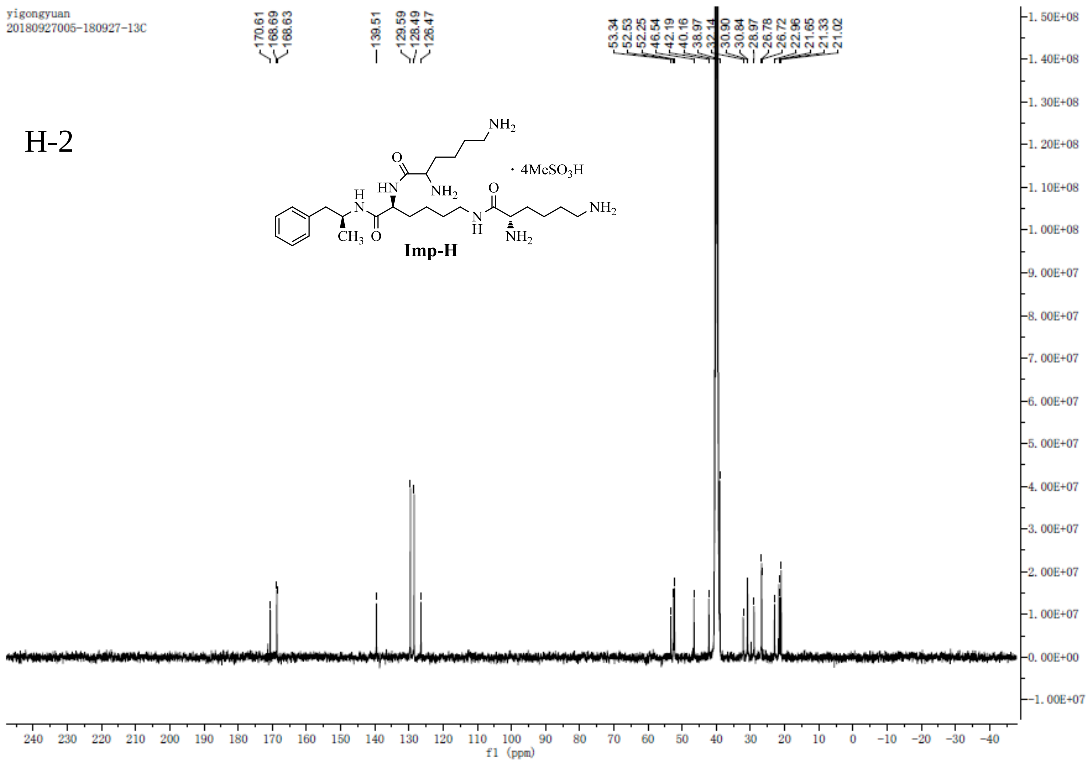

Imp-H ^{13}C NMR DMSO 101 MHz

H-3

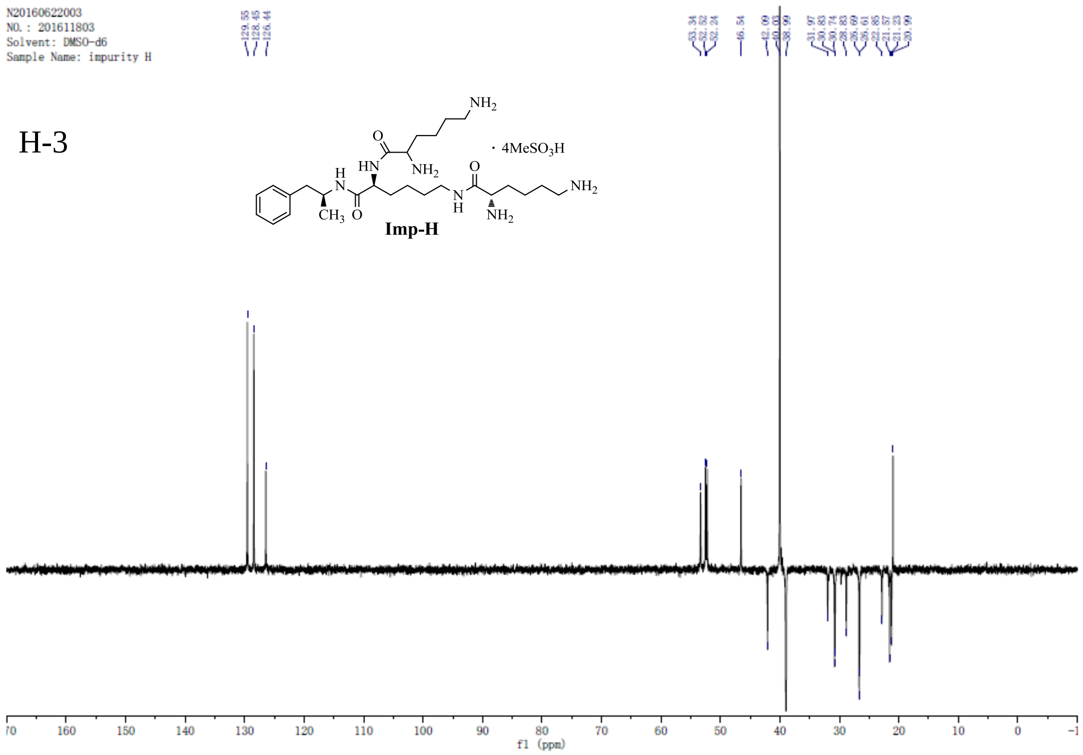

Imp-H DEPT DMSO 101 MHz

H-4

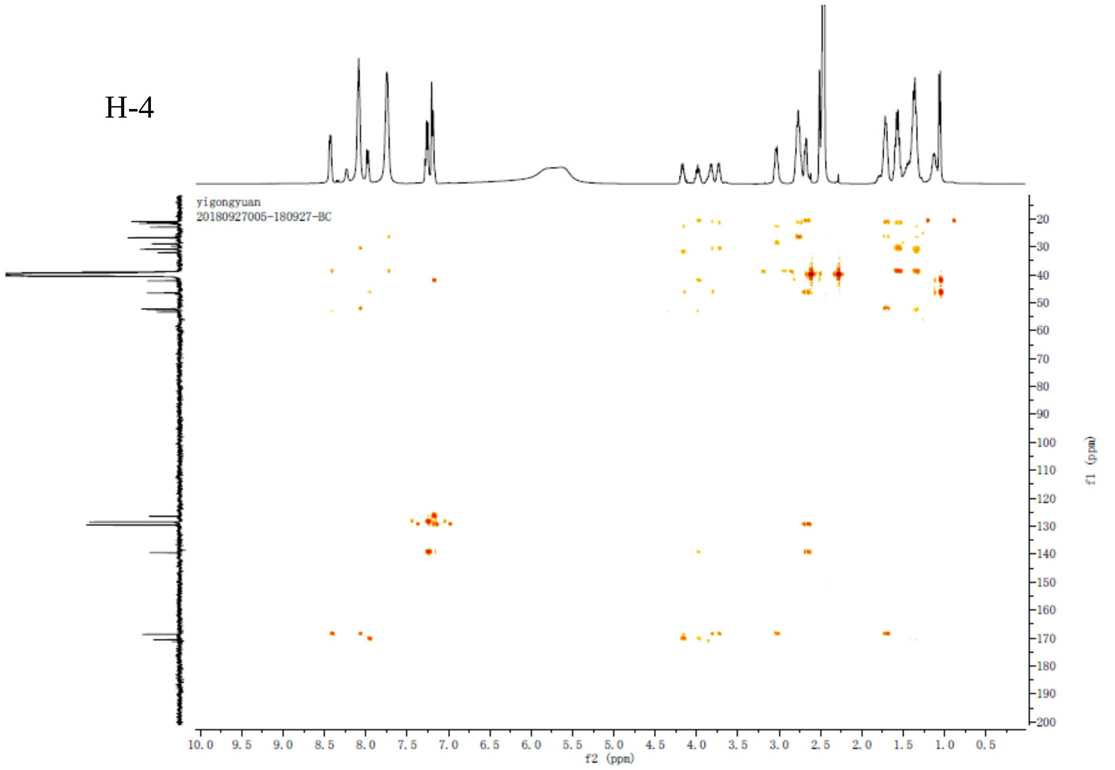

Imp-H HMBC DMSO

H-5

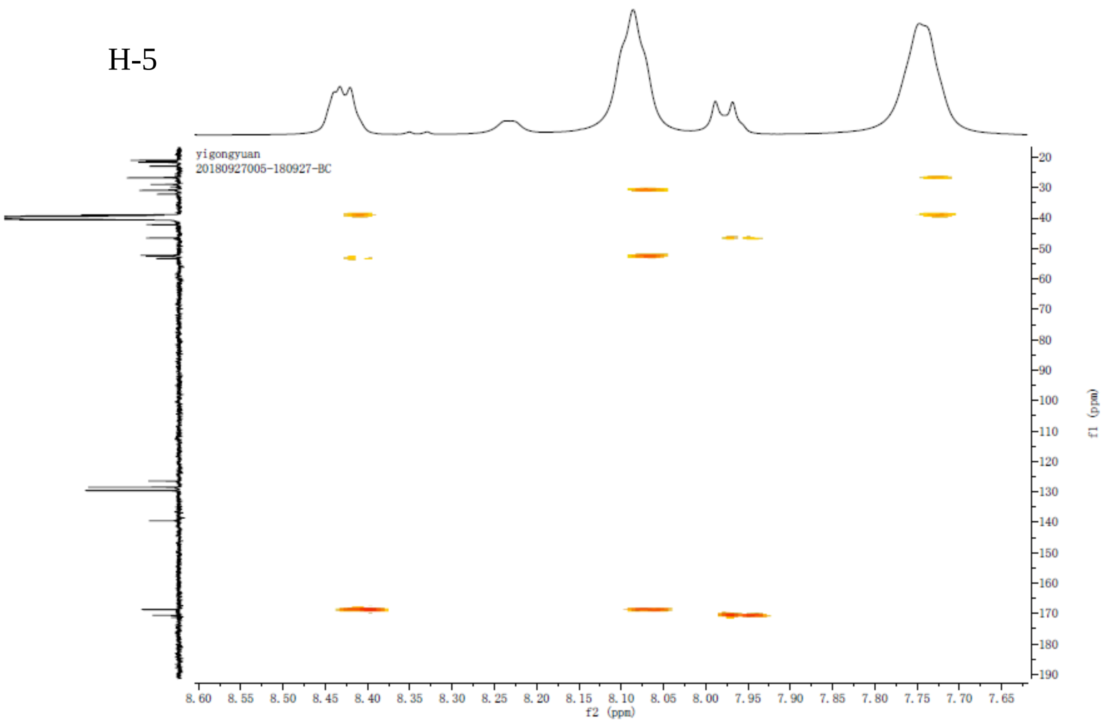

Imp-H the enlarged view of HMBC DMSO

H-6

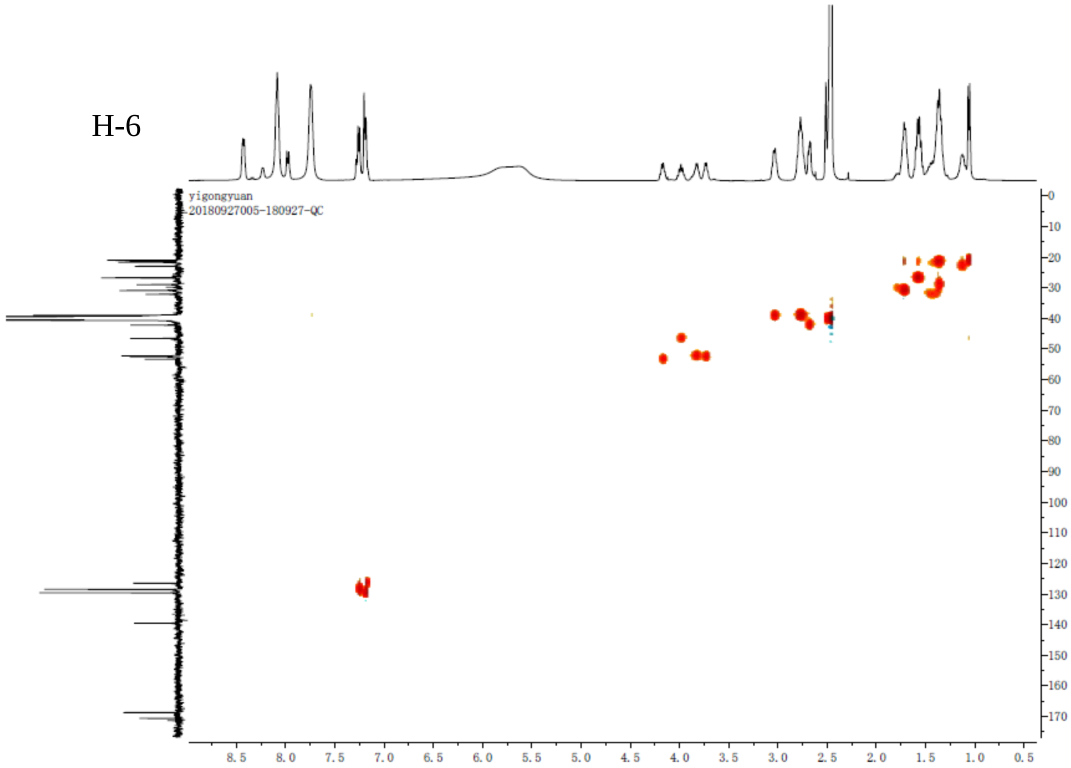

Imp-H HSQC DMSO

H-7

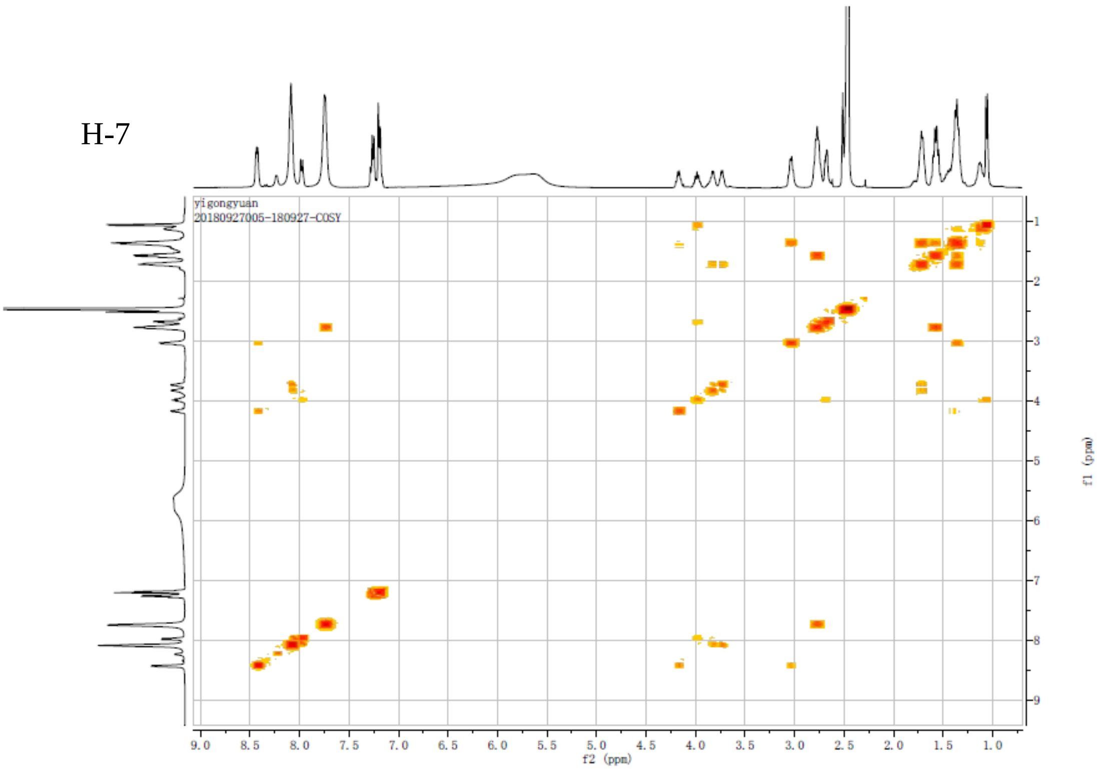

Imp-H COSY DMSO

H-8

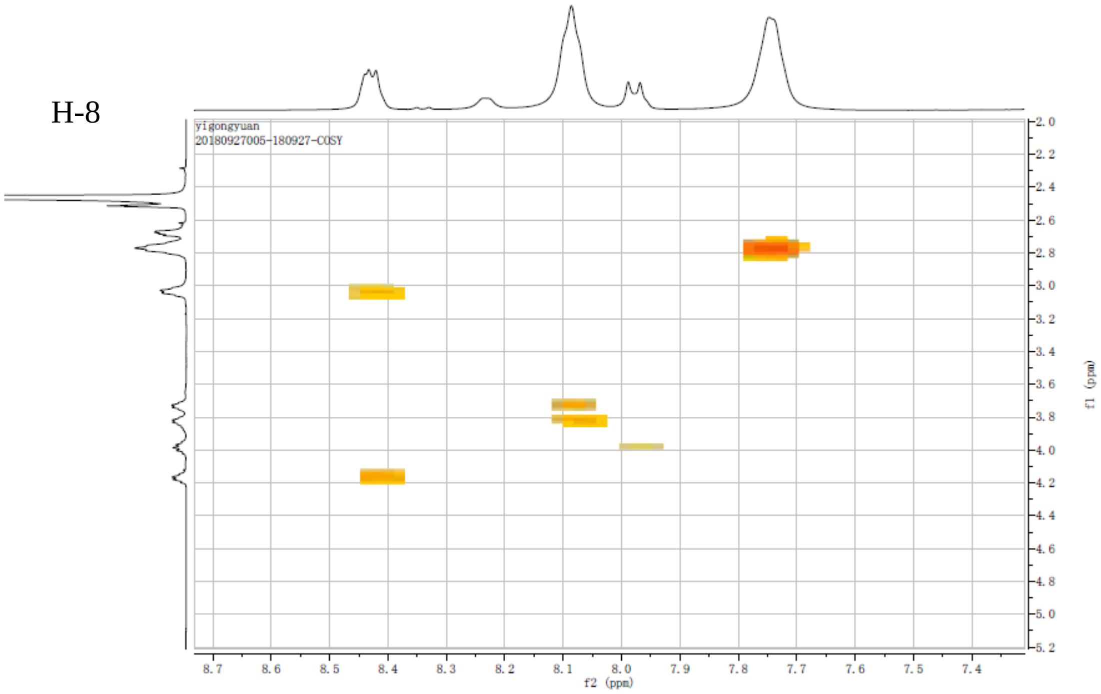

Imp-H the enlarged view of COSY DMSO

H-9

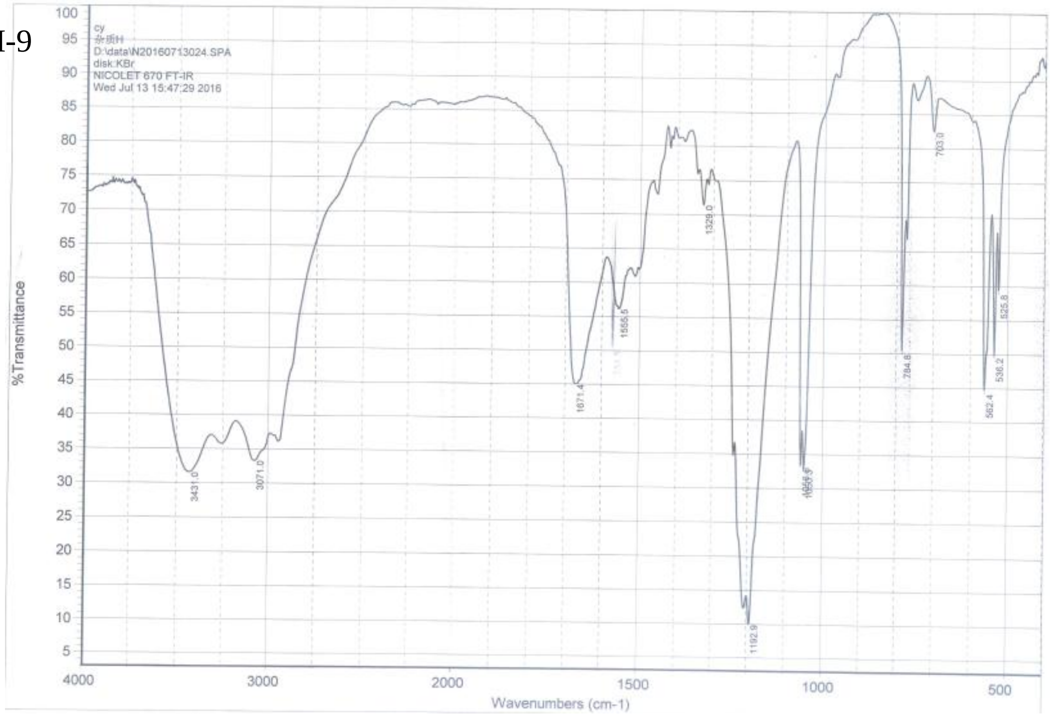

Imp-H IR

H-10

Qualitative Compound Report

**Data File**	20160701-008.d	**Sample Name**	Impurity H
**Sample Type**	Sample	**Position**	Vial 57
**Instrument Name**	Instrument 1	**User Name**	
**Acq Method**		**IRM Calibration Status**	Success
**DA Method**	MS.m	**Comment**	N20160622004

Compound Table

Compound Label	RT	Mass	Abund	Formula	Tgt Mass	Diff (ppm)
Cpd 1: C27 H49 N7 O3	-0.26	519.3903	91317	C27 H49 N7 O3	519.3897	1.13

Compound Label	RT	Algorithm	Mass
Cpd 1: C27 H49 N7 O3	-0.26	Find By Formula	519.3903

MS Zoomed Spectrum

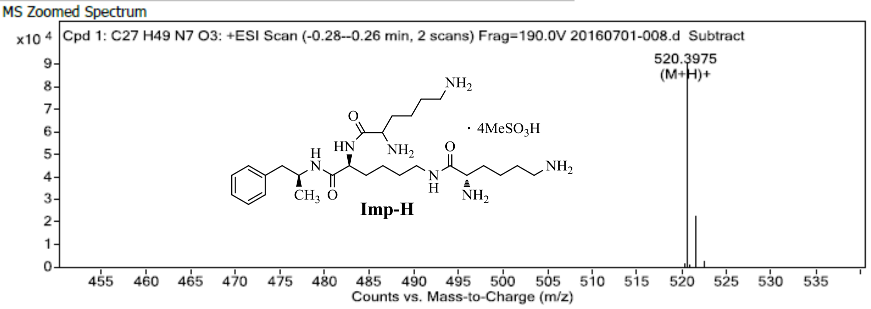

MS Spectrum Peak List

m/z	*Calc m/z*	Diff(ppm)	z	Abund	Formula	Ion
520.3975	520.397	1.12	1	91317	C27 H50 N7 O3	(M+H)+

Imp-H HRMS

J-1

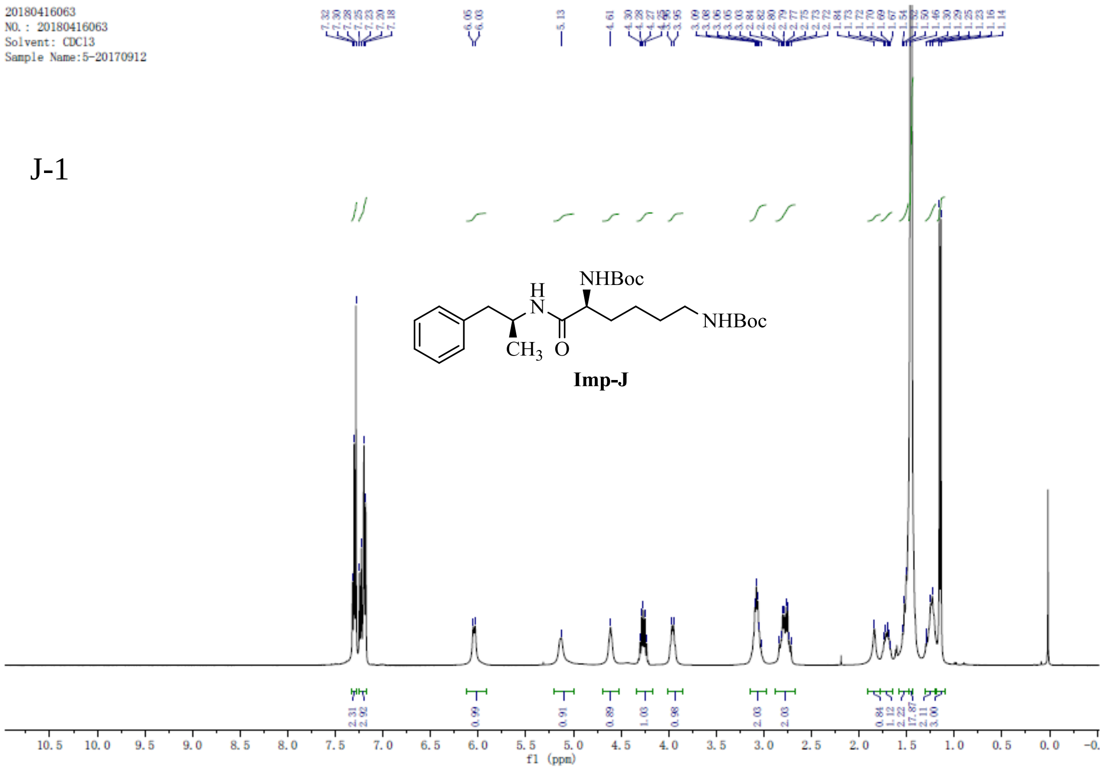

Imp-J ^{1}H NMR CDCl$_3$ 400 MHz

J-2

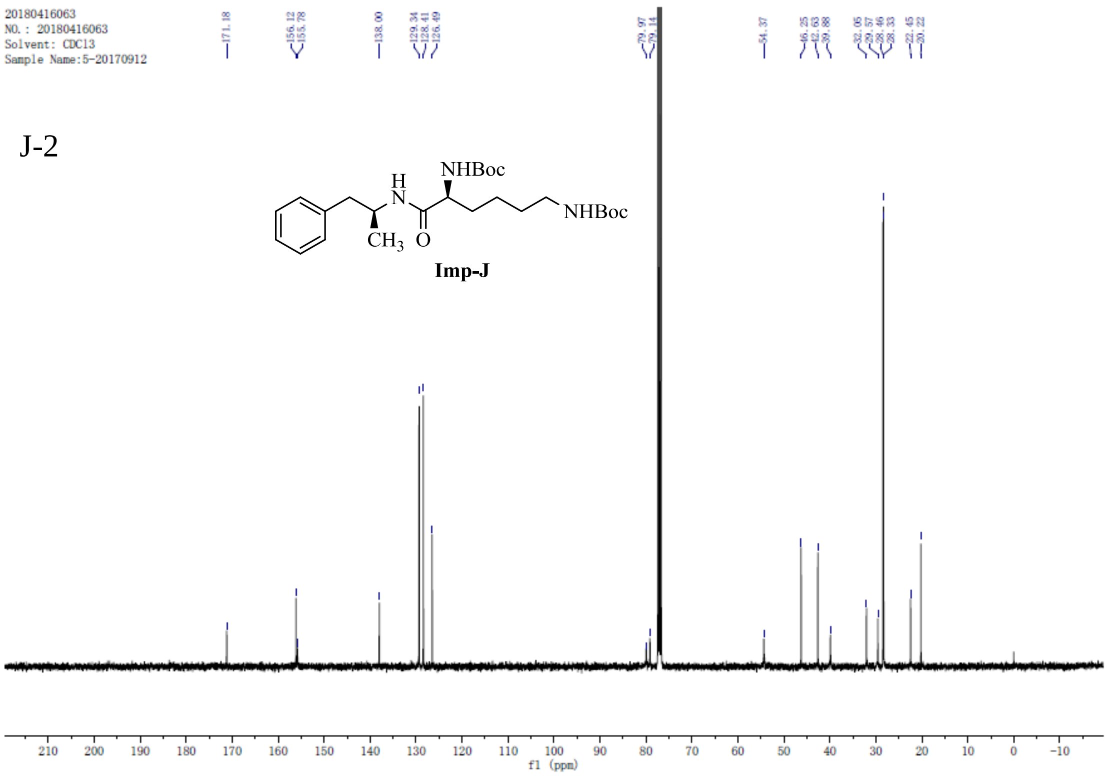

Imp-J ^{13}C NMR CDCl$_3$ 101 MHz

J-3

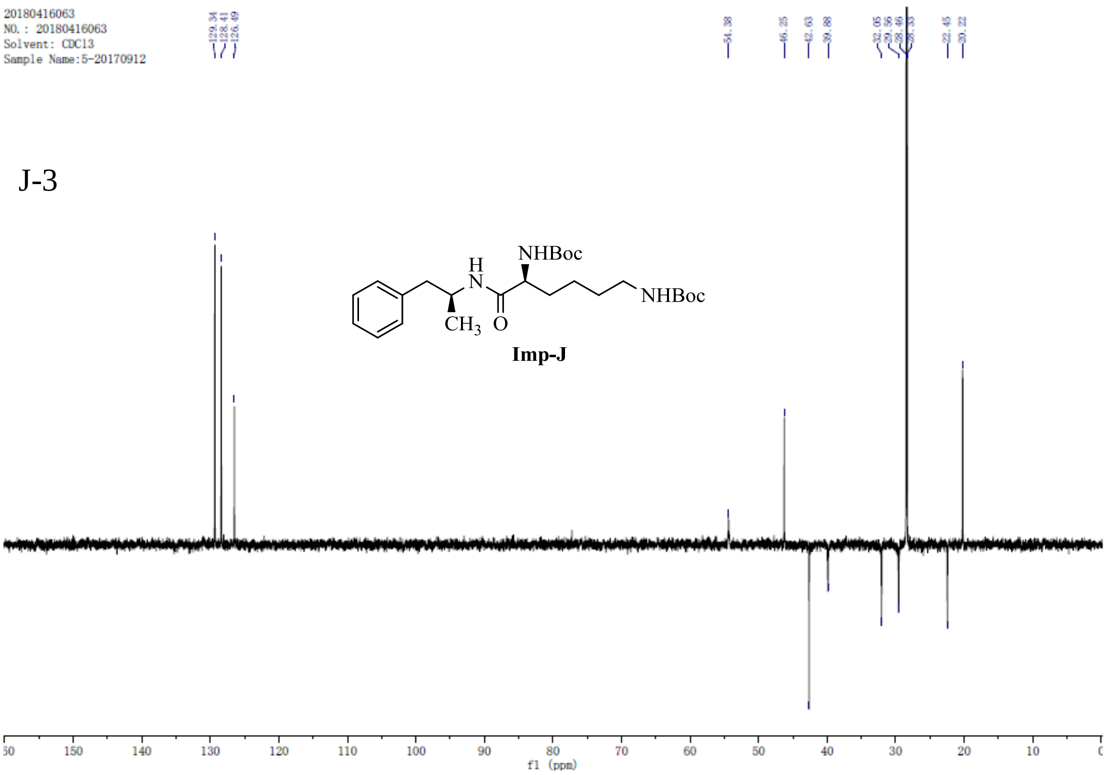

Imp-J DEPT $CDCl_3$ 101 MHz

J-4

Qualitative Compound Report

**Data File**	20160817-015.d	**Sample Name**	LYBBA-5
**Sample Type**	Sample	**Position**	Vial 14
**Instrument Name**	Instrument 1	**User Name**	
**Acq Method**		**IRM Calibration Status**	Success
**DA Method**	MS.m	**Comment**	N20160816003

Compound Table

Compound Label	RT	Mass	Abund	Formula	Tgt Mass	Diff (ppm)
Cpd 1: C25 H41 N3 O5	-0.04	463.3048	71303	C25 H41 N3 O5	463.3046	0.44

Compound Label	RT	Algorithm	Mass
Cpd 1: C25 H41 N3 O5	-0.04	Find By Formula	463.3048

MS Zoomed Spectrum

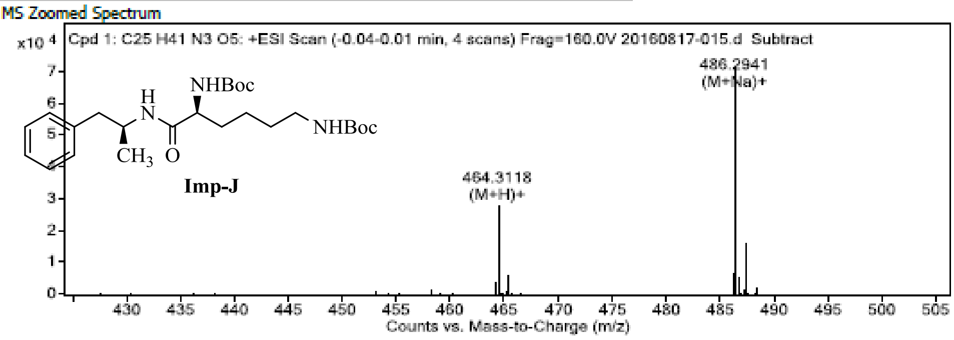

MS Spectrum Peak List

m/z	*Calc m/z*	Diff(ppm)	z	Abund	Formula	Ion
464.3118	464.3119	-0.3	1	28188	C25 H42 N3 O5	(M+H)+
486.2941	486.2938	0.48		71303	C25 H41 N3 Na O5	(M+Na)+

--- End Of Report ---

Imp-J HRMS

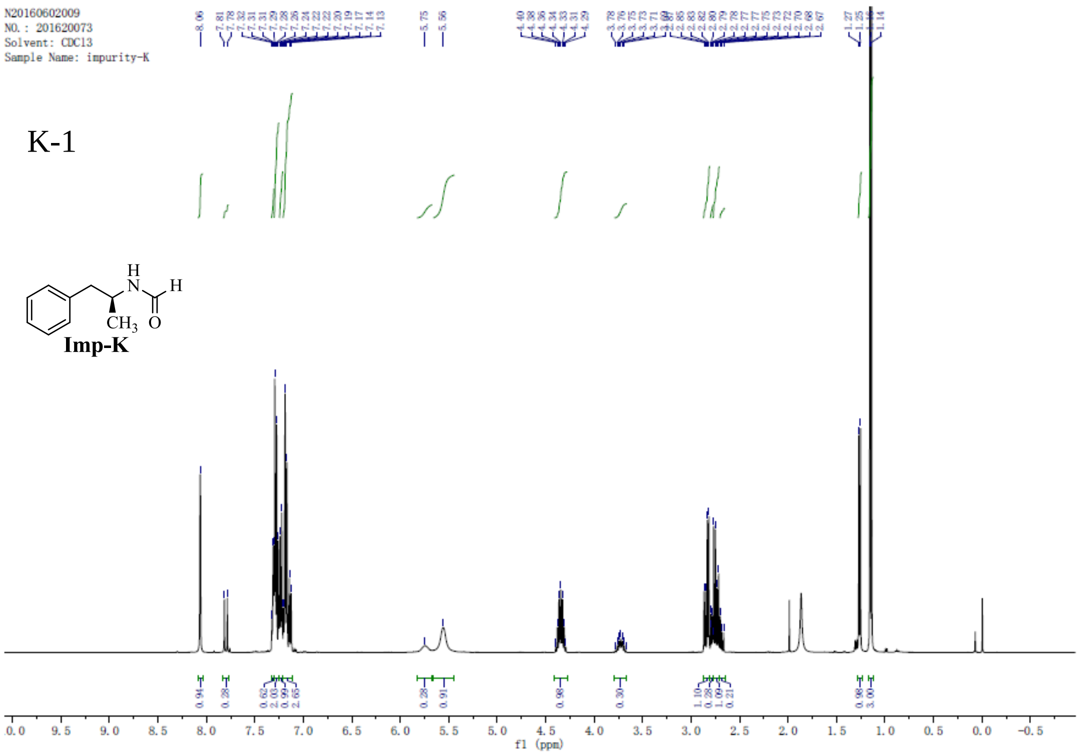

Imp-K ^{1}H NMR $CDCl_3$ 400 MHz

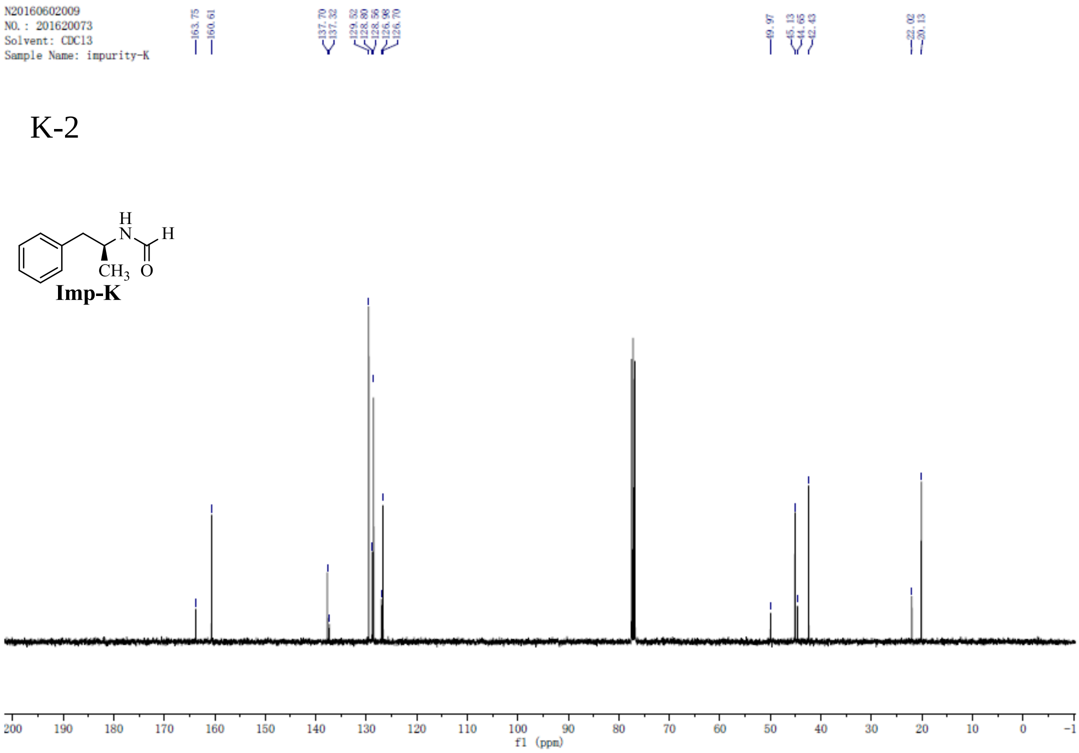

Imp-K ^{13}C NMR $CDCl_3$ 101 MHz

K-3

Qualitative Compound Report

**Data File**	20160706-003.d	**Sample Name**	Impurity-K
**Sample Type**	Sample	**Position**	Vial 77
**Instrument Name**	Instrument 1	**User Name**	
**Acq Method**		**IRM Calibration Status**	Success
**DA Method**	MS.m	**Comment**	N20160706004

Compound Table

Compound Label	RT	Mass	Abund	Formula	Tgt Mass	Diff (ppm)
Cpd 1: C10 H13 N O	0.14	163.0996	128172	C10 H13 N O	163.0997	-0.88

Compound Label	RT	Algorithm	Mass
Cpd 1: C10 H13 N O	0.14	Find By Formula	163.0996

MS Zoomed Spectrum

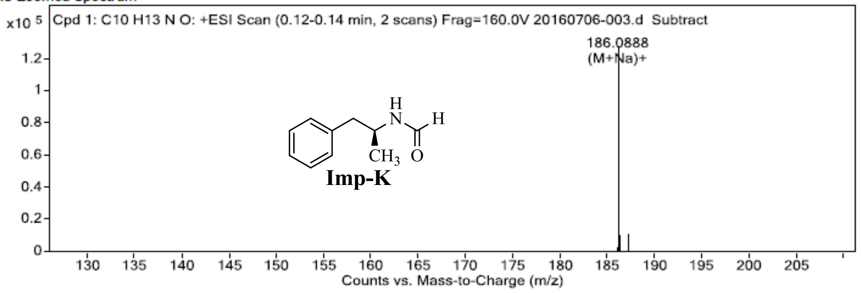

MS Spectrum Peak List

m/z	*Calc m/z*	Diff(ppm)	z	Abund	Formula	Ion
186.0888	186.0889	-0.77	1	128172	C10 H13 N Na O	(M+Na)+

--- End Of Report ---

Imp-K HRMS

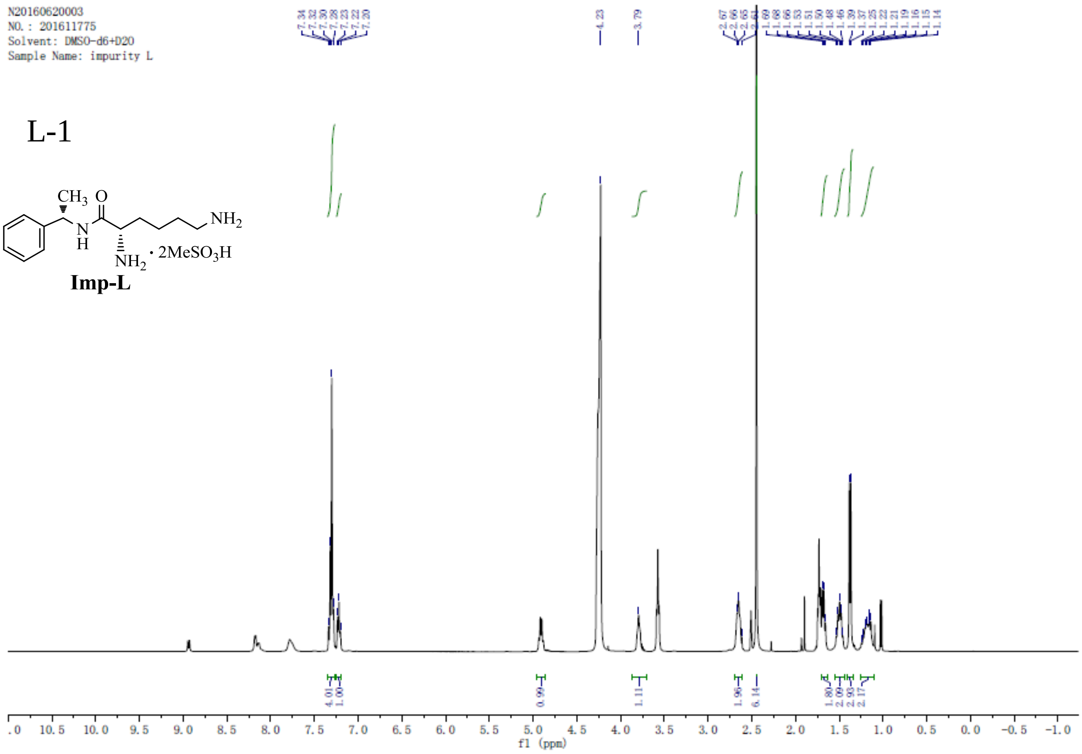

Imp-L ^{1}H NMR DMSO 400 MHz

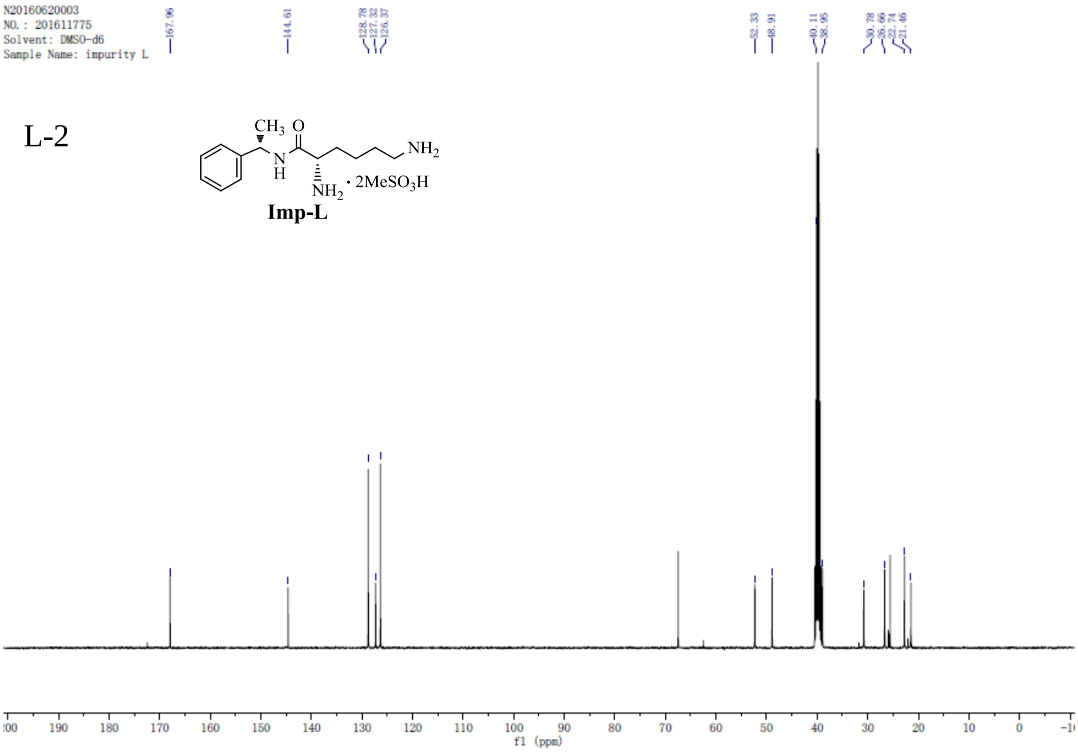

Imp-L ^{13}C NMR DMSO 101 MHz

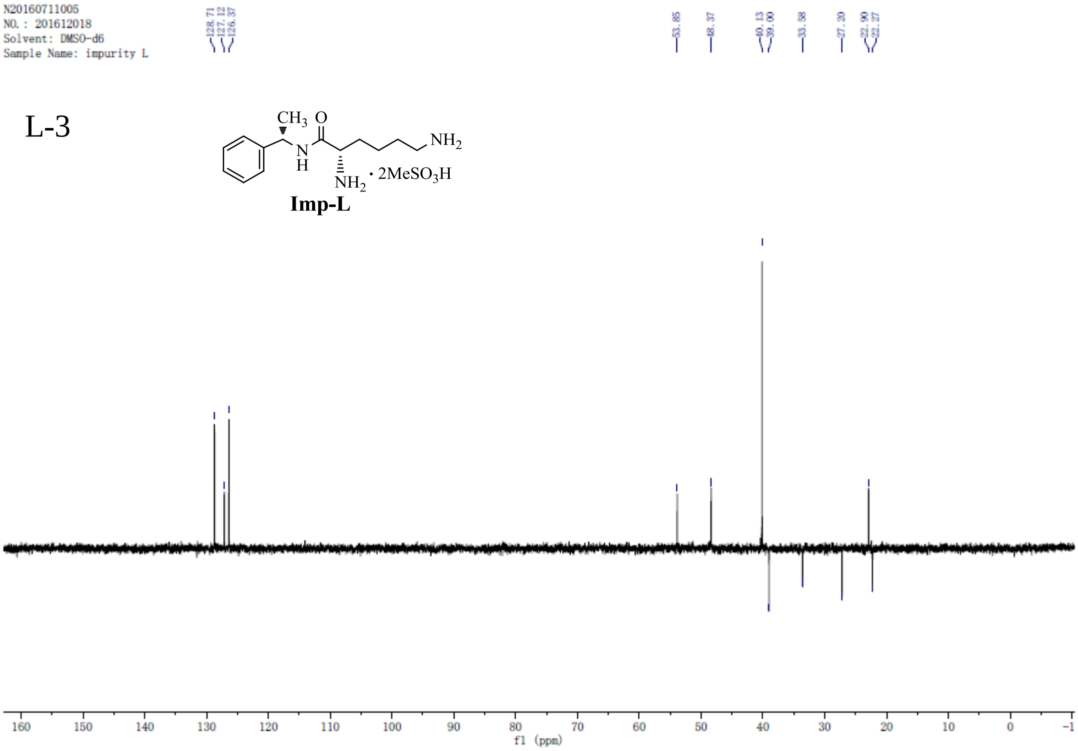

Imp-L DEPT DMSO 101 MHz

L-4

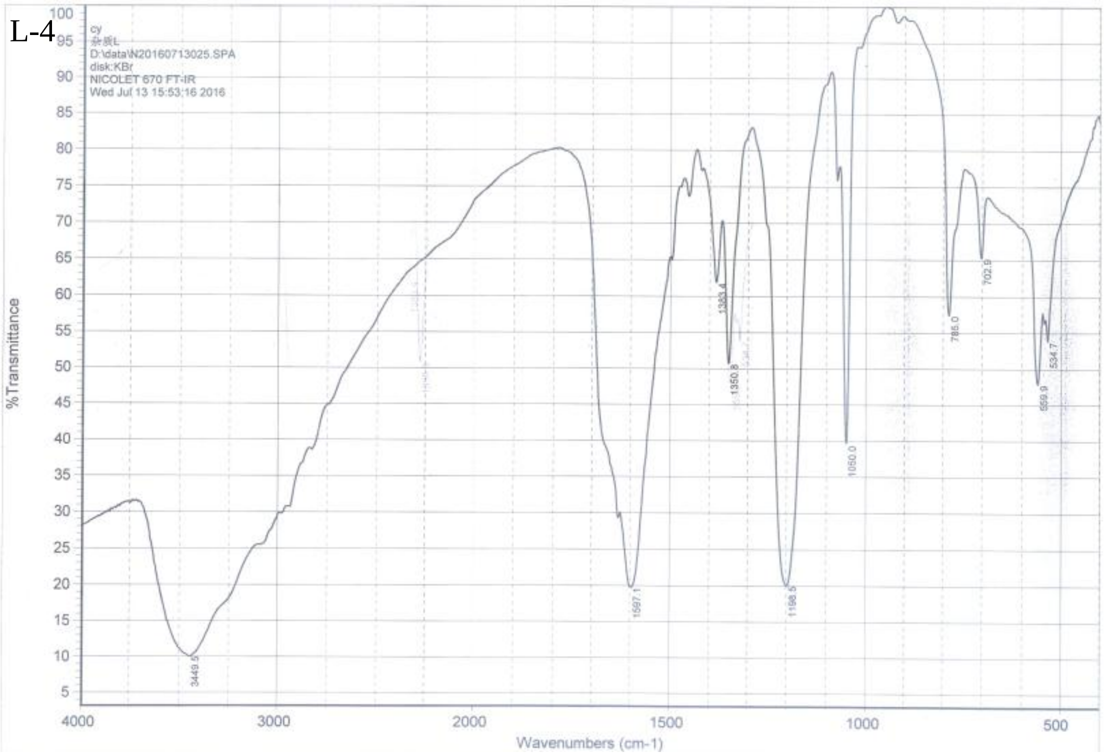

Imp-L IR

L-5

Qualitative Compound Report

**Data File**	20160630-012.d	**Sample Name**	Impurity L
**Sample Type**	Sample	**Position**	Vial 48
**Instrument Name**	Instrument 1	**User Name**	
**Acq Method**		**IRM Calibration Status**	Success
**DA Method**	MS.m	**Comment**	N20160630030

Compound Table

Compound Label	RT	Mass	Abund	Formula	Tgt Mass	Diff (ppm)
Cpd 1: C14 H23 N3 O	-0.25	249.1835	1176045	C14 H23 N3 O	249.1841	-2.39

Compound Label	RT	Algorithm	Mass
Cpd 1: C14 H23 N3 O	-0.25	Find By Formula	249.1835

MS Zoomed Spectrum

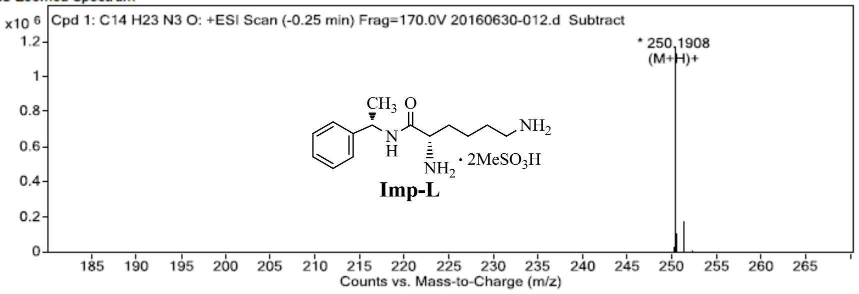

MS Spectrum Peak List

m/z	*Calc m/z*	Diff(ppm)	Abund	Formula	Ion
250.1908	250.1914	-2.37	1176045	C14 H24 N3 O	(M+H)+

--- End Of Report ---

Imp-L HRMS

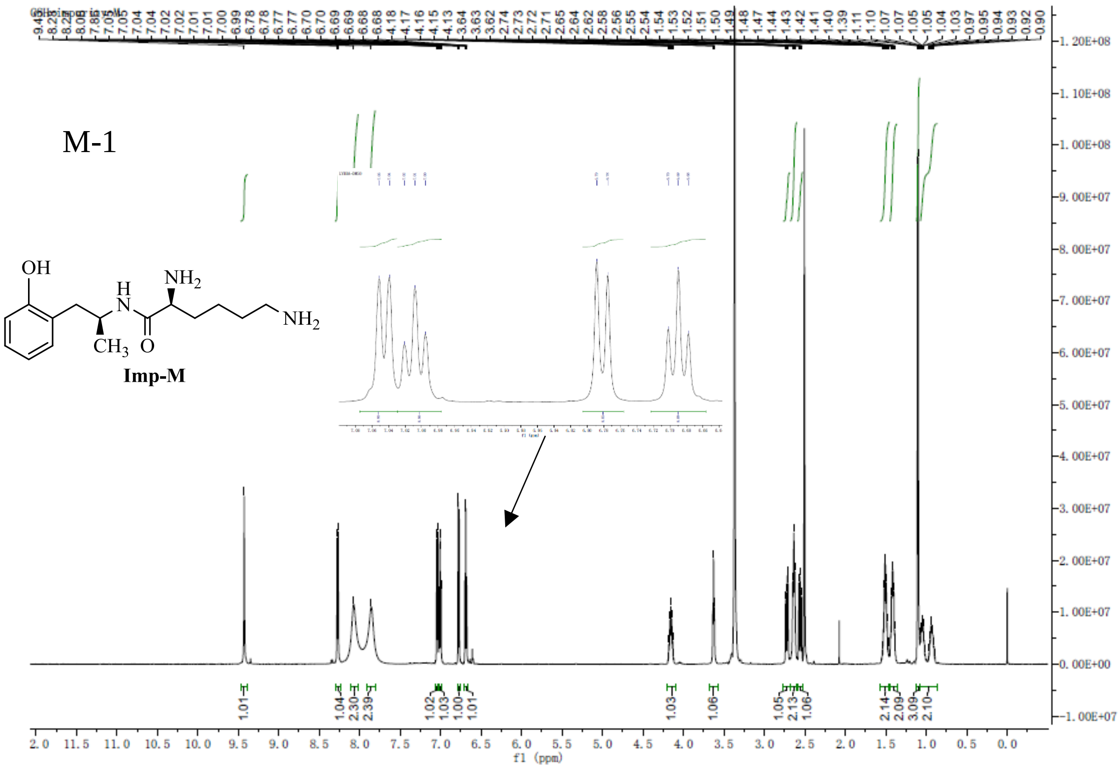

Imp-M ^{1}H NMR DMSO 400 MHz

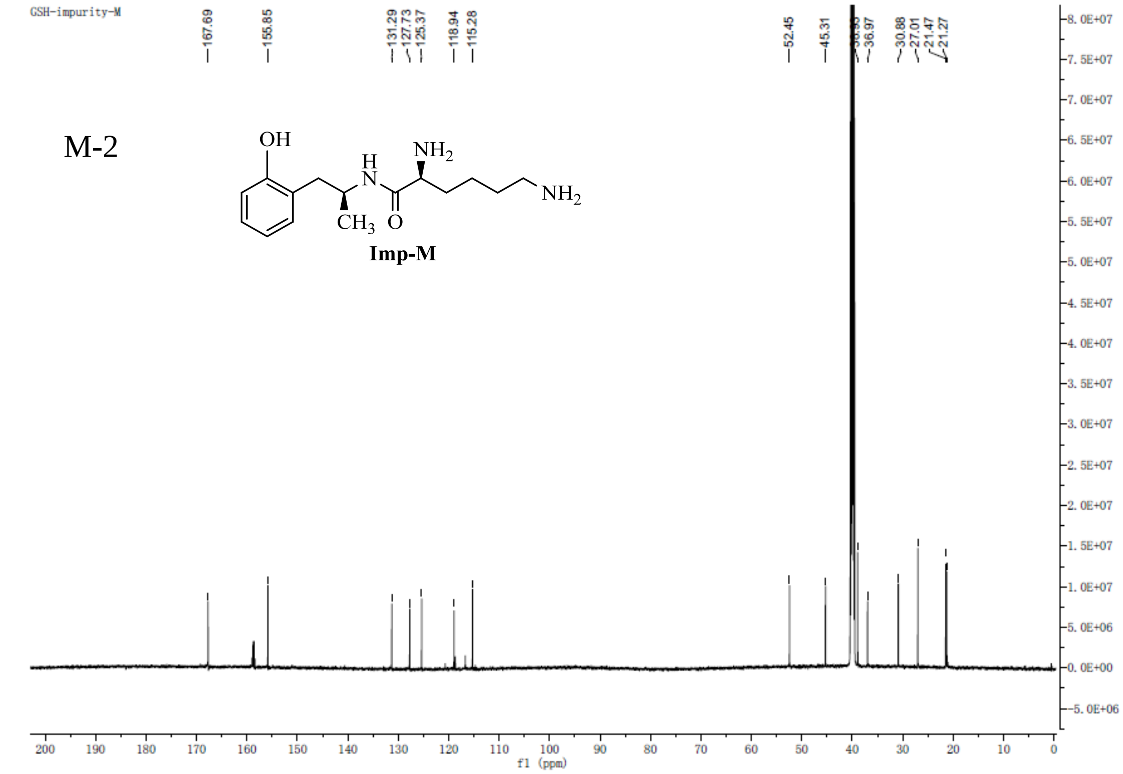

Imp-M ^{13}C NMR DMSO 101 MHz

M-3

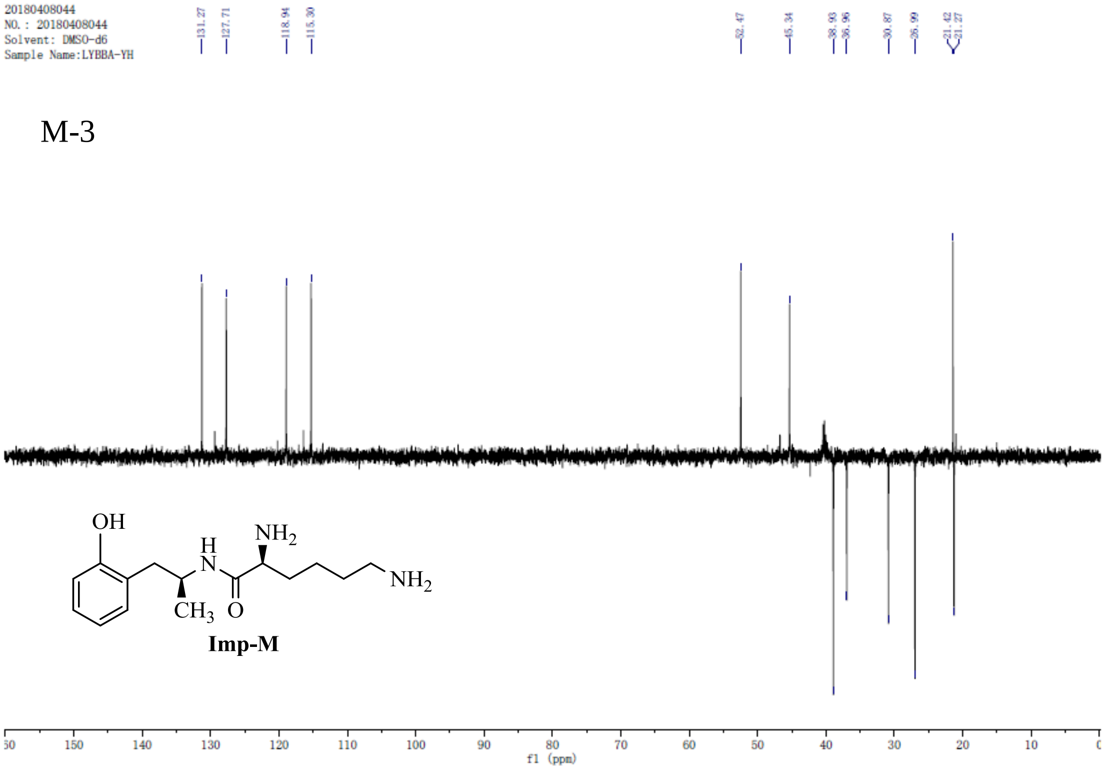

Imp-M DEPT DMSO 101 MHz

M-4

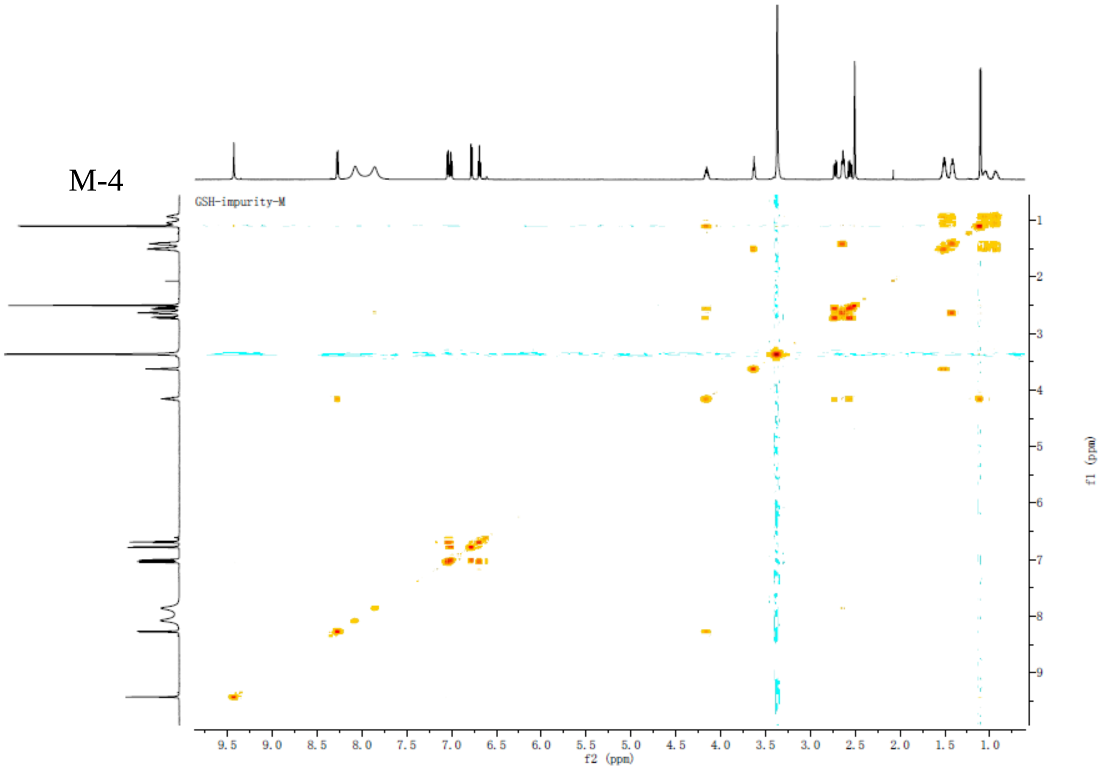

Imp-M COSY DMSO

M-5

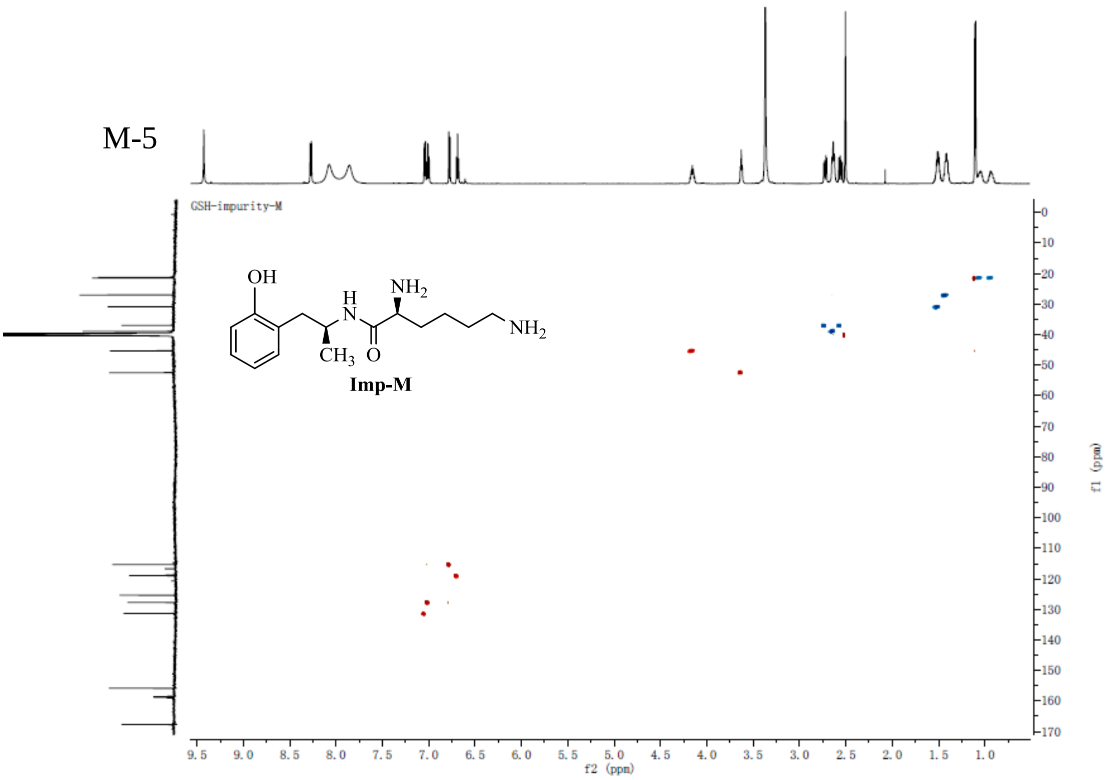

Imp-M HSQC DMSO

M-6

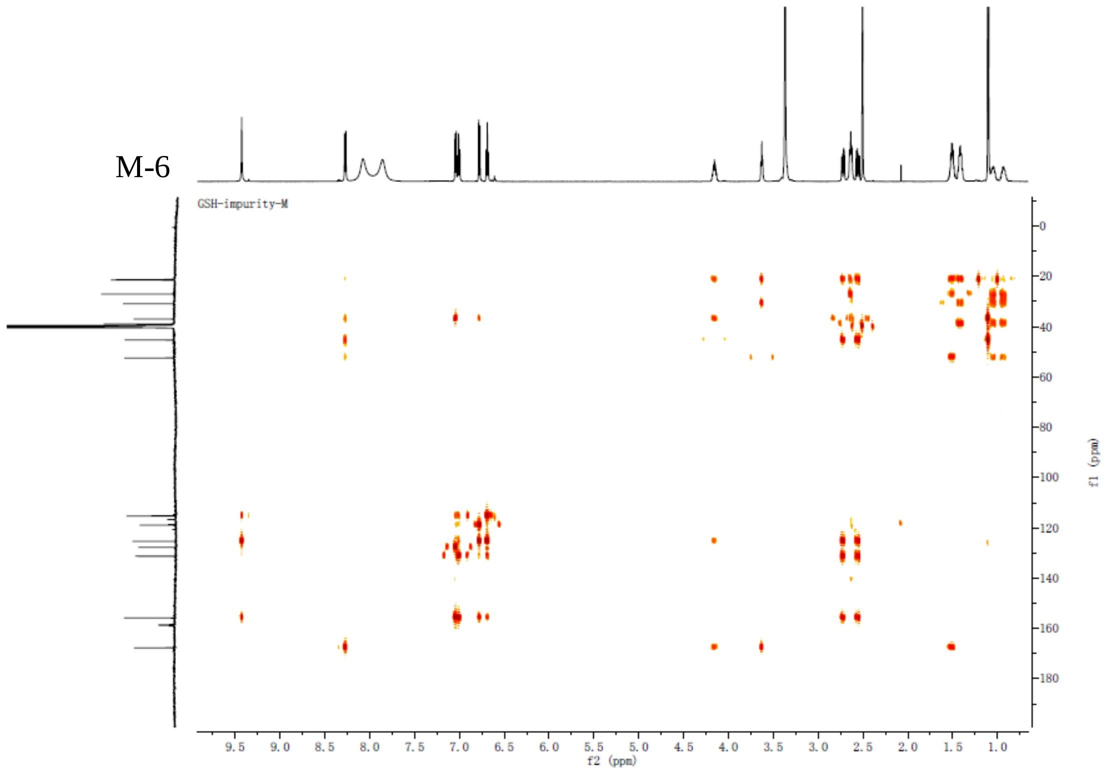

Imp-M HMBC DMSO

M-7

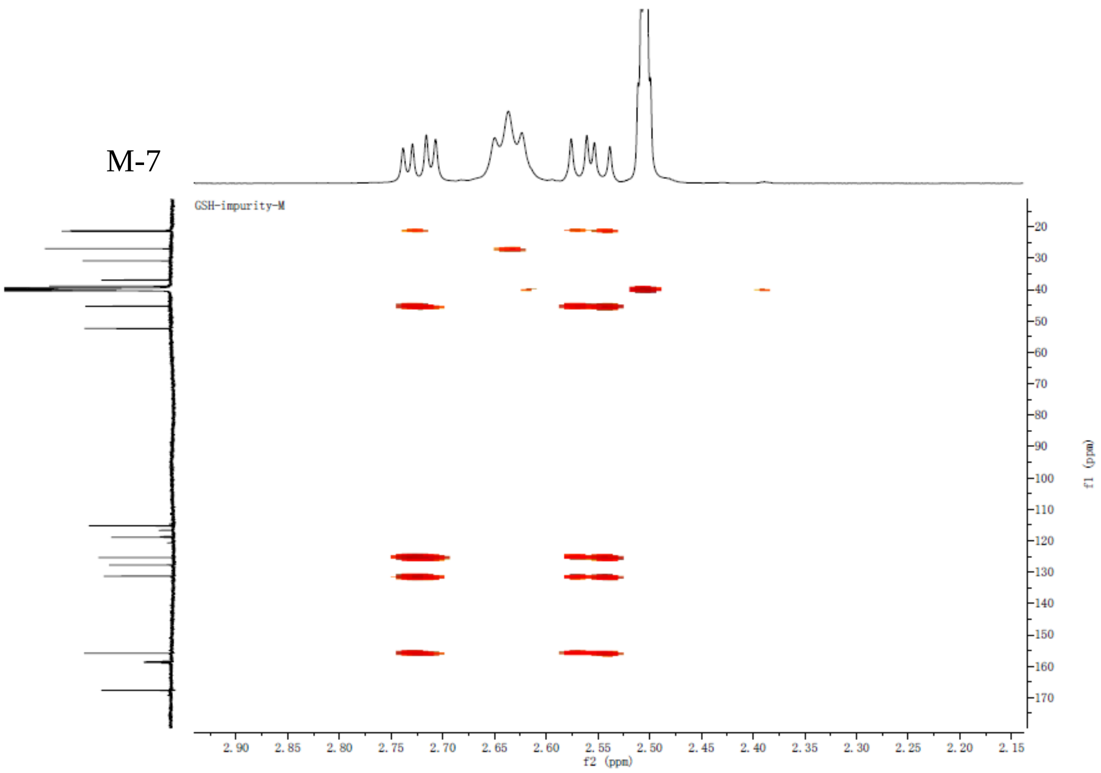

Imp-M the enlarged view of HMBC DMSO

M-8

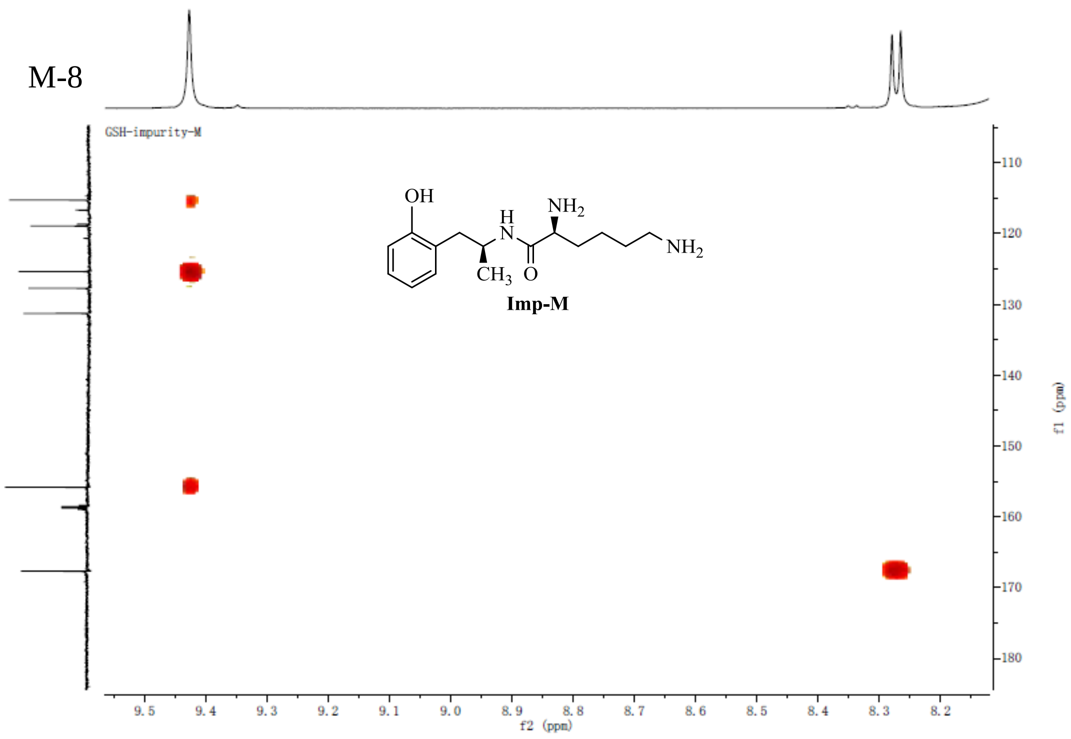

Imp-M the enlarged view of HMBC DMSO

M-9 **Mass Spectrum SmartFormula Report**

Analysis Info

Analysis Name	D:\Data\SHUJVFENXI\MADAWEIGROUP\LYBBA-2_GA3_01_10594.d	Acquisition Date	1/31/2018 5:53:48 AM
Method	20150915.m	Operator	BDAL@DE
Sample Name	LYBBA-2	Instrument / Ser#	maXis 4G 21240
Comment			

Acquisition Parameter

Source Type	ESI	Ion Polarity	Positive	Set Nebulizer	1.0 Bar
Focus	Not active	Set Capillary	4000 V	Set Dry Heater	220 °C
Scan Begin	50 m/z	Set End Plate Offset	-500 V	Set Dry Gas	6.0 l/min
Scan End	1500 m/z	Set Collision Cell RF	600.0 Vpp	Set Divert Valve	Waste

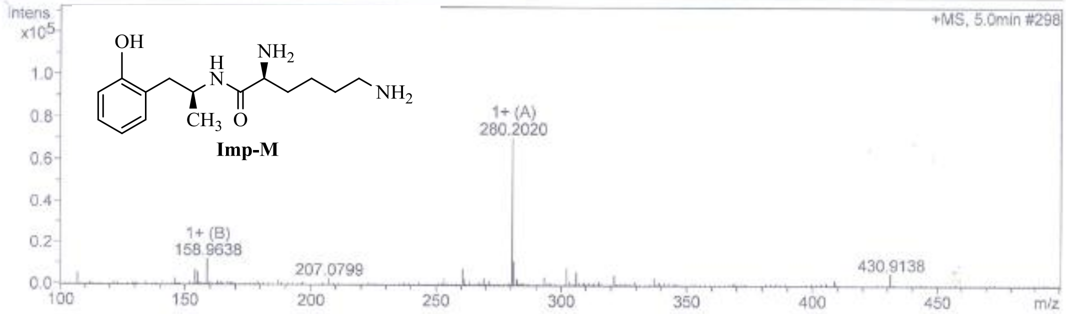

Meas. m/z	#	Formula	Score	m/z	err [ppm]	Mean err [ppm]	mSigma	rdb	e⁻ Conf	N-Rule
280.2020	1	C 15 H 26 N 3 O 2	100.00	280.2020	-0.3	-0.0	8.2	4.5	even	ok
	2	C 18 H 27 N Na	34.06	280.2036	5.5	5.8	22.6	5.5	even	ok
	3	C 10 H 24 N 8 Na	0.00	279.2016	5.9	10.2	767.3	2.5	even	ok
	4	C 13 H 28 N O 5	0.00	278.1962	-1.5	-5.6	812.7	0.5	even	ok
	5	C 14 H 24 N 5 O	0.00	278.1975	2.7	8.1	816.3	5.5	even	ok
	6	C 12 H 25 N 5 Na O	0.00	278.1951	-6.5	-0.5	816.3	2.5	even	ok

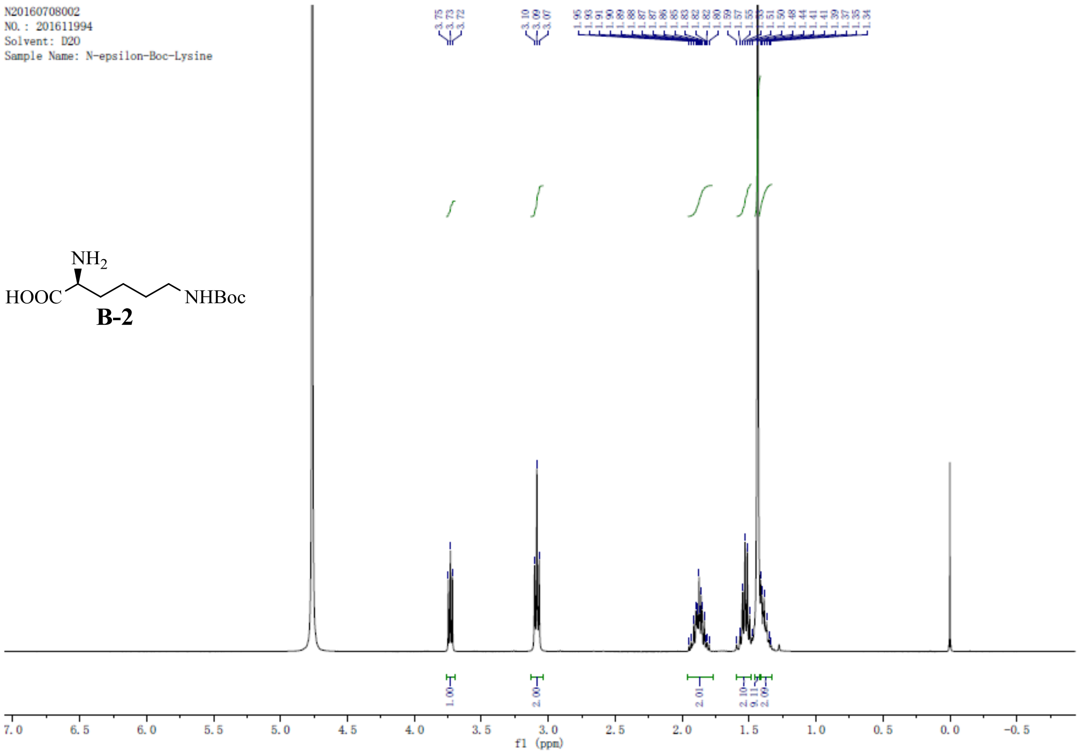

B-2 ^{1}H NMR D_2O 400 MHz

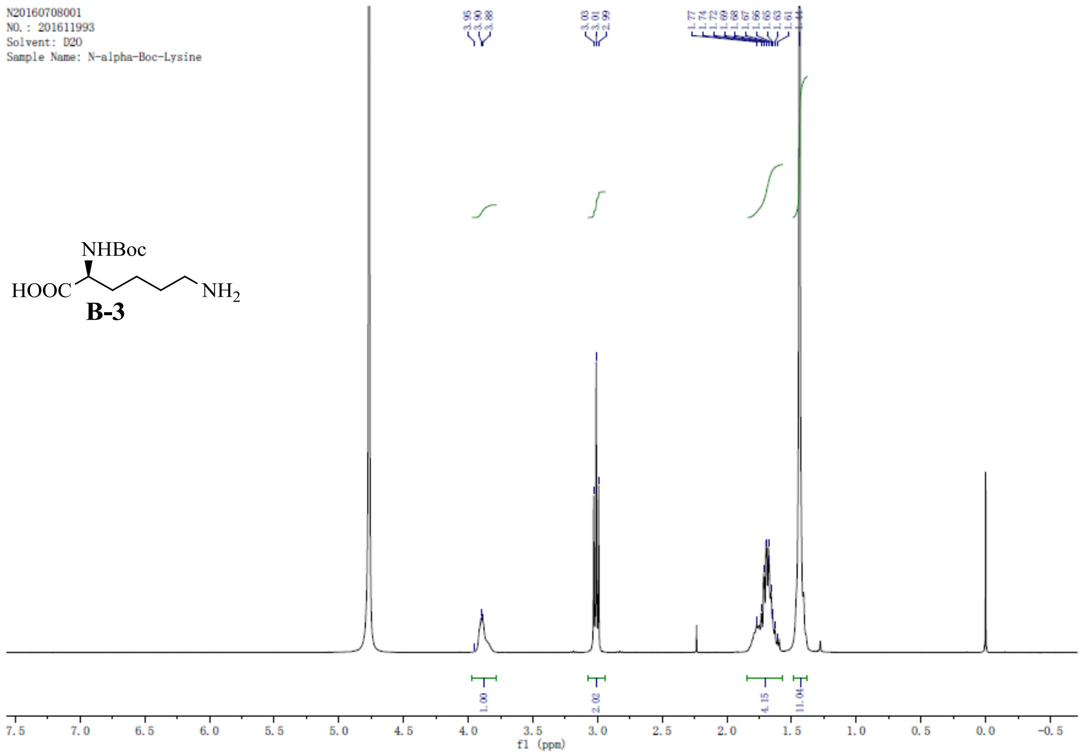

B-3 ^1H NMR D$_2$O 400 MHz
